# Comprehensive analysis of clinical *Burkholderia pseudomallei* isolates demonstrates conservation of unique lipid A structure and TLR4-dependent innate immune activation

**DOI:** 10.1371/journal.pntd.0006287

**Published:** 2018-02-23

**Authors:** Sineenart Sengyee, Sung Hwan Yoon, Suporn Paksanont, Thatcha Yimthin, Vanaporn Wuthiekanun, Direk Limmathurotsakul, T. Eoin West, Robert K. Ernst, Narisara Chantratita

**Affiliations:** 1 Department of Microbiology and Immunology, Faculty of Tropical Medicine, Mahidol University, Bangkok, Thailand; 2 Department of Microbial Pathogenesis, University of Maryland, Baltimore, MD, United States of America; 3 Mahidol-Oxford Tropical Medicine Research Unit, Faculty of Tropical Medicine, Mahidol University, Bangkok, Thailand; 4 Department of Tropical Hygiene, Faculty of Tropical Medicine, Mahidol University, Bangkok, Thailand; 5 Division of Pulmonary and Critical Care Medicine, Harborview Medical Center, University of Washington, Seattle, WA, United States of America; 6 International Respiratory and Severe Illness Center, University of Washington, Seattle, WA, United States of America; University of Tennessee, UNITED STATES

## Abstract

*Burkholderia pseudomallei* is an environmental bacterium that causes melioidosis, a major community-acquired infection in tropical regions. Melioidosis presents with a range of clinical symptoms, is often characterized by a robust inflammatory response, may relapse after treatment, and results in high mortality rates. Lipopolysaccharide (LPS) of *B*. *pseudomallei* is a potent immunostimulatory molecule comprised of lipid A, core, and O-polysaccharide (OPS) components. Four *B*. *pseudomallei* LPS types have been described based on SDS-PAGE patterns that represent the difference of OPS–type A, type B, type B_2_ and rough LPS. The majority of *B*. *pseudomallei* isolates are type A. We used matrix-assisted laser desorption/ionization time-of-flight mass spectrometry (MALDI-TOF MS) followed by electrospray ionization quadrupole time-of-flight mass spectrometry (ESI-QqTOF MS) and gas chromatography to characterize the lipid A of *B*. *pseudomallei* within LPS type A isolates. We determined that *B*. *pseudomallei* lipid A is represented by penta- and tetra-acylated species modified with 4-amino-4-deoxy-arabinose (Ara4N). The MALDI-TOF profiles from 171 clinical *B*. *pseudomallei* isolates, including 68 paired primary and relapse isolates and 35 within-host isolates were similar. We did not observe lipid A structural changes when the bacteria were cultured in different growth conditions. Dose-dependent NF-κB activation in HEK cells expressing TLR4 was observed using multiple heat-killed *B*. *pseudomallei* isolates and corresponding purified LPS. We demonstrated that TLR4-dependent NF-κB activation induced by heat-killed bacteria or LPS prepared from OPS deficient mutant was significantly greater than those induced by wild type *B*. *pseudomallei*. These findings suggest that the structure of *B*. *pseudomallei* lipid A is highly conserved in a wide variety of clinical and environmental circumstances but that the presence of OPS may modulate LPS-driven innate immune responses in melioidosis.

## Author summary

Melioidosis is a tropical infection caused by *Burkholderia pseudomallei*. Disease ranges from mild to lethal, and patients who die from melioidosis have greater inflammatory responses than survivors. Identifying variation in bacterial structures that alter the immune response is therefore important. *B*. *pseudomallei* expresses lipopolysaccharide, a major stimulator of the immune response. Studies of other bacteria suggest that differences in the lipid A component of lipopolysaccharide result in variable stimulation of the immune system. In this study, we analyzed the chemical structure of lipid A of 171 clinical *B*. *pseudomallei* isolates, structural changes caused by different growth conditions, and innate immune responses induced. Surprisingly, we did not observe any variation of lipid A structure or different innate immune activation from different *B*. *pseudomallei* isolates. Our data suggest that lipid A structure of *B*. *pseudomallei* is highly conserved and that different inflammatory responses in patients may be caused by other factors.

## Introduction

*Burkholderia pseudomallei* is a Gram-negative bacillus and the causative agent of melioidosis. This disease is endemic in Southeast Asia and northern Australia, with a mortality rate up to 40% in northeast Thailand and 20% in Australia [1, 2]. The manifestations of disease vary from indolent chronic infection to acute and fulminant sepsis. The common clinical presentations in melioidosis include severe pneumonia, bacteremia, skin infection, and abscesses in several organs [2]. *B*. *pseudomallei* is encapsulated, highly adaptable and able to survive under several extreme conditions in the environment, and clearance of *B*. *pseudomallei* in patients is difficult to achieve. The underlying mechanisms that contribute to persistence and death in melioidosis are unclear.

Acute melioidosis is characterized by the up-regulation of pattern recognition receptors (PRRs) and pro-inflammatory cytokine release [3]. Melioidosis patients have increased pro-inflammatory cytokines, such as IL-12, IL-15, IL-18 and IFN-γ and patients who die from melioidosis have higher levels of plasma IL-6 and IL-8 than those who survive [4]. These studies suggest the importance of the innate immune response in the control of infection and pathophysiology of sepsis and mortality in melioidosis.

Host immune cells express several PRRs including membrane bound Toll-like receptors (TLRs) and cytoplasmic receptors that recognize distinct bacterial pathogen-associated molecular patterns (PAMPs) [5]. Recognition of bacterial produces leads to the activation of innate immune response and release of inflammatory cytokines and mediators. Our previous study conducted in 300 healthy Thai donors suggest that lipopolysaccharide (LPS) of *B*. *pseudomallei* is a potent immuno-stimulatory molecule in humans [6]. The innate immune response to heat-killed *B*. *pseudomallei* is highly correlated with the response to purified LPS. We have also showed that genetic variation in TLR4, which encodes the canonical LPS receptor , TLR4 is associated with susceptibility to melioidosis [7]. These data point to the likely importance of *B*. *pseudomallei* LPS in the host-pathogen interaction in melioidosis.

Four *B*. *pseudomallei* LPS types have been described based on SDS-PAGE patterns which represent the difference of O-polysaccharides (OPS)–ladder type A, ladder type B, type B_2_ and rough LPS. Type A and B LPSs are structurally composed of three covalently linked domains of O-polysaccharide, a core-oligosaccharide and a lipid A moiety, while rough LPS lacks OPS. We previously reported that type A is the predominant LPS type expressed by 97% of *B*. *pseudomallei* isolates from Thailand and 80% of isolates from Australia [8]. Lipid A, the membrane anchor region of LPS is recognized by the host innate immune system. This process occurs by the presentation of LPS by LPS-binding protein (LBP) in concert with the accessory molecule CD14 to the TLR4/MD-2 complex [9]. This complex is present on a variety of cell types including macrophages and dendritic cells and upon binding of LPS, triggers the production of proinflammatory cytokines by host cells [10].

In several pathogenic Gram-negative bacteria, lipid A variation has been demonstrated both *in vivo* and *in vitro* to be associated with the induction of different innate immune responses. In *Salmonella enterica* serovar Typhimurium, the presence of Ara4N residues on phosphate groups occurs when the bacteria are grown under magnesium-deficient conditions. These modifications decrease the overall negative charge on the surface of bacteria resulting in the lower affinity for cationic antimicrobial peptides (CAMPs) [11]. Temperature-dependent lipid A modifications have been described in *Porphyromonas gingivalis*, *Francisella novicida*, and *Yersinia pestis* [12–14]. *Pseudomonas aeruginosa* isolates from patients with cystic fibrosis have different lipid A structures that are correlated with disease severity and progression [15, 16].

The first published study of *B*. *pseudomallei* lipid A structure in a single *B*. *pseudomallei* isolate, strain KHW, proposed a penta-acylated bisphosphorylated disaccharide backbone modified with Ara4N [17]. A second report on *B*. *pseudomallei* strains K96243 and 1026b showed the lipid A structure to be a predominantly tetra-acylated with a minor proportion of penta-acylated lipid A [18]. Recently, Norris *et al* have reported that LPSs from a type B Thai strain 576a and a virulent rough central nervous system-tropic strain MSHR435 from Australia induced higher innate immune responses than the prototype Thai type A strain 1026b. Matrix-assisted laser desorption/ionization tandem time-of-flight mass spectrometry (MALDI-TOF/TOF MS) analysis demonstrated structural differences in the lipid A of these isolates. They also suggested that the different LPS types may play a role in variable host responses to LPS [19] though a limitation of this study is the minimal number of *B*. *pseudomallei* used.

Due to the discrepancy in the findings of these reports, the first aim of this study was to characterize the lipid A structure in a large collection of clinical *B*. *pseudomallei* isolates using MALDI-TOF MS followed by electrospray ionization quadrupole- time-of-flight mass spectrometry (ESI-QqTOF MS) and gas chromatography. Because the vast majority of Thai and Australian patients infected with *B*. *pseudomallei* type A present with a wide range of clinical symptoms and incur different outcomes, we hypothesized that different innate immune responses to *B*. *pseudomallei* type A may be associated with variation in the lipid A structures of clinical isolates. The second aim was to examine the structural change of lipid A under varying laboratory conditions and the third aim was to evaluate the TLR4-dependent immuno-stimulatory effect of different *B*. *pseudomallei* type A isolates, as well as the role of OPS and capsule.

## Materials and methods

### Ethics statement

The study was exempted for ethical review by the Ethics Committee of the Faculty of Tropical Medicine, Mahidol University (documentary proof of exemption, MUTM-EXMPT 2017–003). All bacterial isolates obtained from human were anonymous.

### Bacterial strains and growth conditions

Cultivation of *B*. *pseudomallei* was performed in a biosafety level 3 (BSL3) laboratory. Two existing collections of *B*. *pseudomallei* isolates were used as follows: (i) 136 isolates from 68 melioidosis patients presenting to Sunpasitthiprasong Hospital, Ubon Ratchathani Northeast Thailand who developed relapsed infection. The relapse was defined as the bacterial isolates from the same patient during distinct episodes, which showed a similar pulsed-field gel electrophoresis banding pattern or multilocus sequence type regarded as originating from the same clone [20]. The first episode of melioidosis was between 1986 and 2004 and relapse was 15 days to 3,757 days after recovery from the primary episode (median = 207.5 days, IQR = 84.5–594.5) [20, 21]; (ii) 35 isolates from 35 individual colonies of 7 clinical specimens (5 colonies for each specimen) from a Thai patient with acute melioidosis who was admitted in 2006 to the same hospital [22]. The clinical specimens were blood, tracheal suction, urine, pus from right leg, pus from left leg, pus from forehead, and wound swab from thigh. Reference or laboratory strains of *B*. *pseudomallei* (K96243, 1026b, 153 and 164) were used for comparison. The *B*. *pseudomallei* wild type, OPS mutant and capsule mutant strains used during this study are described in S1 Table. Unless stated otherwise, *B*. *pseudomallei* was cultured on trypticase soy agar (TSA) and incubated at 37 ^o^C for 16–18 h. All isolates were stored in trypticase soy broth (TSB) with 15% glycerol at -80 ^o^C.

### Effect of different culture conditions on *B*. *pseudomallei* lipid A structure

The effect of a range of laboratory conditions on lipid A structure was tested for *B*. *pseudomallei* strain K96243. The bacteria were cultured on TSA (Oxoid, Hants, UK) for 16–18 h. Approximately 10 colonies were streaked onto agar plates and incubated for 16–18 h under one of the following conditions: (i) TSA (pH 7.4) at 25 ^o^C, (ii) TSA (pH 7.4) at 37 ^o^C, (iii) TSA (pH 7.4) at 42 ^o^C, (iv) TSA (pH 4.5) at 37 ^o^C, (v) TSA (pH 8.5) at 37 ^o^C, (vi) TSA (pH 7.4) plus 2 mM H_2_O_2_ (Merck, Darmstadt, Germany) at 37 ^o^C, (vii) TSA (pH 7.4) plus 5 mM H_2_O_2_ at 37 ^o^C, (viii) TSA (pH 7.4) plus 50 mM NaNO_2_ (Sigma-Aldrich, MO, USA) at 37 ^o^C, (ix) TSA (pH 7.4) plus 100 mM NaNO_2_ at 37 ^o^C, (x) TSA (pH 7.4) plus 1 mM MgCl_2_ (Fisher Scientific, Leics, UK) at 37 ^o^C, (xi) TSA (pH 7.4) plus 8 μM MgCl_2_ at 37 ^o^C, (xii) Luria-Bertani agar (LB) (BD, MD, USA) (pH 7.4) at 37 ^o^C, (xiii) Ashdown agar (pH 7.4) at 37 ^o^C, (xiv) blood agar (Oxoid, Hants, UK) (pH 7.4) at 37 ^o^C, (xv) MacConkey agar (Oxoid, Hants, UK) (pH 7.4) at 37 ^o^C, and (xvi) M9 minimal medium agar (Sigma-Aldrich, MO, USA) (pH 7.4) at 37 ^o^C. The bacteria were harvested using a 10 μl loop and further extracted for lipid A.

### Lipid A microextraction and MALDI mass spectrometry analysis

Lipid A was extracted from bacteria using a microextraction method as previously described [23] with some modifications. Briefly, the bacteria were cultured on TSA for 16–18 h and harvested as described above. One loop of the bacteria was suspended in 400 μl of 70% isobutyric acid (Sigma-Aldrich, MO, USA) and 1 M ammonium hydroxide (Sigma-Aldrich, MO, USA) at a ratio of 5:3 (v/v). The samples were heated at 100 ^o^C for 1 h followed by incubation on ice for 5 min. After centrifugation at 2000 × *g* for 15 min, the supernatant was collected, diluted with 1 ml of distilled water and lyophilized. The dried material was washed twice with 1 ml of methanol (Merck, Darmstadt, Germany) by centrifugation at 2000 × *g* for 15 min. The pellet was extracted for lipid A using 100 μl of a mixture of chloroform:methanol:water in a ratio of 3:1.5:0.25 (v/v/v). The suspension was centrifuged at 2000 × *g* for 1 min. One microliter of the supernatant was spotted onto a MALDI target plate and allowed to dry in air. The lipid A spots were overlaid with 1 μl of 20 mg/ml norharmane matrix (Sigma-Aldrich, MO, USA). Norharmane was used as a matrix in this study because it has been shown to improve limit of detection and support the characterization of a wide range of lipid A analysis [24]. Each spot was measured in 500 shot steps for a total of 3,000 laser shots using a Bruker Autoflex Speed MALDI-TOF mass spectrometer (Bruker Daltonics, Germany). The instrument was operated in negative ion reflector mode, across the mass range 1500–2000 and calibrated with electrospray ionization (ESI) tuning mix (Agilent Technologies, CA, USA). The mass spectrum was processed for smoothing and baseline subtraction using FlexAnalysis version 3.4 (Bruker Daltonics, Germany). Multiple spectra were displayed in a mass spectrum window for spectra comparison.

### Preparation of heat-killed bacteria

To prepare heat-killed *B*. *pseudomallei*, bacteria were grown on media including TSA, LB agar, Ashdown agar, blood agar, MacConkey agar and M9 minimal agar at 37 ^o^C in air for 16–18 h, harvested by obtaining two 10 μl loops and suspended in 1 ml of sterile phosphate-buffered saline (PBS) pH 7.4. The bacterial suspension was centrifuged at 10,000 × *g* for 10 min and washed twice with 1 ml of PBS. The pellet was resuspended in 1 ml PBS. One hundred microliters of bacterial suspension was taken to make a ten-fold serial dilution in PBS. The colony count was performed by spreading 100 μl of each dilution onto TSA plates in triplicate and incubating at 37 ^o^C for 16–18 h. The remaining bacterial suspension was heated at 80 ^o^C for 1 h. One hundred microliters of heat-killed bacteria were plated on TSA in duplicate for a sterility test.

### Purification of lipopolysaccharide

Lipopolysaccharide (LPS) was extracted using a modified hot water-phenol method as previously described [25]. Briefly, each of four *B*. *pseudomallei* isolates (strains K96243, 1026b, 153 and 164) and *B*. *pseudomallei* K96243 derivative with Δ*wbiD* (mutant defective in OPS synthesis) were cultured on TSA for 50 plates and incubated at 37 ^o^C for 16–18 h. Bacteria were scraped off using a 10 μl loop and suspended in 75 ml of distilled water (DW). The bacterial suspension was mixed with 90% phenol solution at a ratio of 1:1, heated at 80 ^o^C for 5 min with constant mixing, and subsequently cooled down at room temperature. The extract was dialyzed in a dialysis tube with pore size 6–8 kDa (Spectrum, CA, USA) against DW. The dialysate was centrifuged at 8,000 × *g* for 20 min and the supernatant was lyophilized. The dried material were solubilized in 10 mM Tris-HCl (pH 7.5) buffer containing 1 mM MgCl_2_, 1 mM CaCl_2_ at ratio of 30 mg:1 ml and sequentially treated with RNase and DNase at final concentration of 50 μg/ml at 37 ^o^C with agitation for 3 h, and proteinase K (Invitrogen, CA, USA) at final concentration of 50 μg/ml at 60 ^o^C for overnight. LPS was isolated from the supernatant by ultracentrifugation (Beckman Coulter) at 100,000 × *g* at 4 ^o^C for 3 h. The gel-like pellet was suspended in 10 ml pyrogen-free water and lyophilized. The purified LPS was further analyzed using SDS-PAGE and silver staining as previously described [8]. Protein contamination was examined using Coomassie blue staining [18] and bicinchoninic acid (BCA) assay (Pierce, IL, USA).

### Fatty acid analysis

The bacteria were cultured on 10 plates of TSA at 37 ^o^C for 16–18 h, harvested by 10 μl loop and suspended in 18 ml of PBS. Bacteria were inactivated by adding phenol to obtain 1% final concentration, and the bacterial suspension was incubated at 37 ^o^C for overnight. The cell pellet was collected by centrifugation at 12,000 × *g* for 3 min and wash twice with 20 ml of PBS. The pellet was resuspended in 5 ml PBS. Five hundred microliters of bacteria suspension were plated on TSA for sterility test. The remaining bacterial suspension was lyophilized and collected as a dried bacterial cell.

LPS fatty acids were derivatized to fatty acid methyl esters prior to gas chromatography (GC) analysis as previously described [26]. 20 mg of dried bacterial cells were suspended in 500 μl of DW. Five hundred microliters of 90% hot phenol (80 ^o^C) was added and further incubated at 70 ^o^C for 1 h with constant mixing. The cell suspension was incubated on ice 5 min and centrifuged at 10,000 × *g* for 10 min. The upper aqueous phase was obtained and washed with 2 ml of diethyl ether (Fisher Scientific, NJ, USA) by centrifugation at 3,000 × *g* for 5 min. Then, the lower aqueous phase containing LPS was collected and lyophilized. The 200 μg of dried LPS were treated with 200 μl of 2 M anhydrous methanolic HCL (Alltech, KY, USA) at 90 ^o^C for 18 h. Then, the derivatized fatty acid methyl ester was cooled down at room temperature. Two hundred microliters saturated NaCl solution was added. The mixture was subsequently extracted with 400 μl of hexane (Sigma-Aldrich, MO, USA) by mixing using a vortex for 30 sec. The upper layer phase (~400 μl) was analyzed by a gas chromatography-flame ionization detector (GC-FID), HP 5890 series II with a 7673 autoinjector. Bacterial acid methyl ester CP mixture (Matreya, PA, USA) was used as standard control and pentadecanoic acid (Sigma Aldrich, MO, USA) was used as an internal control respectively.

### Lipid A hydrolysis and ESI tandem mass spectrometry analysis

Lipid A was liberated from purified LPS by hydrolysis. Purified LPS was dissolved in 10 mM sodium acetate (pH 4.5) with 1% SDS and then heated at 100 ^o^C for 1 h. The sample was lyophilized and then washed with 170 μl of endotoxin free water and 850 μl of acidified ethanol mixture by centrifugation at 5,000 × *g* for 5 min. The sample was further washed with 1 ml of non-acidified 95% ethanol and followed by 1 ml of 100% ethanol. Finally the sample was lyophilized to yield lipid A. The structural characterization was analyzed on a Waters Synapt G2 QqTOF mass spectrometer (Waters Corporation, Milford, MA, USA) operated in sensitivity mode and negative ion mode. The lipid A was dissolved in a solvent mixture of chloroform and methanol (2:1 v/v). The lipid solution was infused at 3 μl/min flow rate. The source block temperature was set to 150 ^o^C. Tandem MS was carried out using trap collision induced dissection (CID). The instrument standard values (LM resolution 4.7 and HM resolution 15.0) were used for mass selection in the quadrupole. To mitigate the effect of instantaneous signal fluctuations, the data were averaged for 2–3 min.

### Cell culture conditions and stimulations

TLR4-transfected human embryonic kidney cells (HEK-Blue^TM^-hTLR4 cells) were purchased from InvivoGen (San Diego, CA, USA). The HEK-Blue^TM^-hTLR4 cells are HEK cells that are stably transfected with human TLR4 (hTLR4), myeloid differentiation factor-2, cluster of differentiation-14 (MD-2/CD14) and a secreted embryonic alkaline phosphatase (SEAP) reporter gene. The cells were cultured and maintained at 37 ^o^C with 5% CO_2_ in complete Dulbecco’s modified Eagle medium (DMEM) (Gibco, NY, USA) which contained 10% heat inactivated fetal bovine serum (FBS) and 1× HEK-Blue selection medium (InvivoGen, San Diego, CA, U.S.A); an antibiotic mixture for maintenance of HEK-Blue hTLR4 cell lines. The cells were seeded at 2.5 × 10^4^ cells/well in a 96-well plate and stimulated for 24 h with heat-killed bacteria at 10^6^ or 10^7^ CFU/ml and purified LPS at 1, 10, 100, 1000, 10,000 ng/ml from *B*. *pseudomallei* isolates. Cells stimulated with ultrapure TLR4 ligand *Escherichia coli* O111:B4 LPS (Sigma-Aldrich, MO, USA) was a positive control. The activation of nuclear transcription factor (NF)-κB in HEK-Blue hTLR4 cells in response to TLR4 agonists was determined by a SEAP reporter assay at a wavelength 620 nm using a microplate reader (TECAN, Grodig, Austria). The results of NF-κB activation in HEK-Blue hTLR4 cells were calculated to account for the difference in weight of wild type LPS and *ΔwbiD* mutant LPS by a factor 2.5. The mass individually of the lipid A (1948), core (1258), and repeating O-antigen unit (423+ 4250 = 4673) were used to calculated the weight of wild type LPS. The mass individually of the lipid A (1948) and core (1258) were used to calculated the weight of the mutant LPS.

### Antimicrobial susceptibility to polymyxin B testing

Susceptibility testing to polymyxin B was performed with *B*. *pseudomallei* clinical isolates using a broth microdilution method according to the Clinical and Laboratory Standards Institute (CLSI) guidelines [27]. The bacterial isolates used in this experiment were randomly selected from different groups of infections that the lipid A results showed slightly different mass intensity at m/z 1801.6. These included 14 isolates from 7 Thai patients with primary infection (strains: 226a, 855e, 1234a, 1304a, 1620a, 1790a and 2944b) and relapse after recovery from primary infection (strains: 240a, 855h, 1234b, 1304b, 1620d, 1790b and 2944d) and 7 isolates from within-host infection [H3921b(C5), H3921c(C3), H3921d(C2), H3921e(C2), H3921f(C2), H3921g(C3), H3921h(C5)]. The MIC interpretive criteria used for polymyxin B were established in CLSI standard M100-S24 as follows: susceptible ≤ 2 μg/ml, intermediate 4 μg/ml and resistant ≥ 8 μg/ml [28]. *Pseudomonas aeruginosa* ATCC 27853 was used as a control strain. *B*. *pseudomallei* isolates were recovered from the freezer vials at -80 ^o^C by streaking onto Columbia agar and incubating aerobically at 37 ^o^C for 24 h. Bacterial colonies were harvested and suspended in normal saline 0.85%. Bacteria suspension was adjusted at OD_600 nm_ to obtain a concentration of 1 × 10^8^ CFU/ml. Bacteria at a final concentration of 5 × 10^5^ CFU/ml as recommended in CLSI document M07-A9 [29] were used for susceptibility testing of polymyxin B (catalog number P4932; Sigma-Aldrich) at concentrations of 0, 0.25, 0.5, 1, 2, 4, 8, 16, 32, 64, 128, 256, and 512 μg/ml in duplicate. The MIC was read as the lowest drug concentration at which no visible growth was observed following aerobic incubation at 35 ± 2 ^o^C for 16–20 h.

### Construction of mutants

Four *B*. *pseudomallei* mutants including three OPS mutants and a capsule mutant were constructed from wild types K96243 and 4095A, respectively using a fragment mutagenesis method as described [30] (S1 Table). K96243*ΔwbiD* was defective in *wbiD* and did not synthesize the OPS, K96243*ΔwbiA* and K96243*ΔoacA* were both defective in acetylation of the OPS. The capsule mutant (4095a *ΔwcbB*) was defective in *wcbB* and did not have capsule [30]. Characterization of OPS was examined by SDS-PAGE of proteinase K extracts, silver staining and Western blot using a monoclonal antibody to OPS [8]. Characterization of capsule was examined by latex agglutination and Western blot using a monoclonal antibody to CPS [31].

### Statistical analysis

Statistical analysis was performed using GraphPad Prism (version 5; GraphPad Software). One-way ANOVA was used to test the difference among the means of NF-κB activation from different *B*. *pseudomallei* isolates. Unpaired *t* tests were used to test the difference between the mean of results from *B*. *pseudomallei* wild type and mutant strains. The data were presented as mean ± standard deviations. Differences were considered statistically significant at a *P* value < 0.05.

## Results

### MALDI-TOF MS analysis of *B*. *pseudomallei* K96243 lipid A

**Fig 1:**
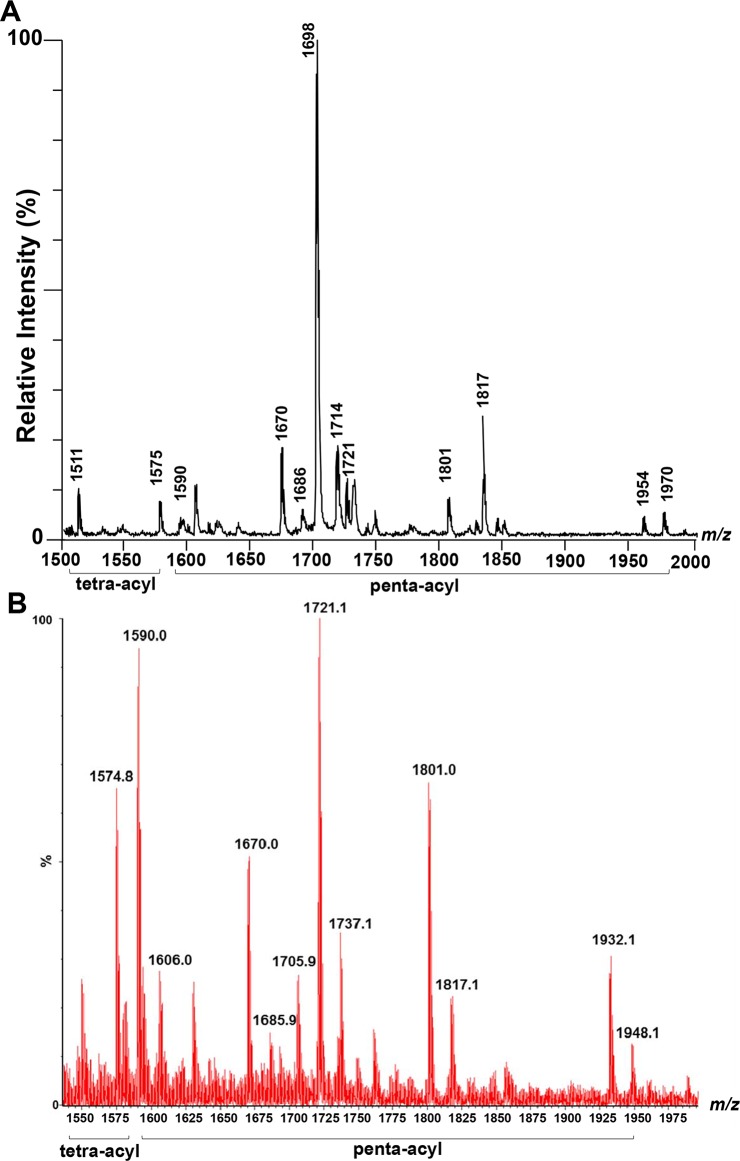
Negative ion mass spectrum by MALDI-TOF (A), by ESI (B) of *B*. *pseudomallei* K96243 lipid A.

**Fig 2:**
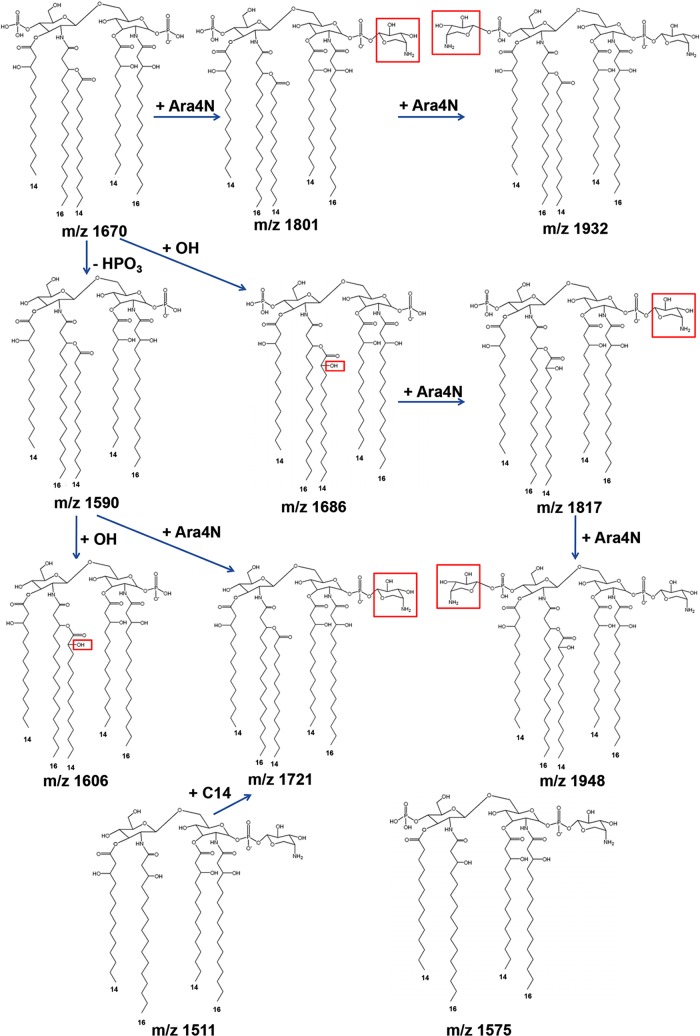
Predicted structure of *B*. *pseudomallei* K96243 lipid A.

We initially analyzed the lipid A isolated from the reference strain *B*. *pseudomallei* K96243. Negative ion mass spectra of K96243 lipid A showed a complex pattern of ions with major ions at m/z 1511, 1575, 1590, 1670, 1686, 1698, 1714, 1721, 1801, 1817, 1954, 1970 (Fig 1A). The predicted structures of fatty acid, phosphate, and carbohydrate substituents on lipid A of K96243 backbone are shown in Fig 2 and Table 1. Based on overall chemical compositions, the data predicted that *B*. *pseudomallei* contained penta- and tetra-acylated lipid A species. The ion at m/z 1511 was a representative of tetra-acylated monophosphorylated GlcN disaccharide backbone possessing two C14:0(3-OH) residue in ester linkage and two C16:0(3-OH) residues in amide linkage with one phosphorylated and one 4-amino-4-deoxy-arabinose (Ara4N) attached to the phosphate group. The ion at m/z 1575 was a representative of tetra-acylated bisphosphorylated GlcN disaccharide backbone possessing one C14:0 residue and one C14:0(3-OH) residue in ester linkage and two C16:0(3-OH) residues in amide linkage with two phosphorylated and one Ara4N attached to the phosphate group. The ion at m/z 1590 was a penta-acylated monophosphorylated GlcN disaccharide backbone possessing one C14:0, two C14:0(3-OH) and two C16:0(3-OH) residues with one phosphorylated. The ion at m/z 1670 was a penta-acylated bisphosphorylated GlcN disaccharide backbone possessing one C14:0 residue, two C14:0(3-OH) residues and two C16:0(3-OH) residues with two phosphorylated. The ion at m/z 1686 was a representative of ion at m/z 1670 with the substitution of fatty acid C14:0 for C14:0(2-OH). The ion at m/z 1721 was a representative of ion at m/z 1590 modified with one Ara4N residue attached to the phosphate group (*Δ*m/z + 131). The ion at m/z 1801 was a representative of ion at m/z 1670 modified with an Ara4N residue attached to the phosphate group. The ion at m/z 1817 was a representative of ion at m/z 1686 modified with an Ara4N residue attached to the phosphate group. The ion at m/z 1954 was a representative of ion at m/z 1670 with sodium adduct and modified with two Ara4N residues attached to the phosphate group. The ion at m/z 1970 was a representative of ion at m/z 1686 with sodium adduct and modified with two Ara4N residues attached to the phosphate group. The ions at m/z 1698 and 1714 were detected only by MALDI-TOF and could not be detected for structural identification by ESI-QqTOF.

**Table 1:** Negative ion MALDI-TOF and ESI-QqTOF MS lipid A species and predicted structures of the fatty acid, phosphate and carbohydrate substituents on the *B*. *pseudomallei* K96243 lipid A backbone.

Lipid A species	Observed ion (m/z)	Theoretical monoisotopic ion (m/z)	Acyl substitution	Predicted structure
1	1511.7	1511.0	Tetra	2 × C14:0(3-OH), 2 × C16:0(3-OH), P, Ara4N
2	1574.8	1575.0	Tetra	C14:0, C14:0(3-OH), 2 × C16:0(3-OH), 2 × P, Ara4N
3	1590.0	1590.1	Penta	C14:0, 2 × C14:0(3-OH), 2 × C16:0(3-OH), P
4	1606.0	1606.1	Penta	C14:0(2-OH), 2 × C14:0(3-OH), 2 × C16:0(3-OH), P
5	1670.0	1670.1	Penta	C14:0, 2 × C14:0(3-OH), 2 × C16:0(3-OH), 2 × P
6	1685.9	1686.1	Penta	C14:0(2-OH), 2 × C14:0(3-OH), 2 × C16:0(3-OH), 2 × P
7	1721.1	1721.2	Penta	C14:0, 2 × C14:0(3-OH), 2 × C16:0(3-OH), P, Ara4N
8	1801.0	1801.1	Penta	C14:0, 2 × C14:0(3-OH), 2 × C16:0(3-OH), 2 × P, Ara4N
9	1817.1	1817.2	Penta	C14:0(2-OH), 2 ×C14:0(3-OH), 2 × C16:0(3-OH), 2 × P, Ara4N
10	1932.1	1932.2	Penta	C14:0, 2 × C14:0(3-OH), 2 × C16:0(3-OH), 2 × P, 2 × Ara4N
11	1948.1	1948.2	Penta	C14:0(2-OH), 2 ×C14:0(3-OH), 2 × C16:0(3-OH), 2 × P, 2 × Ara4N

**Fig 3:**
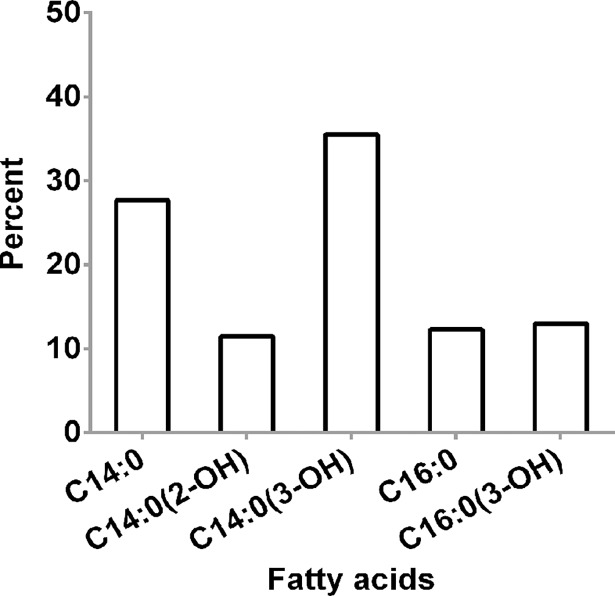
Fatty acid composition of *B*. *pseudomallei* K96243 lipid A.

The total fatty acid of *B*. *pseudomallei* K96243 lipid A was validated by gas chromatography (GC). The result is presented in Fig 3. Fatty acid composition of *B*. *pseudomallei* strain K96243 LPS confirmed the MALDI-TOF MS results for the presence of tetradecanoic acid (C14:0), 2-hydroxytetradecanoic [C14:0(2-OH)], 3-hydroxytetradecanoic acid [C14:0(3-OH)], hexadecanoic acid (C16:0), and 3-hydroxyhexadecanoic acid [C16:0(3-OH)]. Our data suggest that the lipid A species of *B*. *pseudomallei* K96243 was predominantly penta-acylated with a combination of C14:0(2-OH), C16:0(3-OH), C14:0(3-OH) or C14:0.

### ESI-QqTOF analysis of *B*. *pseudomallei* K96243 lipid A

ESI-QqTOF MS analysis with negative ion mode of *B*. *pseudomallei* K96243 lipid A showed major ions at m/z 1574.8, 1590.0, 1606.0, 1670.0, 1685.9, 1721.1, 1801.0, 1817.1, 1932.1 and 1948.1 (Fig 1B). ESI results, where ESI-QqTOF gives higher mass accuracy, as compared to MALDI-TOF are shown at Fig 1B. The two MS results are very reproducible. These ions were further characterized by trap collision-induced dissociation (S1 Fig) and the tandem MS results confirm the predicted structures (Fig 2). The ion at m/z 1606.0 was a representative of m/z 1590.0 with the substitution of fatty acid C14:0 for C14:0(2-OH). The ion at m/z 1932.1 was a representative of ion at m/z 1670.0 with two Ara4N residues. The m/z 1698 and 1714 were detected only by MALDI-TOF and could not be detected for structural identification by ESI-QqTOF.

### Mass spectra of lipid A from clinical isolates of *B*. *pseudomallei*

**Fig 4:**
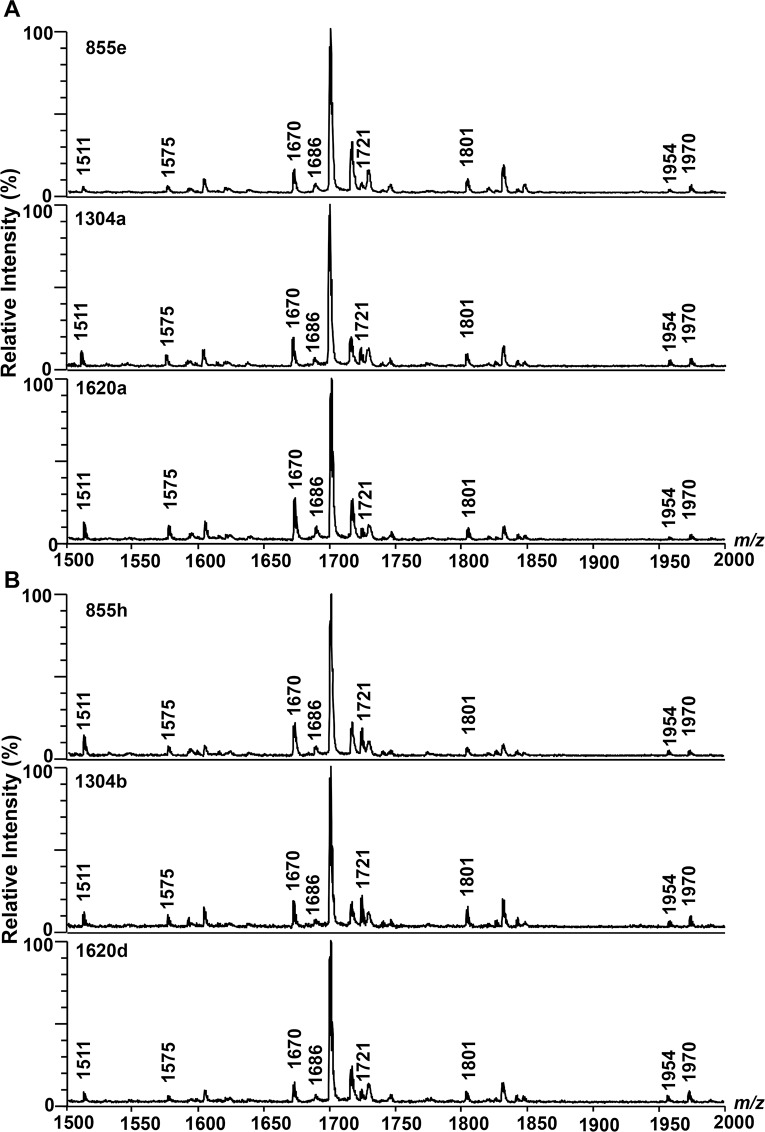
MALDI-TOF lipid A spectra of clinical isolates of *B*. *pseudomallei* strains from three Thai patients with primary infection followed by an episode of relapse. (A) Primary isolates: 855e, 1304a and 1620a. (B) Relapse isolates: 855h, 1304b and 1620d.

We next investigated the variation of lipid A structure in 136 clinical isolates of *B*. *pseudomallei* grown on TSA plates. The mass spectrum of lipid A was determined in two groups of existing Thai isolate collections. The first group represented primary isolates (S2 Fig) and relapse isolates (S3 Fig) from 68 melioidosis patients who had at least one episode of relapse. MALDI-TOF lipid A spectra of representatives of these isolates are shown in Fig 4. Using a negative reflector mode MALDI-TOF MS analysis, we observed similar lipid A spectra in a mass range of 1500–2000 Da in all isolates from both primary and relapse infections. All lipid A species of these isolates contained four main clusters of peaks around *m/z* of 1575, 1670, 1801 and 1954 corresponding to tetra-acylated modified with one phosphorylated and one Ara4N group, bisphosphorylated penta-acylated, bisphosphorylated penta-acylated modified with only one Ara4N group and bisphosphorylated penta-acylated modified with two Ara4N group, respectively. The second group consisted of 35 *B*. *pseudomallei* isolates from a variety of specimen type (blood, tracheal suction, urine, pus from right leg, pus from left leg, pus from forehead, and wound swab from thigh) of a Thai patient with acute melioidosis. The lipid A species of these within-host isolates were not different (S4 Fig), and represented the same four main clusters of peaks at approximately *m/z* of 1575, 1670, 1801 and 1954. Together, these comprehensive set of data indicate a striking lack of variation in lipid A structure in a large group of clinical isolates cultured from patients with primary melioidosis, as well as in isolates obtained during relapse infection, and from multiple isolates obtained from numerous sites within a single host.

### Effect of laboratory culture condition on *B*. *pseudomallei* K96243 lipid A structure

We considered the possibility that the lipid A structures of *B*. *pseudomallei* isolates may be modified during growth on TSA at 37 ^o^C, the conditions used in the initial experiments. To address this, we tested whether the lipid A structure of *B*. *pseudomallei* K96243 differed following culture under a range of different laboratory conditions. All of lipid A spectra of *B*. *pseudomallei* cultured in different laboratory conditions had the same four main clusters of peaks at approximately *m/z* of 1575, 1670, 1801 and 1954 (S5 Fig). These data indicate that *B*. *pseudomallei* K96243 lipid A molecule was structurally conserved and suggested that the lack of variability in lipid A structure observed in our 136 clinical isolates was not a result of identical growth conditions.

### Susceptibility of *B*. *pseudomallei* to polymyxin B

Cationic antimicrobial peptides (CAMPs), such as polymyxin B exerts their activity through electrostatic interactions with the lipid A moiety of LPS causing disruption of the outer membrane and cell death. Bacteria can develop resistance to colistin by modifying the structures of the lipid A moiety, hindering colistin binding. Aminoarabinose (AraN4) modification of pathogenic bacteria lipid A has been related to resistance to polymyxin B [32, 33]. *B*. *pseudomallei* has been reported to be resistant to polymyxin B [34]. Our lipid A structural characterizations showed that *B*. *pseudomallei* lipid A species were modified with Ara4N residues at the terminal phosphate groups that was observed by the ion at m/z 1801, and other associated ions. We postulated that the varying ion intensity of lipid A modified with Ara4N may contribute to variable polymyxin B resistance. To test the susceptibility of *B*. *pseudomallei* to polymyxin B, 21 representative isolates were chosen with notable varying ion intensity at m/z 1801. The results demonstrated all 21 isolates were resistant to polymyxin B with a minimum inhibitory concentrations (MICs) for all isolates ≥ 512 μg/ml.

### Activation of human TLR4 by *B*. *pseudomallei* isolates

**Fig 5:**
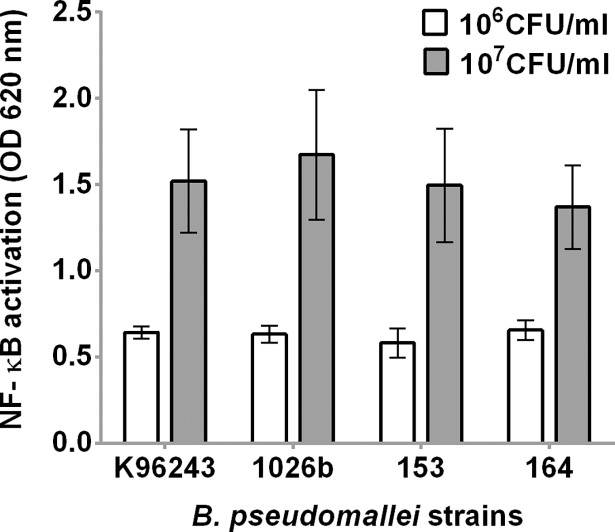
Activation of NF-κB in HEK-Blue hTLR4 cells by four isolates of *B*. *pseudomallei*. HEK-Blue hTLR4 cells were stimulated with heat-killed *B*. *pseudomallei* at 10^6^ or 10^7^ CFU/ml at 37°C in 5% CO_2_ for 24 h. NF-κB activation was determined in the supernatant by a SEAP reporter assay. Mean ± SD of three independent experiments were illustrated.

Our previous studies demonstrated that LPS of *B*. *pseudomallei* activates the innate immune responses in human monocytes through TLR4 [6, 35]. In this study, we considered whether different *B*. *pseudomallei* isolates cultured on the same medium differentially activate TLR4-dependent responses. We prepared heat-killed bacteria from four isolates (K96243, 1026b, 153 and 164) grown on TSA and stimulated HEK-Blue hTLR4 cells, expressing hTLR4-MD2-CD14 (Fig 5). All heat-killed *B*. *pseudomallei* isolates at 10^6^ CFU/ml were able to induce NF-κB activation after 24 h at OD levels between 0.49–0.73 (median = 0.63, IQR = 0.58–0.68). At bacterial concentrations of 10^7^ CFU/ml, the NF-κB activation was induced at OD level between 1.02 and 2.17 (median = 1.48, IQR = 1.24–1.72), respectively. We demonstrated no significant difference in TLR4-dependent NF-κB activation in cells stimulated with the four heat-killed bacterial isolates at 10^6^ CFU/ml (*P* = 0.12) or 10^7^ CFU/ml (*P* = 0.38).

**Fig 6:**
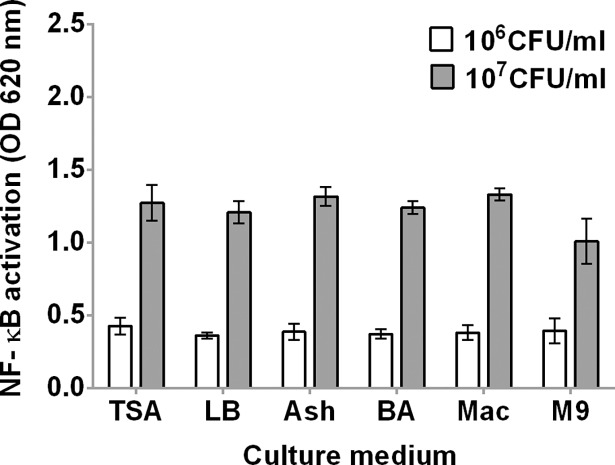
Activation of NF-κB in HEK-Blue hTLR4 cells by *B*. *pseudomallei* K96243 cultured in different media. HEK-Blue hTLR4 cells were stimulated with heat-killed *B*. *pseudomallei* K96243 at 10^6^ or 10^7^ CFU/ml at 37°C in 5% CO_2_ for 24 h. NF-κB activation was determined in the supernatant by a SEAP reporter assay with HEK-Blue detection. Mean ± SD of three independent experiment were illustrated. TSA; trypticase soy agar, LB; Luria-Bertani agar, Ash; Ashdown agar, BA; Blood agar, Mac; MacConkey agar, M9; M9 minimal medium agar.

To test whether *B*. *pseudomallei* isolates cultured on various media differentially activate TLR4-dependent responses, we prepared heat-killed *B*. *pseudomallei* K96243 grown on six different agar media (TSA, LB, Ashdown agar, blood agar, MacConkey agar, M9 minimal medium agar) and stimulated HEK-Blue hTLR4 cells with bacteria at 10^6^ and 10^7^ CFU/ml (Fig 6). We demonstrated no significant difference in NF-κB activation induced by heat-killed *B*. *pseudomallei* K96243 on different media at 10^6^ CFU/ml (*P* = 0.47). However, NF-κB activation induced by 10^7^ CFU/ml of heat-killed *B*. *pseudomallei* K96243 cultured on M9 minimal medium agar was significantly lower than that induced by K96243 cultured on other media (*P* < 0.0001).

### Activation of human TLR4 by LPS of *B*. *pseudomallei* isolates

**Fig 7:**
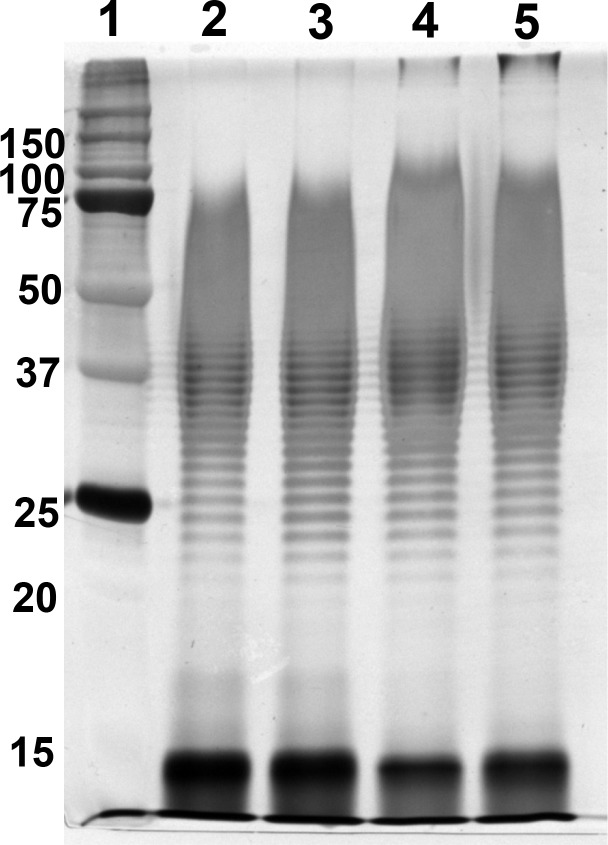
Purified LPS from *B*. *pseudomallei* isolates K96243, 1026b, 153 and 164. LPS was purified by a modified hot phenol-water extraction method. Five micrograms of LPS was run by SDS-PAGE electrophoresis followed by silver staining. Lane 1 was a protein marker (number at the left are masses in kilodaltons). Lanes 2–5 were the purified LPS of *B*. *pseudomallei* strains K96243, 1026b, 153 and 164, respectively.

**Fig 8:**
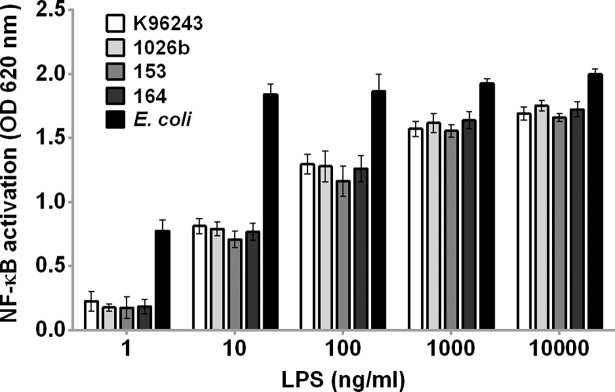
Activation of NF-κB in HEK-Blue hTLR4 cells by LPS of four *B*. *pseudomallei* clinical isolates. The HEK-Blue hTLR4 cells were stimulated with LPS from *B*. *pseudomallei* K96243, 1026b, 153 and 164 at 1, 10, 100, 1000, and 10,000 ng/ml at 37°C in 5% CO_2_ for 24 h. The positive control was ultrapure *Escherichia coli* O111:B4 LPS. NF-κB activation was determined in the supernatant using a SEAP reporter assay. Mean ± SD of two independent experiments were illustrated.

We previously demonstrated that LPS is a main driver of the innate immune response to *B*. *pseudomallei* [6]. Therefore, we further investigated whether LPS from different isolates potentially induce variable NF-κB activation. SDS-PAGE and silver stain of purified LPSs from all four isolates showed typical patterns of LPS type A (Fig 7). The Coomassie blue staining and BCA assay results showed no protein contamination. We determined NF-κB activation of HEK-Blue hTLR4 cells at 24 h after stimulation with LPSs from four *B*. *pseudomallei* isolates (K96243, 1026b, 153 and 164) at concentrations of 1, 10, 100, 1000, and 10,000 ng/ml. The LPSs of all isolates induced a significant increase of NF-κB activation in HEK-Blue hTLR4 cells above the baseline level in dose-dependent manner (Fig 8). Our results showed no significant difference among different *B*. *pseudomallei* isolates in TLR4-dependent NF-κB activation in cells stimulated with LPSs at 1 ng/ml (*P* = 0.68), 10 ng/ml (*P* = 0.14), 100 ng/ml (*P* = 0.33), 1000 ng/ml (*P* = 0.28) or 10,000 ng/ml (*P* = 0.29).

### Activation of human TLR4 by heat-killed *B*. *pseudomallei* OPS and capsule mutants

**Fig 9:**
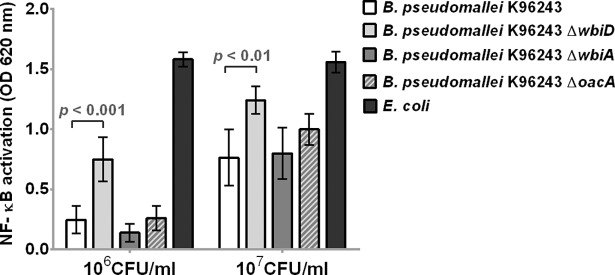
Activation of NF-κB in HEK-Blue hTLR4 cells by heat-killed wild type K96243 and mutant containing unmarked deletions of *wbiD*, *wbiA*, *and oacA*. HEK-Blue hTLR4 cells were stimulated with heat-killed bacteria at 10^6^ or 10^7^ CFU/ml at 37°C in 5% CO_2_ for 24 h. The positive control was *E*. *coli* ATCC 25922. NF-κB activation was determined in the supernatant by a SEAP reporter assay. Mean ± SD of two independent experiments were illustrated.

Although lipid A is the ligand for TLR4 [35], we hypothesized that there may be an effect of the OPS component of the LPS on this interaction. To investigate whether the presence of OPS on *B*. *pseudomallei* cells was associated with altered TLR4 signaling, we stimulated HEK-Blue^TM^-hTLR4 cells with heat-killed wild type K96243 and OPS mutant strains containing unmarked deletions of *wbiD*, *wbiA*, and *oacA*, which are involved in *B*. *pseudomallei* OPS biosynthesis [30]. We have previously shown that–like wild type *B. pseudomallei* K96243 –K96243 Δ*wbiA* and Δ*oacA* have type A ladder patterns, whereas K96243 Δ*wbiD* completely lacks OPS [30]. As shown in Fig 9, NF-κB activation was significantly higher in cells stimulated with 10^6^ (*P* < 0.001) and 10^7^ (*P* < 0.01) CFU/ml of *B*. *pseudomallei* K96243 Δ*wbiD* compared to those of wild type. We demonstrated no significant difference in TLR4-dependent NF-κB activation in cells stimulated with heat-killed bacteria obtained from *B*. *pseudomallei* K96243 Δ*wbiA* and Δ*oacA* compared to wild type at 10^6^ CFU/ml or 10^7^ CFU/ml. These data show that the absence of OPS does permit enhanced TLR4 signaling by heat killed *B*. *pseudomallei*.

**Fig 10:**
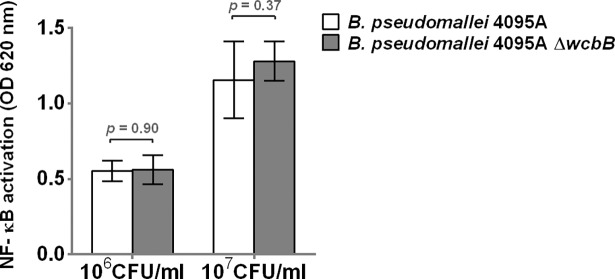
Activation of NF-κB in HEK-Blue hTLR4 cells by heat-killed wild type 4095A and mutant containing unmarked deletions of *wcbB*. The HEK-Blue hTLR4 cells were stimulated with heat-killed bacteria at 10^6^ or 10^7^ CFU/ml at 37°C in 5% CO_2_ for 24 h. NF-κB activation was determined in the supernatant by a SEAP reporter assay. Mean ± SD of two independent experiments were illustrated.

To further test whether the presence of capsule of *B*. *pseudomallei* cells interfered with TLR4 signaling, we stimulated HEK-Blue^TM^-hTLR4 cells with heat-killed wild type strain 4095a and a mutant containing unmarked deletions of *wcbB* (4095a Δ*wcbB*), which is involved in *B*. *pseudomallei* capsule biosynthesis [30]. Confirmatory testing with latex agglutination, SDS-PAGE, and Western blot of heat-killed bacteria indicated that the 4095a Δ*wcbB* did not express capsule. The results in Fig 10 demonstrated that NF-κB activation was not different in cells stimulated with 10^6^ (*P* = 0.90) and 10^7^ (*P* = 0.37) CFU/ml of *B*. *pseudomallei* 4095a Δ*wcbB*, as compared to those of wild type. Thus, the deletion of the capsule gene does not affect TLR4 signaling by heat killed *B*. *pseudomallei*.

### Activation of human TLR4 by LPS of *B*. *pseudomallei* OPS mutant

**Fig 11:**
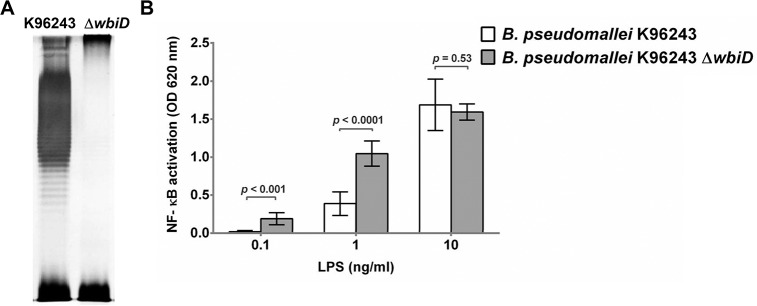
Purified LPS from *B*. *pseudomallei* strain K96243 and K96243 Δ*wbiD*. LPS was purified by a modified hot phenol-water extraction method. Five micrograms of LPS was run by SDS-PAGE electrophoresis followed by silver staining (A). Activation of NF-κB in HEK-Blue hTLR4 cells by LPS at 0.1, 1 and 10 ng/ml of *B*. *pseudomallei* K96243 and K96243Δ*wbiD* (B). NF-κB activation was determined in the supernatant using a SEAP reporter assay. The data were calculated to account for the difference in weight of wild type LPS and Δ*wbiD* mutant LPS. Mean ± SD of two independent experiments were illustrated.

We then tested the impact of the OPS mutant on TLR4-dependent NF-κB activation by *B*. *pseudomallei* LPS. LPS was extracted and verified by SDS-PAGE and silver staining confirming the structural difference in LPS between wild type and isogenic mutant. The results in Fig 11A revealed ladder pattern with the presence of OPS for K96243 wild type but not for the K96243 Δ*wbiD* mutant, indicating the lack of OPS biosynthesis [30]. Stimulation of HEK-Blue^TM^-hTLR4 cells with different concentrations of LPSs from wild type and Δ*wbiD* mutant demonstrated that NF-κB activation was significantly higher in cells stimulated with Δ*wbiD* LPS at 0.1 ng/ml (*P* < 0.001) and 1 ng/ml, (*P* < 0.0001) compared with NF-κB activation in cells stimulated with wild type LPS. We observed no difference in NF-kB activation between cells stimulated with 10 ng/ml LPS of wild type and cells stimulated with 10 ng/ml LPS of the Δ*wbiD* mutant. We noted that the lipid A structure of K96243 Δ*wbiD* mutant and the wild type were identical (S6 Fig). These data provide additional confirmation that the absence of OPS allows enhanced TLR4 signaling by *B*. *pseudomallei* LPS.

## Discussion

Human melioidosis is characterized by hyperinflammatory responses leading to high mortality rates even in patients who have received appropriate antimicrobial therapy. The initial interaction between *B*. *pseudomallei* and innate immune receptors such as the LPS-TLR4 interaction is important in the immunopathogenesis and outcome of infection. In this study, we characterized lipid A structure of *B*. *pseudomallei* isolates by MALDI-TOF MS followed by ESI-QqTOF MS and GC and examined the innate immune responses to the different bacterial isolates. Our data revealed the same lipid A profile for *B*. *pseudomallei* from a large collection of diverse isolates of LPS type A and demonstrated minimal variability in TLR4 signaling. Despite being cultured in different laboratory-induced conditions, *B*. *pseudomallei* K96243 lipid A expressed similar lipid A profiles, as characterized by MALDI-TOF MS. Moreover, we demonstrated that *B*. *pseudomallei* OPS interfered with TLR4 activation.

Our data show that the major lipid A species of *B*. *pseudomallei* contain a mixture of tetra- and penta-acylated lipid A species that are non-stoichiometrically substituted with Ara4N residues at both phosphate groups. We observed the presence of fatty acid C14:0, C14:0(2-OH), C14:0(3-OH), C16:0, and C16:0(3-OH). In comparison with other bacterial species, the lipid A structure of *B*. *pseudomallei* is unique. Previous published data showed that the fatty acid C14:0(2-OH) substituted into lipid A backbone of *B*. *pseudomallei* was not present in other closely related *Burkholderia* species, such as *B*. *thailandensis*, *B*. *cepacia* and *B*. *mallei* [17, 36, 37].

Although genomic analysis of within-host isolates showed substantial divergence from the founder genotype during a short period of acute infection [22], we found a remarkable lack of lipid A structural variation in 35 clinical *B*. *pseudomallei* isolates cultured from multiple body sites from a single patient with disseminated melioidosis. The data suggest that the lipid A is essential for *B*. *pseudomallei* and the modification during short period of acute infection is not required for bacterial fitness. Lipid A has an important role as it is the anchor for LPS on the outer membrane of *B*. *pseudomallei*. We previously showed that the synthesis of lipid A molecules is of vital importance among the various components that are responsible for outer membrane assembly [38].

Surprisingly, our study revealed similar lipid A spectra of 136 clinical isolates obtained from primary and relapse infections of 68 melioidosis patients, thus confirming that all *B*. *pseudomallei* isolates in our collection expressed a similar lipid A structure. In comparison to *B*. *pseudomallei*, *B*. *mallei* also produce both tetra- and penta-acylated lipid A species that are potent stimulators of hTLR-4-dependent cytokine production [36]. Other studies demonstrated that *B*. *cenocepacia* strain LMG 12614 expressed only penta-acylated lipid A species and it can induce stronger TNF-α and IL-6 responses than *B*. *multivorans* strain LMG 14273 that expressed both tetra- and penta-acylated lipid A [39].

Gram-negative organisms have evolved several LPS modification that benefit these organisms in their interactions with the host innate immune system and hostile environments during infections. The alteration of lipid A structure can promote resistance to antimicrobial peptides and interfere with host recognition by the innate immune system. Alterations are accomplished by many enzymes that modify the lipid A moieties by adding or removing acyl chains and phosphate groups. In some bacteria, lipid A are modified to evade immune recognition and survive within a host by reducing the charge of the bacterial surface via adding sugar onto lipid A molecules. The addition of phosphoethanolamine and aminoarabinose to lipid A molecules can protect bacteria against cationic antimicrobial peptides (CAMPs) [40, 41]. In *Salmonella* serovar Typhimurium, aminoarabinose modification at both phosphate groups have increased resistance to cationic antimicrobial peptides [11]. Our characterization of lipid A revealed that the lipid A species of all *B*. *pseudomallei* isolates were modified with aminoarabinose residues, thus demonstrated the resistance to polymyxin B with MIC value >512 μg/ml.

We observed no alteration of lipid A structure when *B*. *pseudomallei* strain K96243 was cultured under standard laboratory-induced conditions. However, we observed a slight decreased TLR4-dependent NF-κB activation after culture using M9 media. A previous study demonstrated that a variety of growth conditions alter the expression of *Escherichia coli* LPS polysaccharide formation especially in nutrient-depleted conditions [42]. The changes in LPS expression may extend to lipid A synthesis and result less TLR4-dependent NF-κB activation. In contrast, various other Gram-negative bacteria modify their lipid A structure when exposed to the external environment *in vitro*. For example, temperature-dependent structural changes in lipid A structure have been shown in *Porphyromonas gingivalis* and *Yersinia* species. *P*. *gingivalis* expresses mainly nonphosphorylated and monophosphorylated tetra-acylated lipid A structure at normal body temperature, whereas the major lipid A produced are monophosphorylated penta-acylated lipid A when the bacteria are grown at 41 ^o^C. The temperature-dependent alteration in *P*. *gingivalis* lipid A structure is associated with more potent TLR4-dependent innate immune activation by LPS from bacteria grown at 41 ^o^C than LPS from bacteria cultured at 37 ^o^C [12]. Three pathogenic *Yersinia*; including *Y*. *pestis*, *Y*. *enterocolitica*, and *Y*. *pseudotuberculosis* synthesize predominantly hexa-acylated lipid A at 21 ^o^C which induces higher levels of cytokine production compared to tetra-acylated lipid A at 37 ^o^C [14]. Additionally, *Salmonella enterica* serovar Typhimurium undergoes hydroxylation of fatty acid C14:0 to fatty acid C14:0(2-OH) when grown under magnesium-depleted conditions [11].

We also observed that the heat-killed bacteria and LPSs from different *B*. *pseudomallei* isolates activated human TLR4 and we demonstrated no significant difference among different *B*. *pseudomallei* isolates in TLR4-dependent NF-κB activation. Similarly, LPS from four clinical isolates resulted in similar TLR4-dependent NF-κB activation. These results are concordant with the invariable MALDI-TOF results characterizing *B*. *pseudomallei* lipid A. While increased natural diversity or heterogeneity of specific components of LPS, such as lipid A, can produce dramatic changes in host responses, this is not the case for *B*. *pseudomallei*.

Our results differ from recent reports. Norris *et al*. described different lipid A profiles among each of different LPS types in *B*. *pseudomallei* and the effect on innate immune activation suggesting that clinical strains with different LPS types may be the cause of clinical outcomes. However, their study used only one strain of *B*. *pseudomallei* for each LPS type (type A, B, B_2_ and rough) [19]. Our study was conducted entirely using LPS type A, which is predominant in Thailand, yet despite analysis of LPS from over 170 clinical isolates, found no differences that could potentially explain clinical variations. Another reason for the discrepancy in results may be due to the different MALDI-TOF matrix used. We used norharmane as a matrix for MALDI-TOF in contrast to the 2,5-dihydroxybenzoic acid used by Norris and colleagues. Norharmane is an optimum matrix for overcoming limitations of lipid A detection [24].

Despite our previous identification of *B*. *pseudomallei* lipid A as a TLR4 agonist [35], we also show here that TLR4-dependent NF-κB activation induced by an OPS mutant (rough LPS) is significantly greater than those induced by LPS from wild type *B*. *pseudomallei*. However, the response induced by a *B*. *pseudomallei* capsule mutant was comparable to those induced by the wild type. This observation is concordant with findings from a previous study showing that rough *B*. *pseudomallei* LPS induces higher nitric oxide and TNF-α levels in RAW 264.7 macrophages than smooth LPS [19], and suggests that there may be interference by the OPS on the LBP/CD14-mediated presentation or binding of LPS to the TLR4/MD-2 complex [9].

In conclusion, we have demonstrated that the structural features of *B*. *pseudomallei* lipid A are extremely conserved among different clinical *B*. *pseudomallei* isolates with LPS type A and result in no variation in TLR4-dependent innate immune activation. Further studies are required to evaluate the lipid A structure of environmental isolates and the role of OPS in the LPS-TLR4 interaction.

## Supporting information

S1 Fig Tandem MS spectra of *B. pseudomallei* strain K96243 lipid A by ESI-QqTOF. (PDF)

S2 Fig MALDI-TOF lipid A spectra of clinical isolates of *B*. *pseudomallei* from 68 Thai patients with primary infection. (PDF)

S3 Fig MALDI-TOF lipid A spectra of clinical isolates of *B*. *pseudomallei* from 68 Thai patients with relapse infection. (PDF)

S4 Fig MALDI-TOF lipid A spectra of 35 individual colonies of *B*. *pseudomallei* from 7 clinical specimens (5 colonies for each specimen) from a Thai patient with acute melioidosis. (PDF)

S5 Fig MALDI-TOF lipid A spectra of clinical isolates of *B*. *pseudomallei* strain K96243 cultured in different laboratory conditions. (PDF)

S6 Fig MALDI-TOF lipid A spectra of *B*. *pseudomallei* K96243 and *B*. *pseudomallei* K96243 Δ*wbiD*. (PDF)

S1 Table *B*. *pseudomallei* wild type and mutant strains used in this study. (PDF)

S2 Table Clinical isolates of *B*. *pseudomallei* from 68 Thai patients with episode of relapse and date of isolation and specimen types. (PDF)

S3 Table Clinical isolates of *B*. *pseudomallei* from 35 individual colonies of 7 clinical specimens used in this study. (PDF)
