# Supplementary figures and images for: Comprehensive analysis of clinical Burkholderia pseudomallei isolates demonstrates conservation of unique lipid A structure and TLR4-dependent innate immune activation
Source: PLoS Negl Trop Dis. 2018 Feb 23;12(2):e0006287. doi: 10.1371/journal.pntd.0006287 (PMC5842036; doi:10.1371/journal.pntd.0006287)

S1 Fig. Tandem MS spectra by ESI-QqTOF

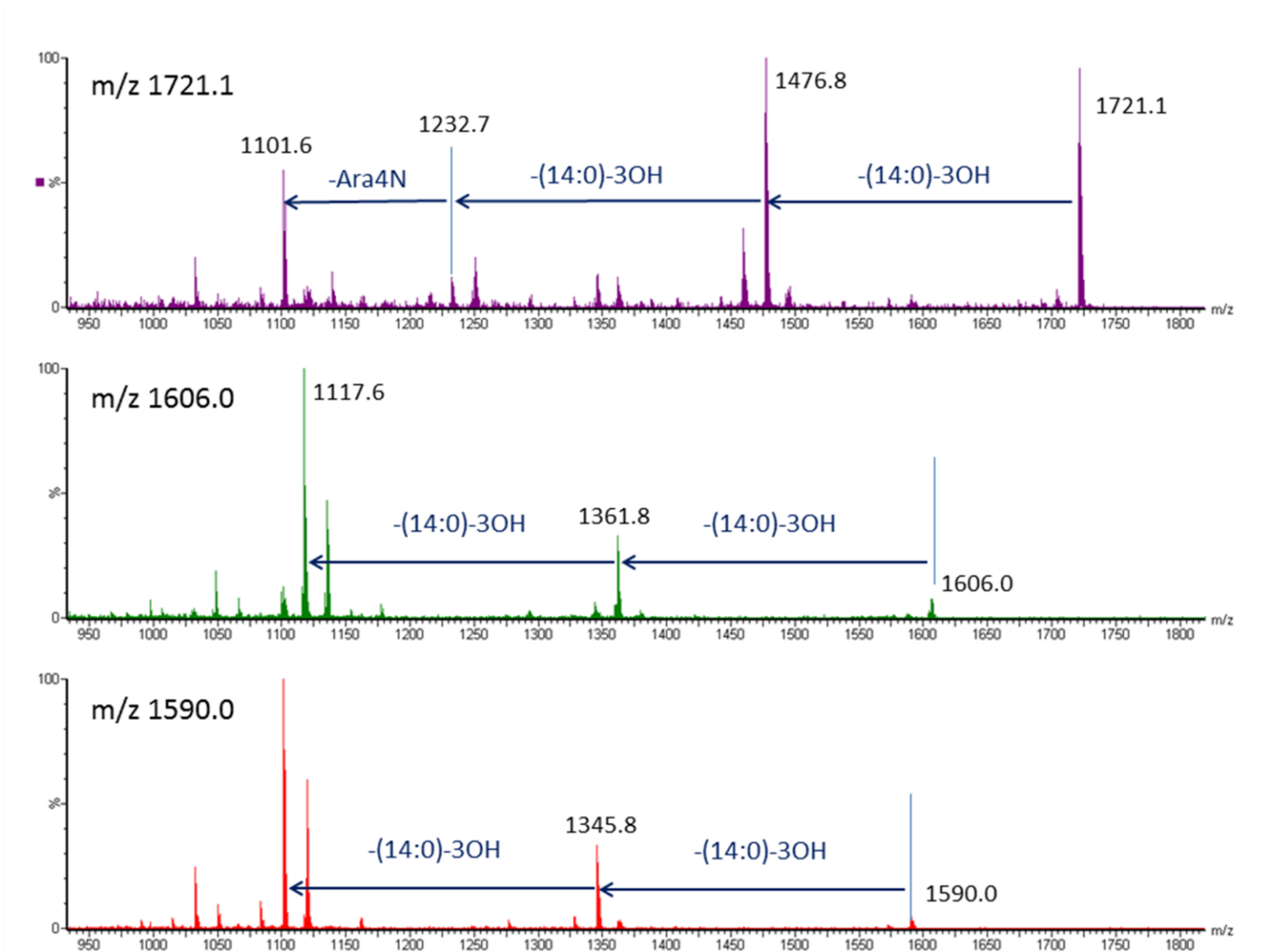

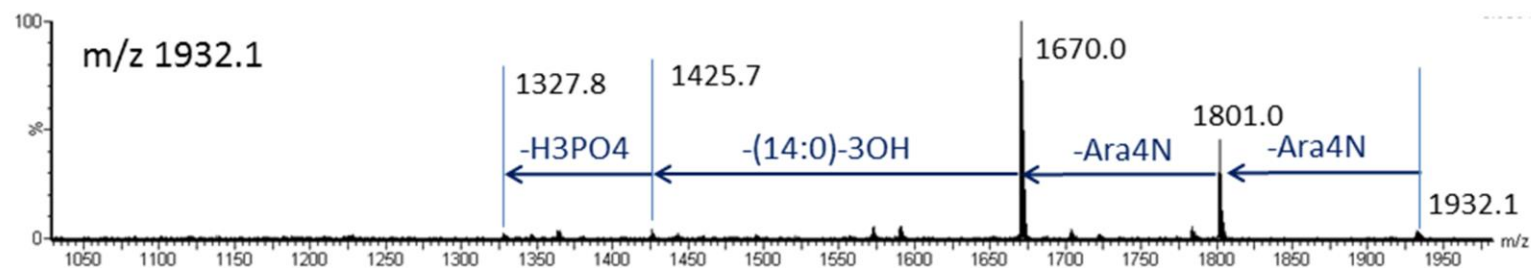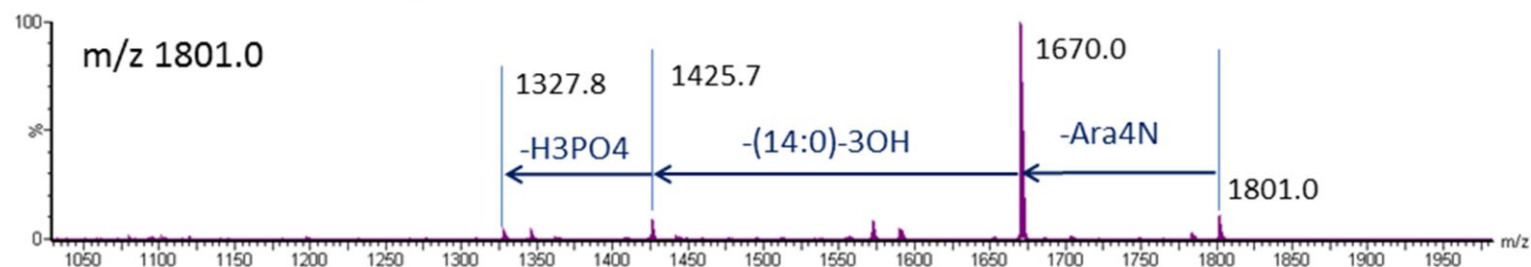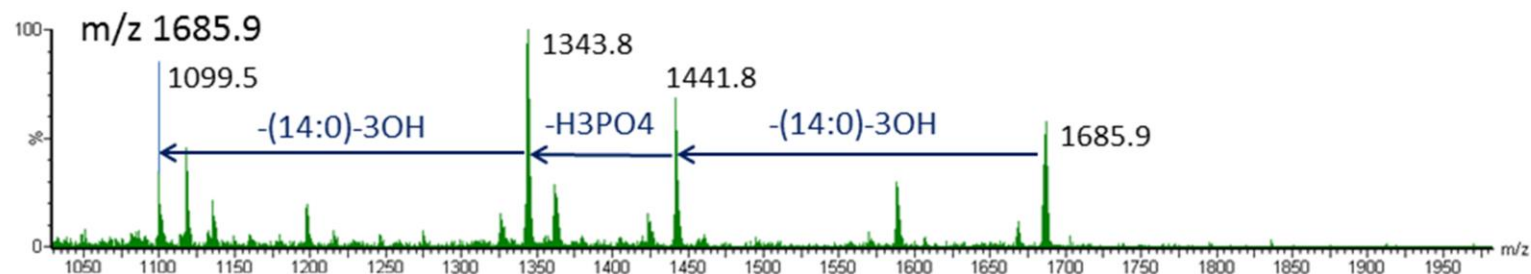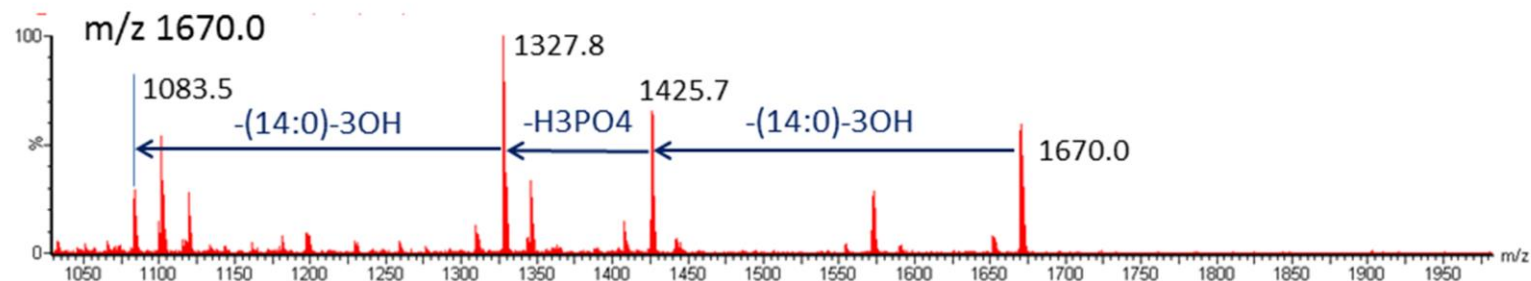

Supplement: S1 Fig — (PDF) [file pntd.0006287.s001.pdf]

S6 Fig. MALDI-TOF lipid A spectra of *B. pseudomallei* K96243 and *B. pseudomallei* K96243  $\Delta wbiD$

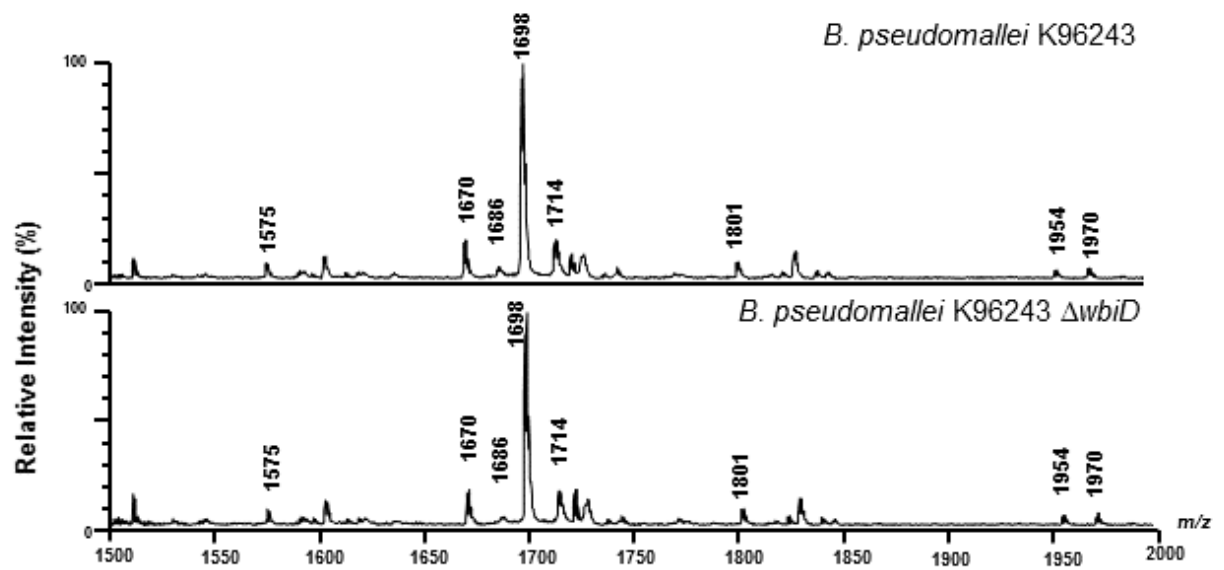

Supplement: S6 Fig — (PDF) [file pntd.0006287.s006.pdf]
