# Supplementary material for: Comprehensive analysis of clinical Burkholderia pseudomallei isolates demonstrates conservation of unique lipid A structure and TLR4-dependent innate immune activation
Source: PLoS Negl Trop Dis. 2018 Feb 23;12(2):e0006287. doi: 10.1371/journal.pntd.0006287 (PMC5842036; doi:10.1371/journal.pntd.0006287)
Supplement: S2 Fig — (PDF) [file pntd.0006287.s002.pdf]

S2 Fig. MALDI-TOF lipid A spectra of clinical isolates of *B. pseudomallei* from 68 Thai patients with primary infection

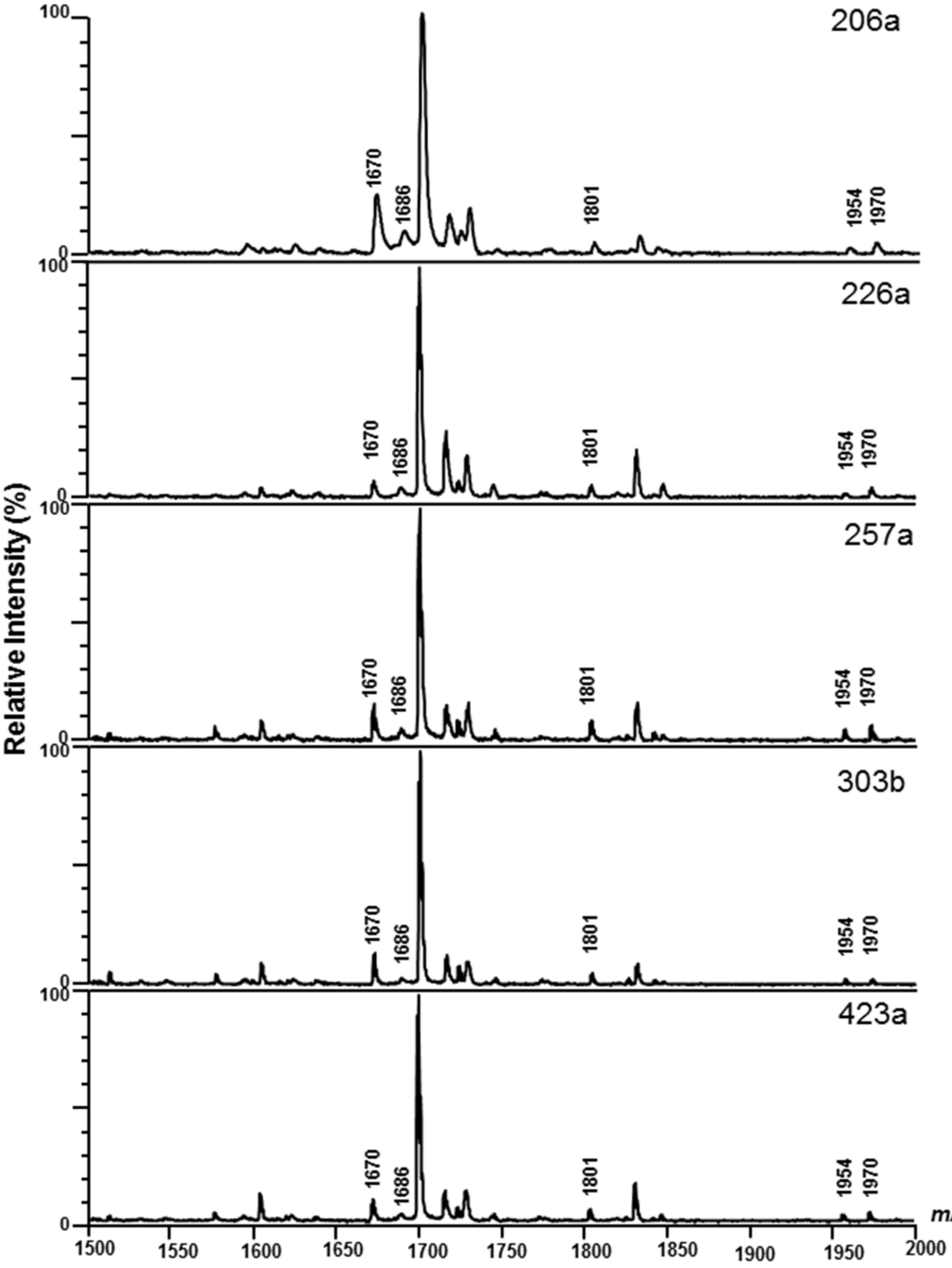

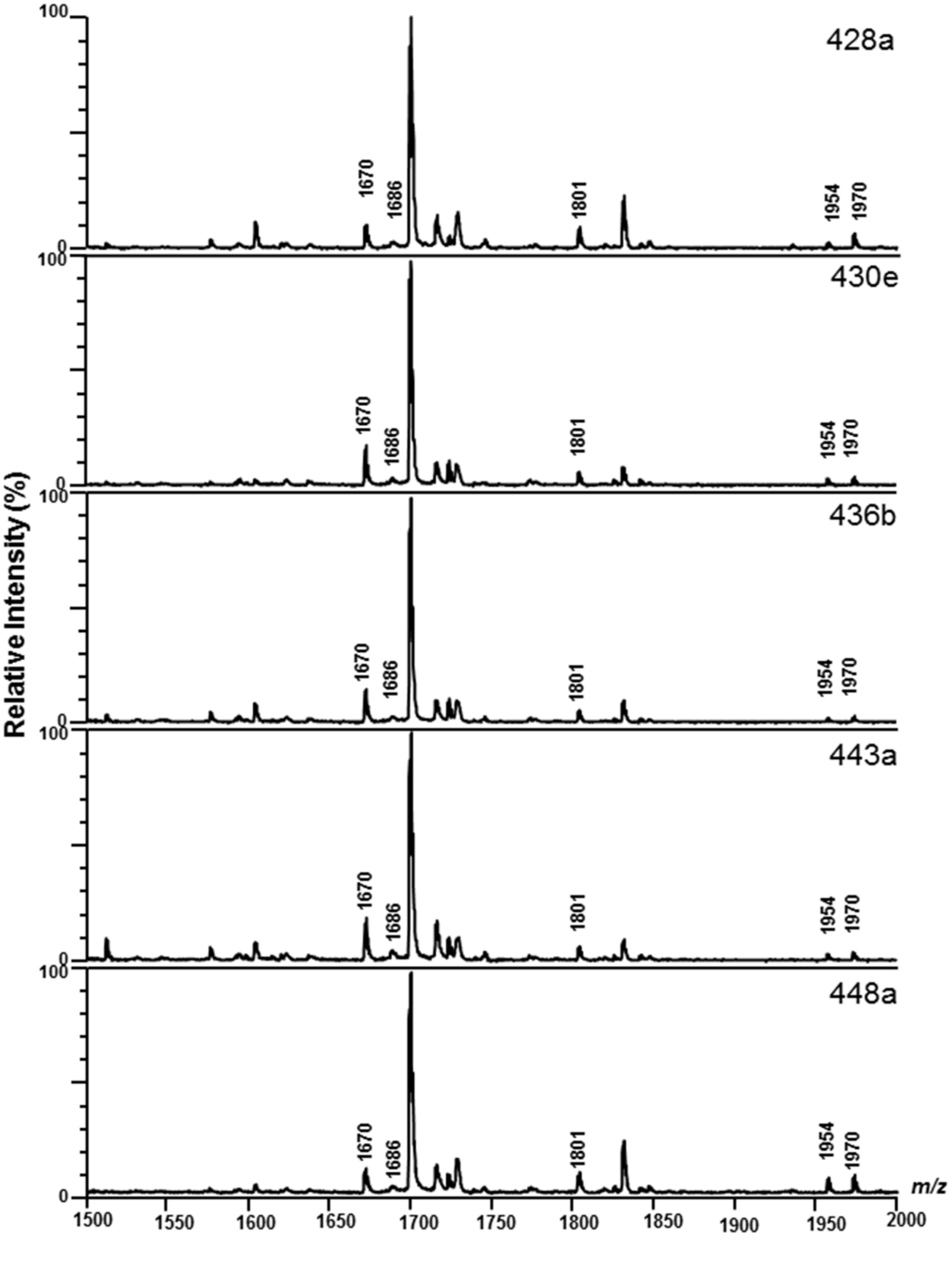

Relative Intensity (%)
100
0
428a
1670
1686
1801
1954
1970
430e
436b
443a
448a
m/z
1500
1550
1600
1650
1700
1750
1800
1850
1900
1950
2000

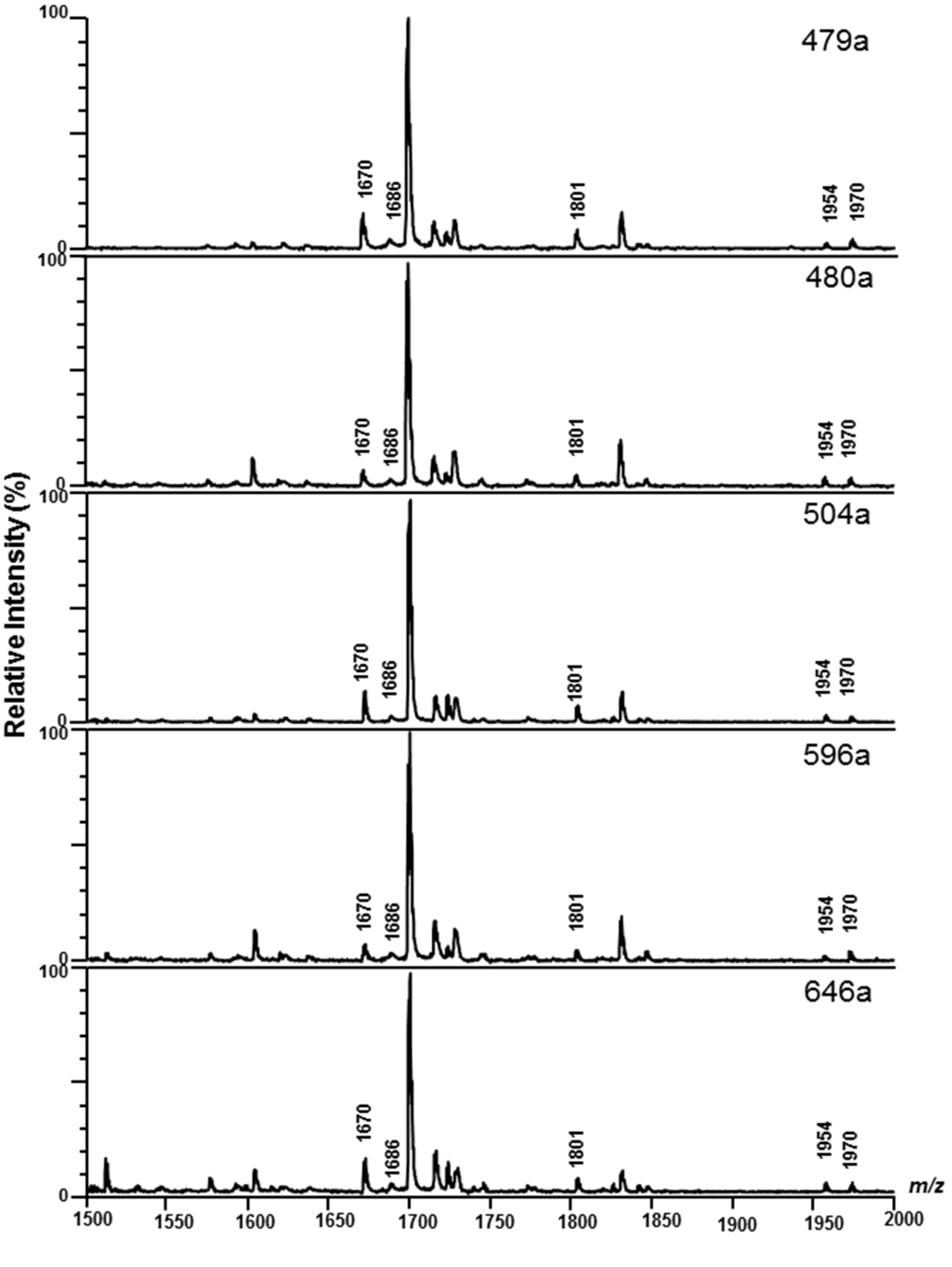

Relative Intensity (%)
479a
480a
504a
596a
646a
100
0
1670
1686
1801
1954
1970
m/z
1500
1550
1600
1650
1700
1750
1800
1850
1900
1950
2000

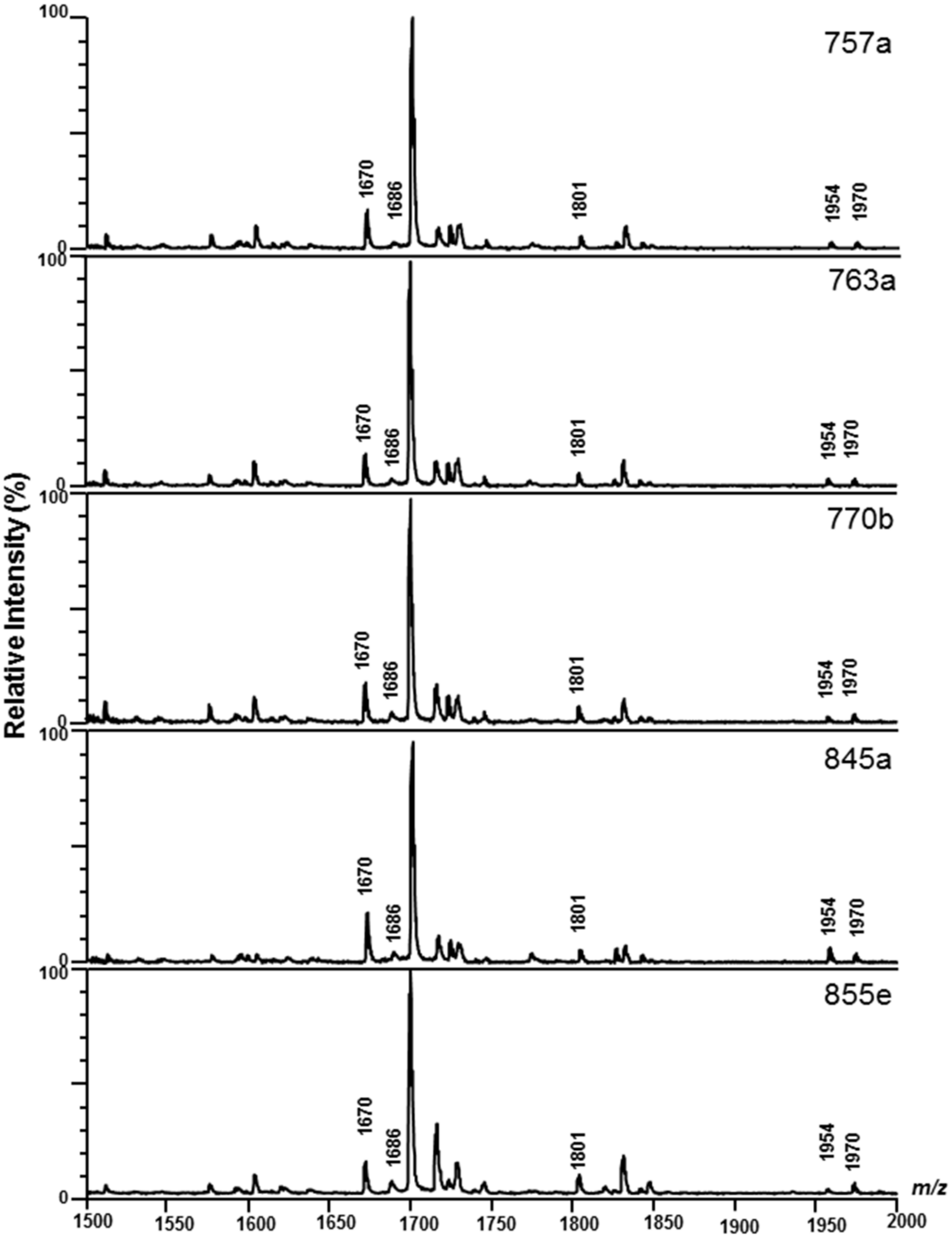
757a
763a
770b
845a
855e
1670
1686
1801
1954
1970
100
0
Relative Intensity (%)
m/z
1500
1550
1600
1650
1700
1750
1800
1850
1900
1950
2000

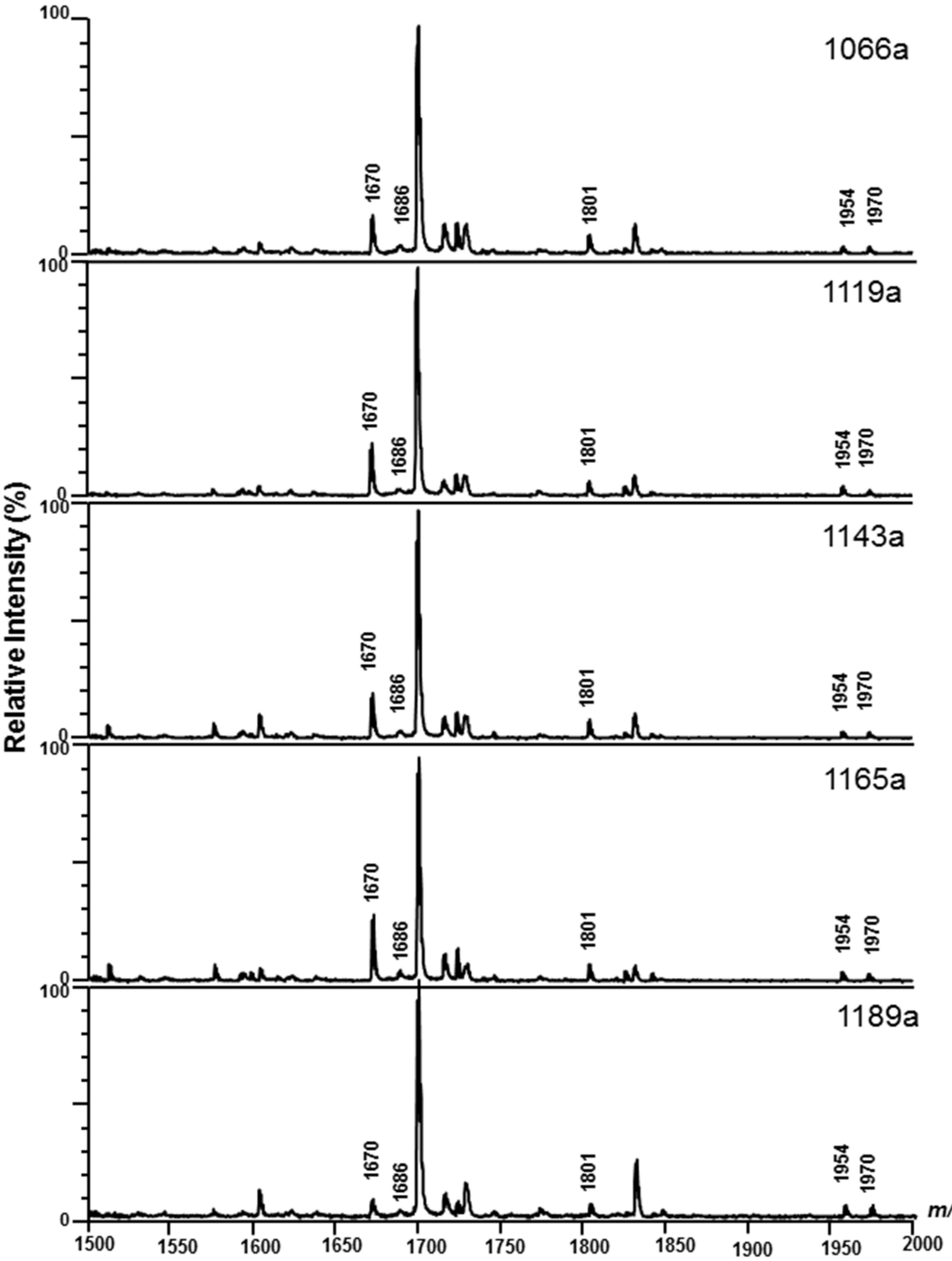

Relative Intensity (%)
100
0
1066a
1119a
1143a
1165a
1189a
1670
1686
1801
1954
1970
m/z
1500
1550
1600
1650
1700
1750
1800
1850
1900
1950
2000

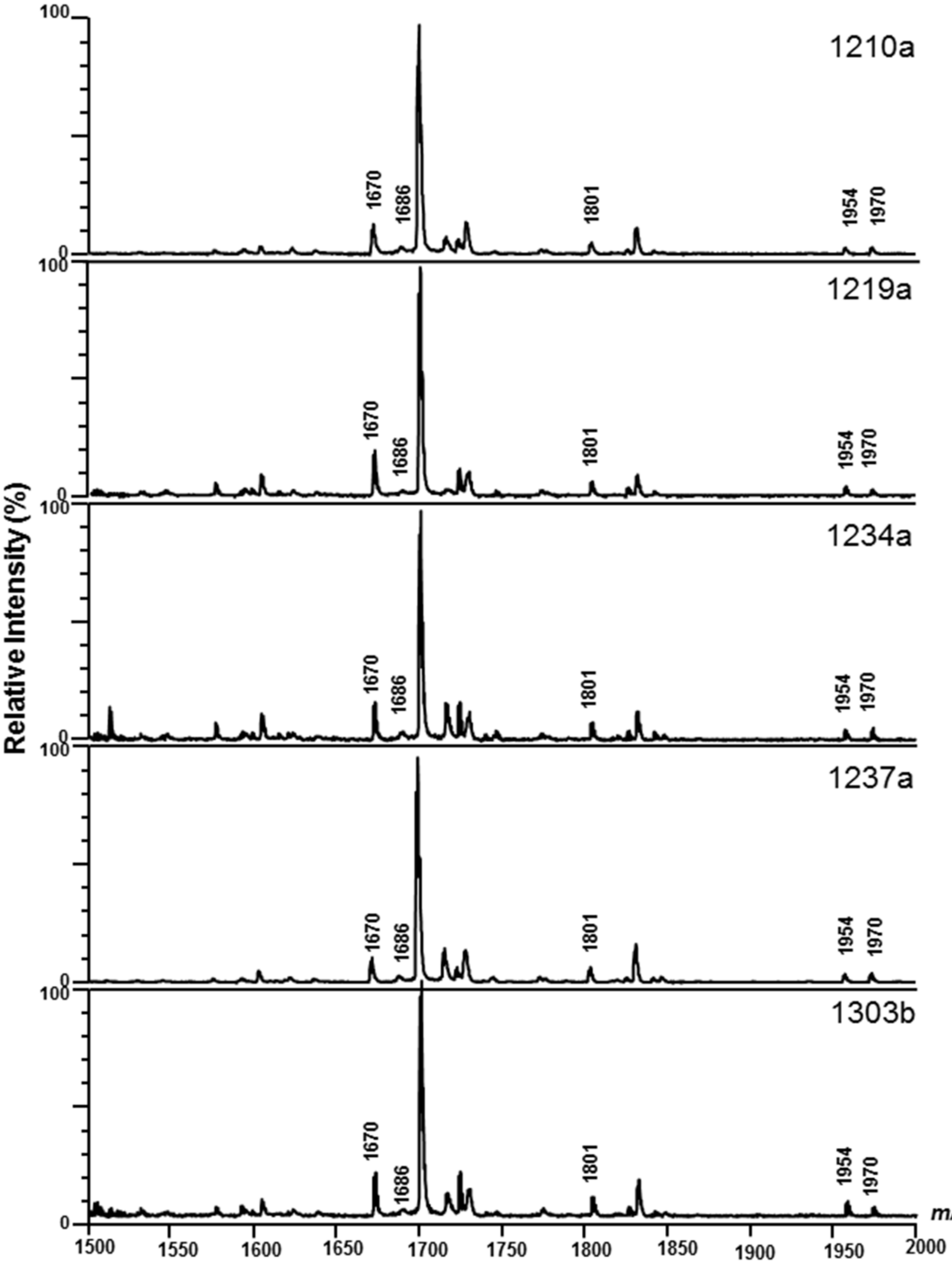

Relative Intensity (%)
100
0
1210a
1219a
1234a
1237a
1303b
1670
1686
1801
1954
1970
m/z
1500
1550
1600
1650
1700
1750
1800
1850
1900
1950
2000

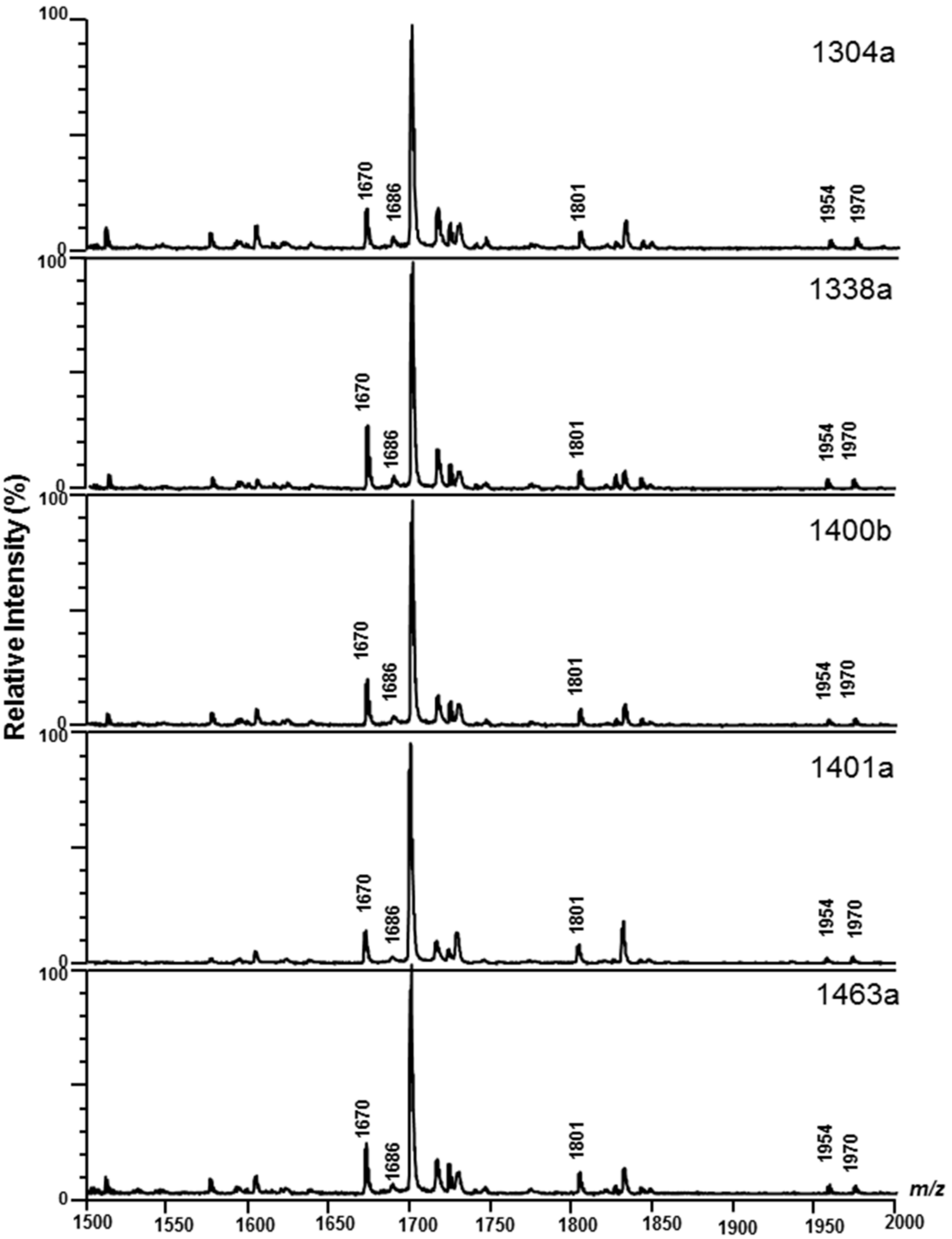

Relative Intensity (%)
100
0
1304a
1670
1686
1801
1954
1970
1338a
1400b
1401a
1463a
m/z
1500
1550
1600
1650
1700
1750
1800
1850
1900
1950
2000

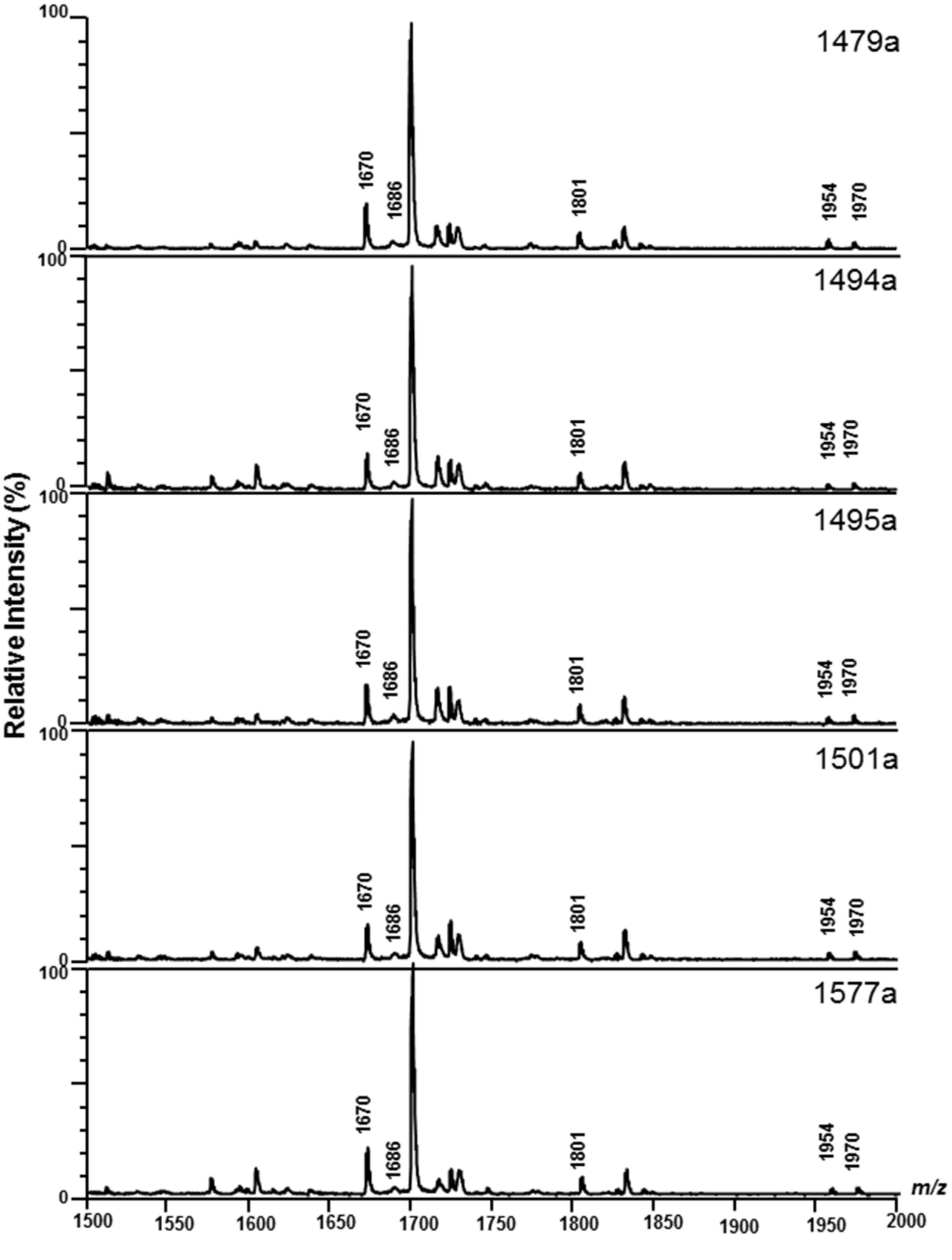

Relative Intensity (%)
1479a
1494a
1495a
1501a
1577a
1670
1686
1801
1954
1970
100
0
m/z
1500
1550
1600
1650
1700
1750
1800
1850
1900
1950
2000

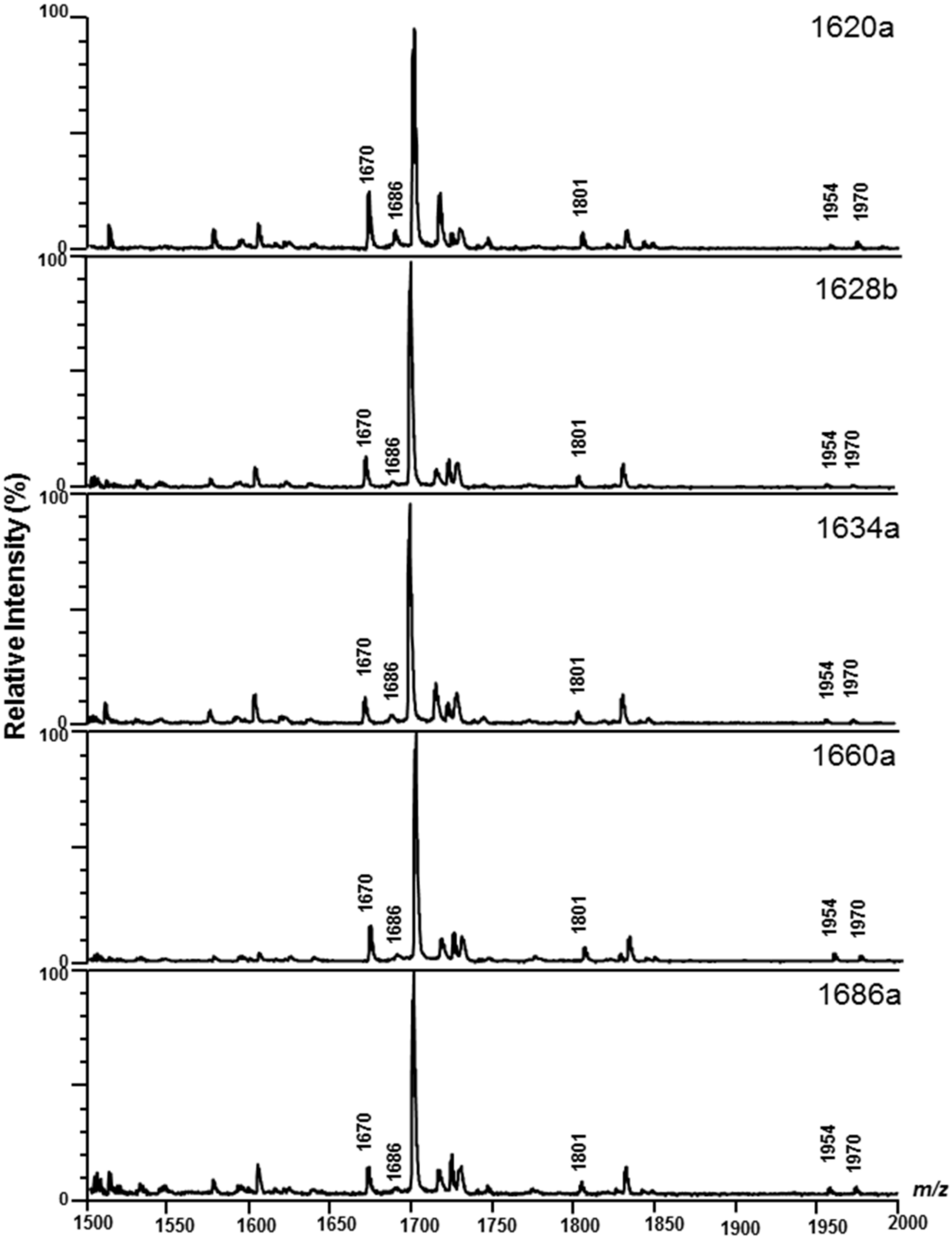

Relative Intensity (%)
1620a
1628b
1634a
1660a
1686a
100
0
1670
1686
1801
1954
1970
m/z
1500
1550
1600
1650
1700
1750
1800
1850
1900
1950
2000

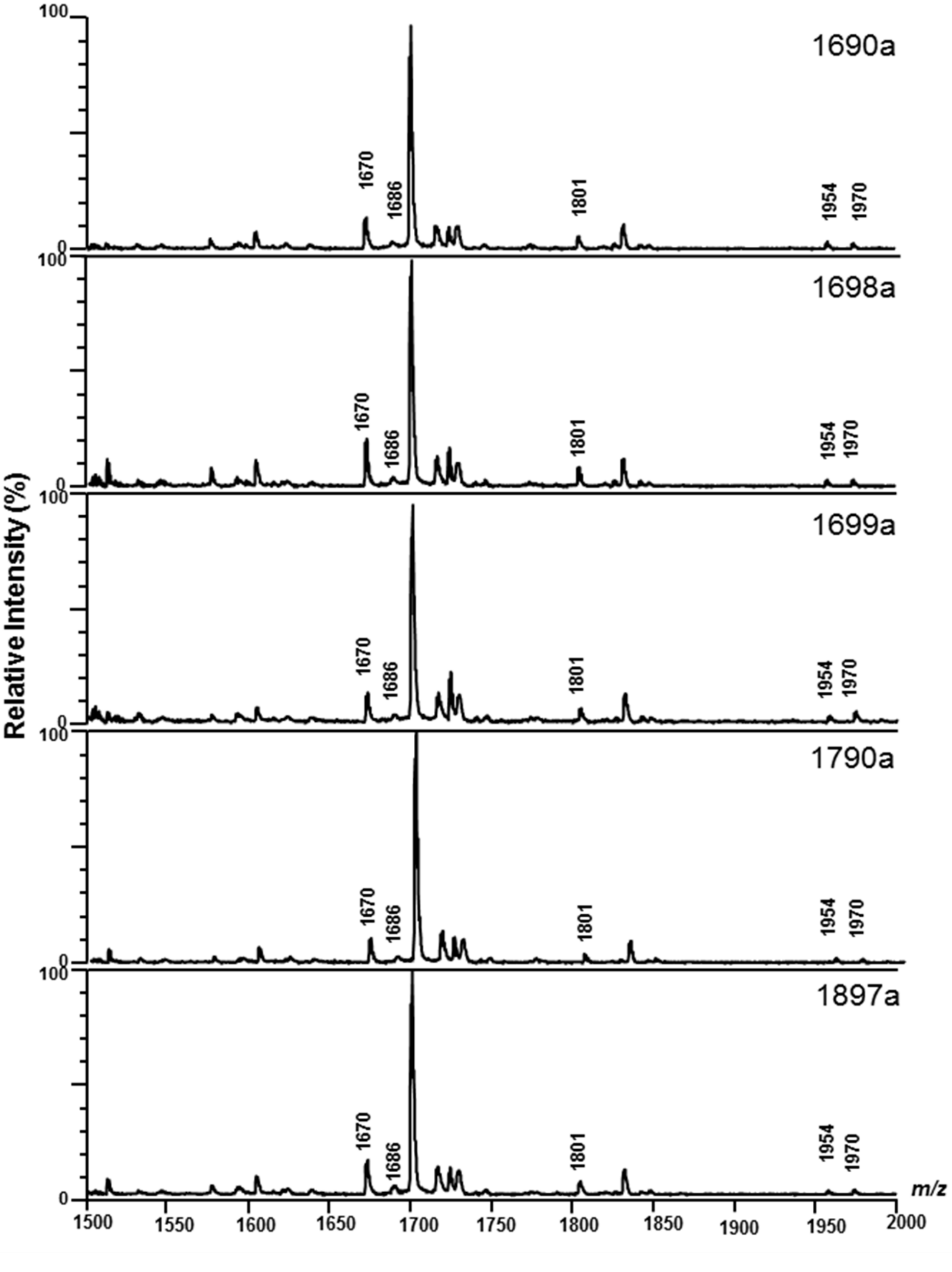

Relative Intensity (%)
100
0
1690a
1698a
1699a
1790a
1897a
1670
1686
1801
1954
1970
m/z
1500
1550
1600
1650
1700
1750
1800
1850
1900
1950
2000

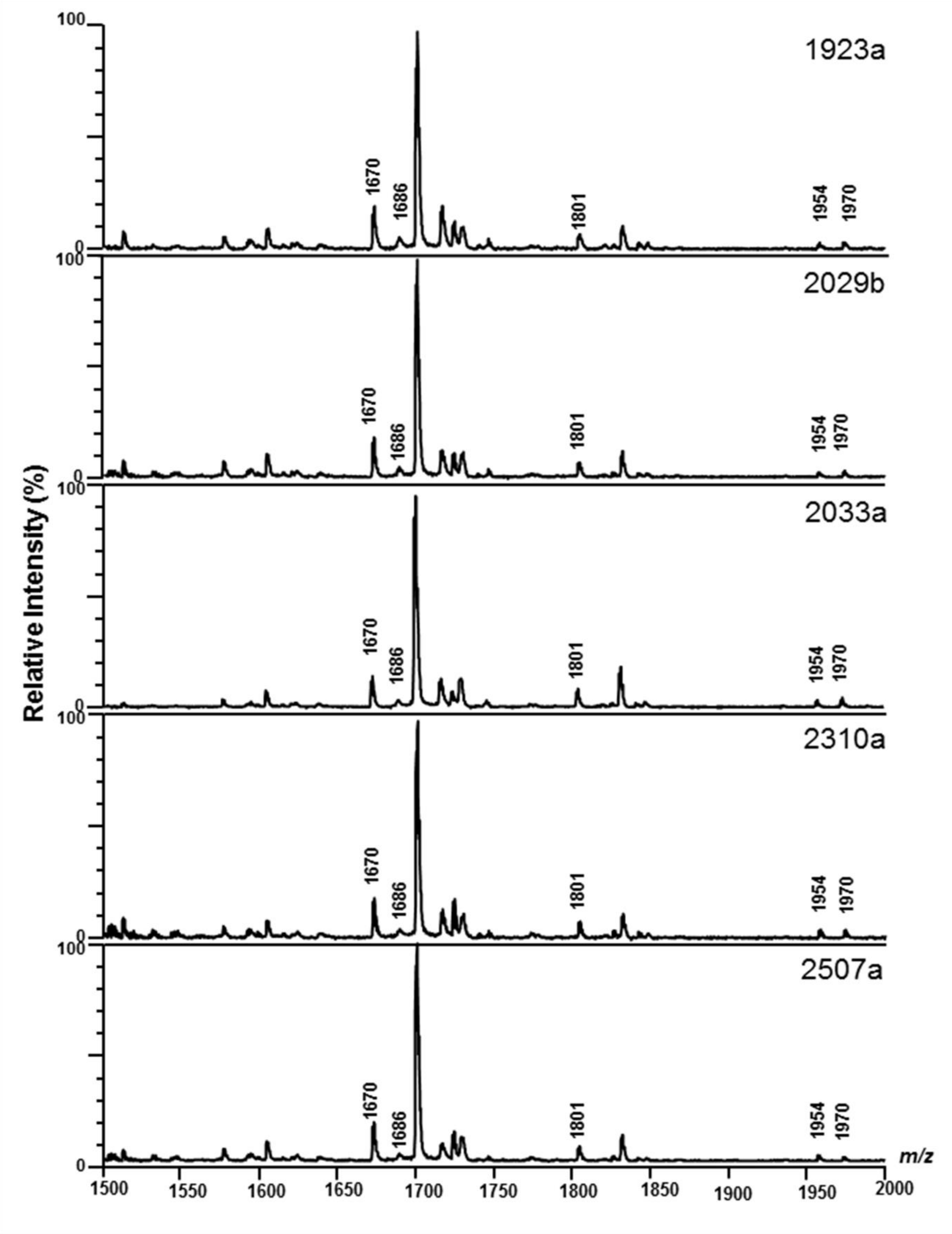

Relative Intensity (%)
100
0
1923a
2029b
2033a
2310a
2507a
1670
1686
1801
1954
1970
m/z
1500
1550
1600
1650
1700
1750
1800
1850
1900
1950
2000

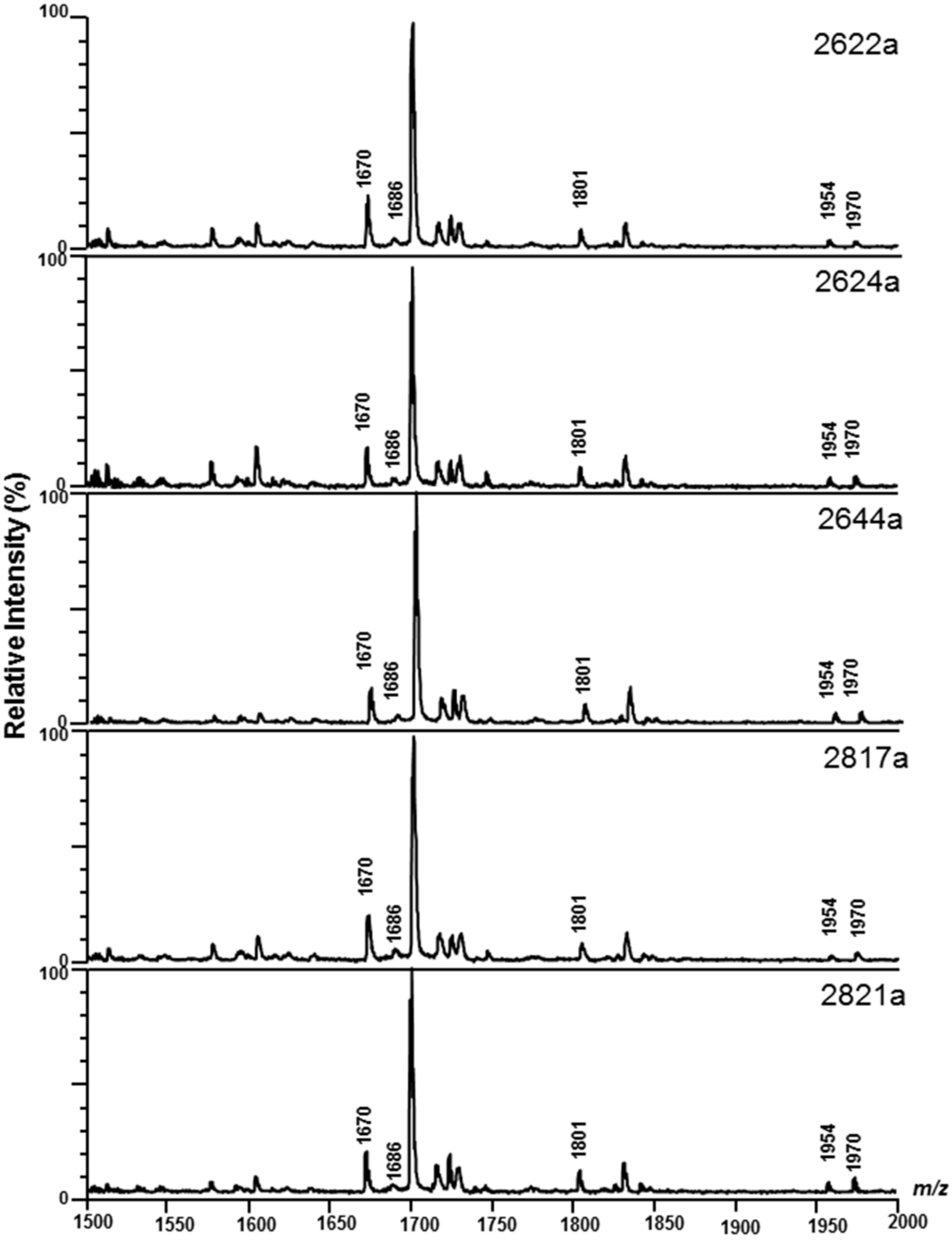

Relative Intensity (%)
2622a
2624a
2644a
2817a
2821a
100
0
1670
1686
1801
1954
1970
m/z
1500
1550
1600
1650
1700
1750
1800
1850
1900
1950
2000

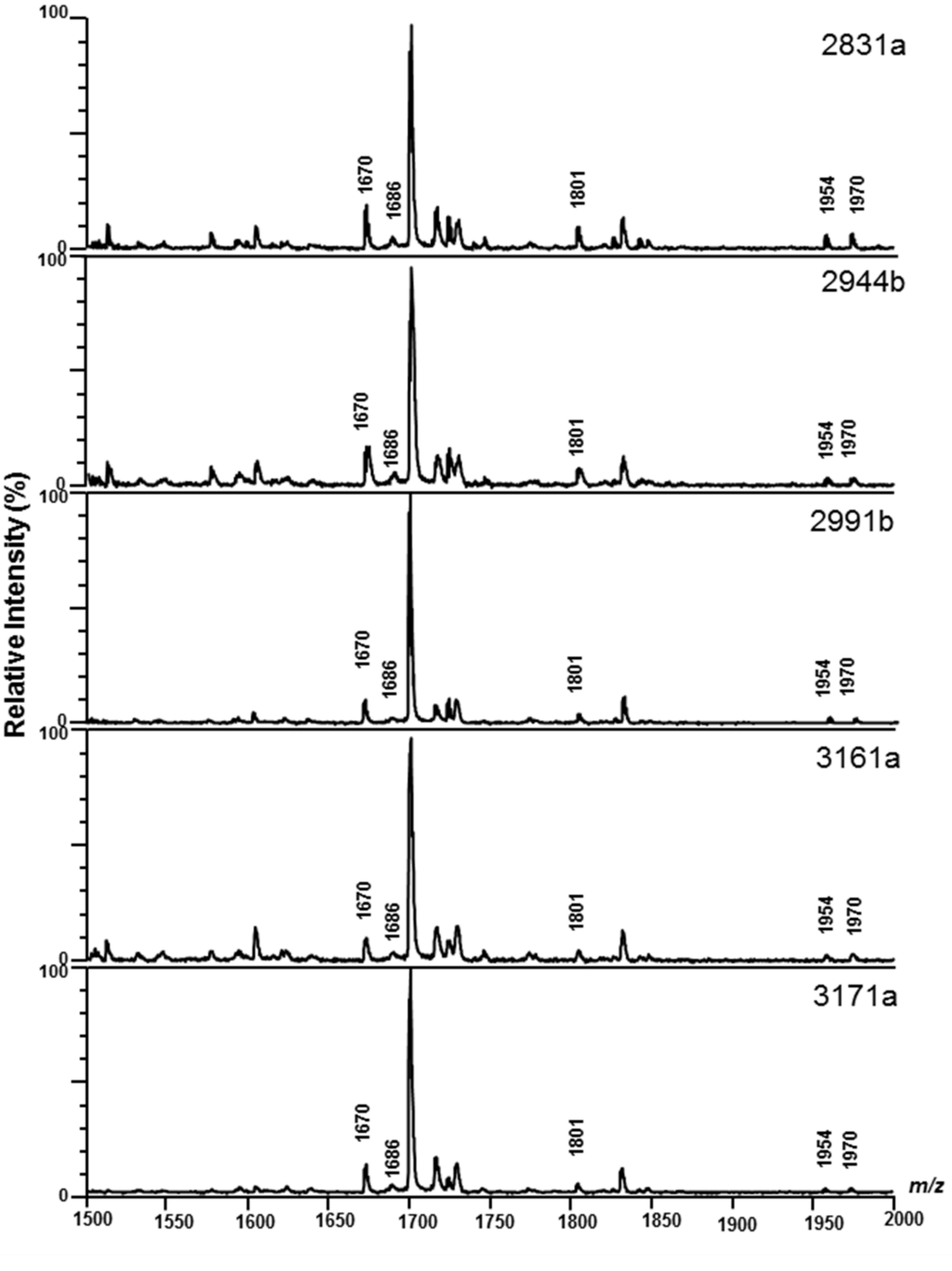

2831a
2944b
2991b
3161a
3171a
1670
1686
1801
1954
1970
Relative Intensity (%)
100
0
m/z
1500
1550
1600
1650
1700
1750
1800
1850
1900
1950
2000

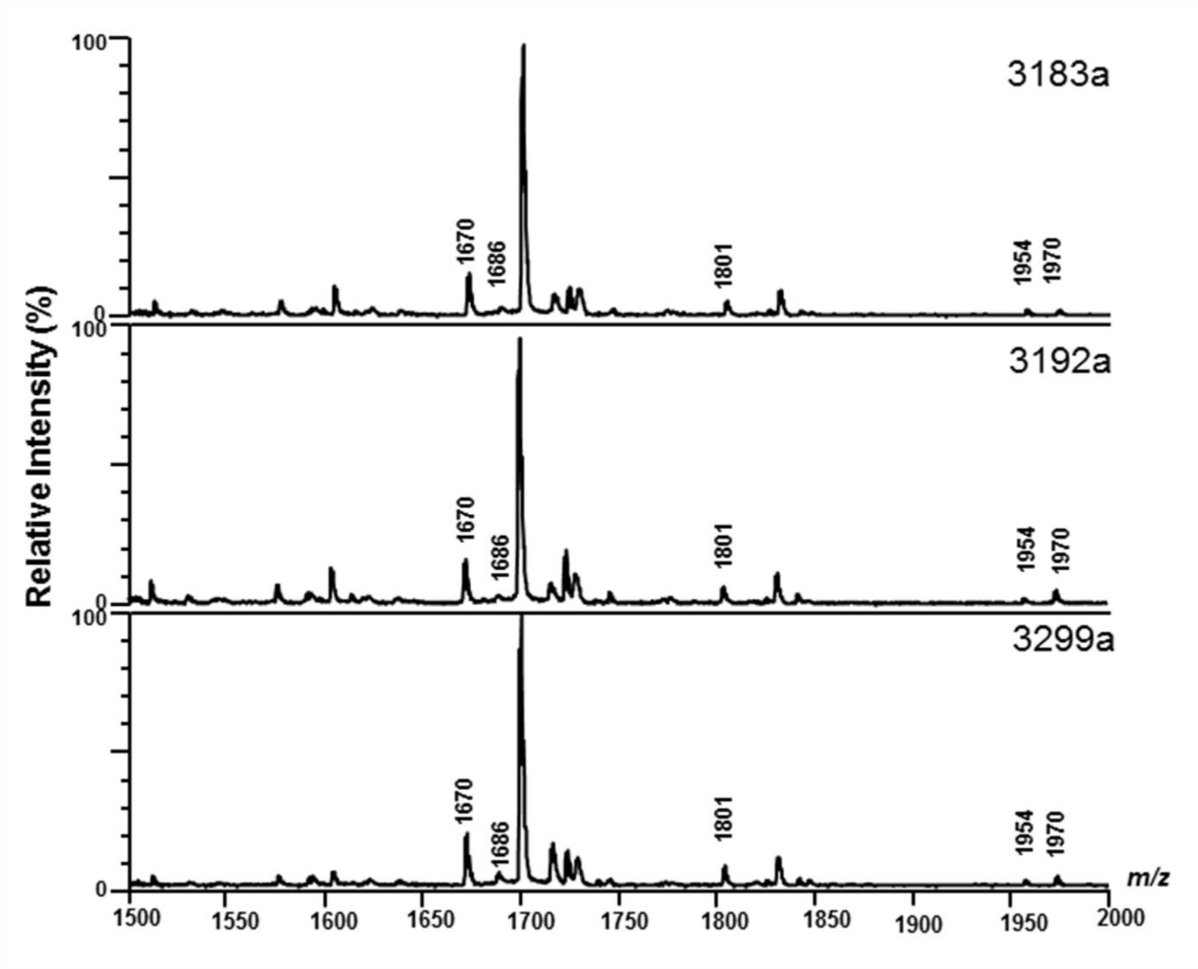

Relative Intensity (%)
100
0
3183a
3192a
3299a
1670
1686
1801
1954
1970
m/z
1500
1550
1600
1650
1700
1750
1800
1850
1900
1950
2000
