# Supplementary material for: Comprehensive analysis of clinical Burkholderia pseudomallei isolates demonstrates conservation of unique lipid A structure and TLR4-dependent innate immune activation
Source: PLoS Negl Trop Dis. 2018 Feb 23;12(2):e0006287. doi: 10.1371/journal.pntd.0006287 (PMC5842036; doi:10.1371/journal.pntd.0006287)
Supplement: S3 Fig — (PDF) [file pntd.0006287.s003.pdf]

S3 Fig. MALDI-TOF lipid A spectra of clinical isolates of *B. pseudomallei* from 68 Thai patients with relapse infection

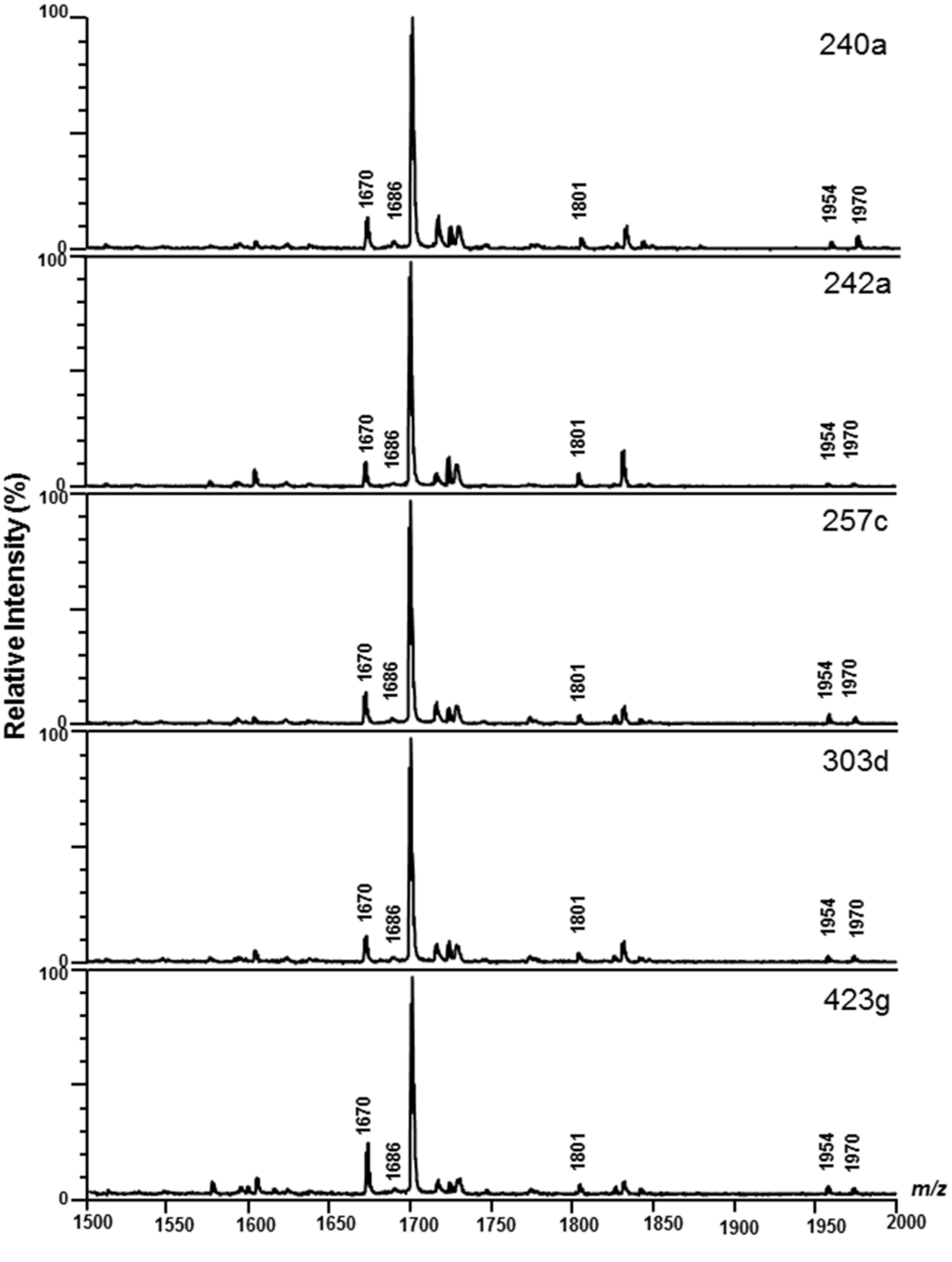

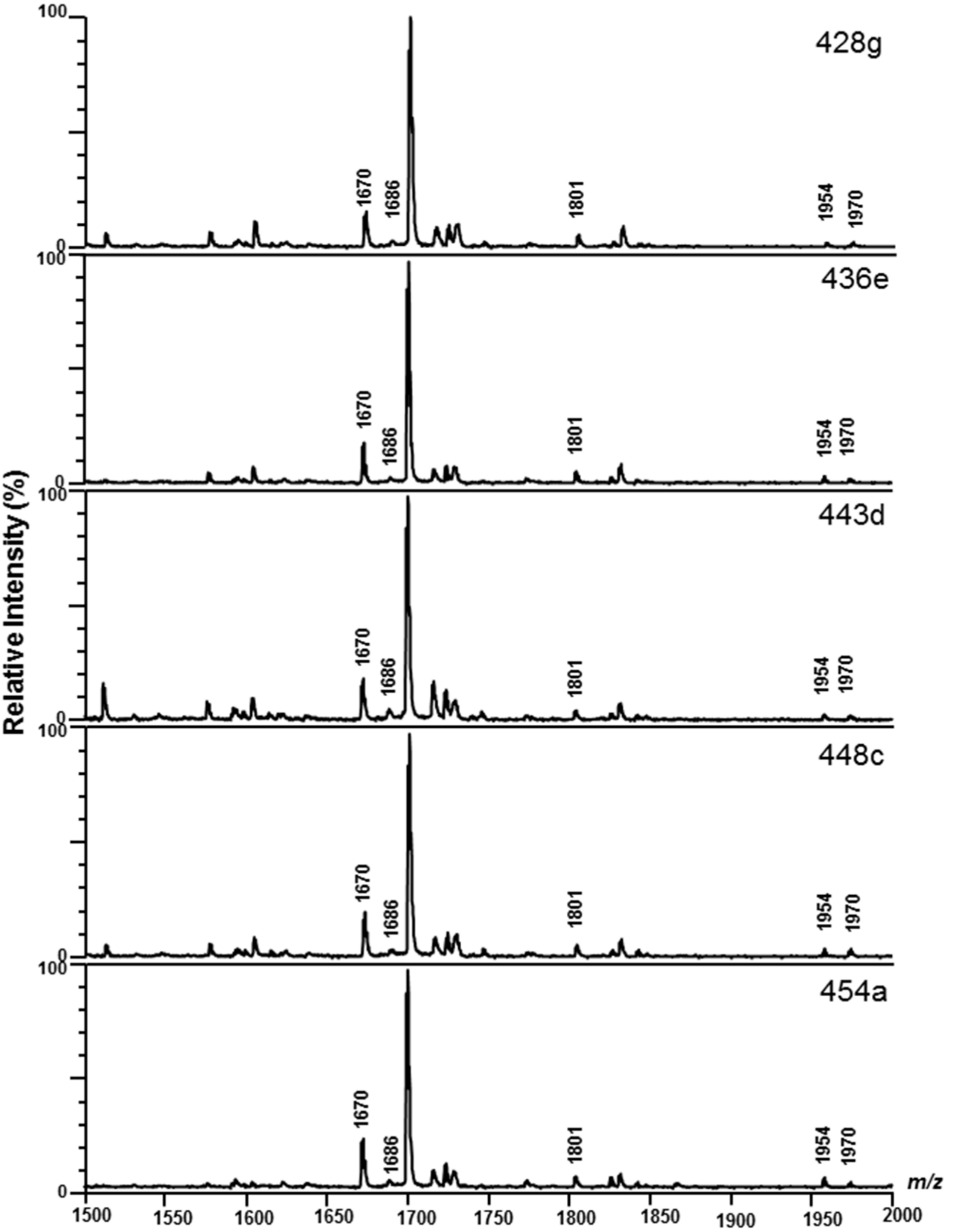

Relative Intensity (%)
100
0
428g
436e
443d
448c
454a
1670
1686
1801
1954
1970
m/z
1500
1550
1600
1650
1700
1750
1800
1850
1900
1950
2000

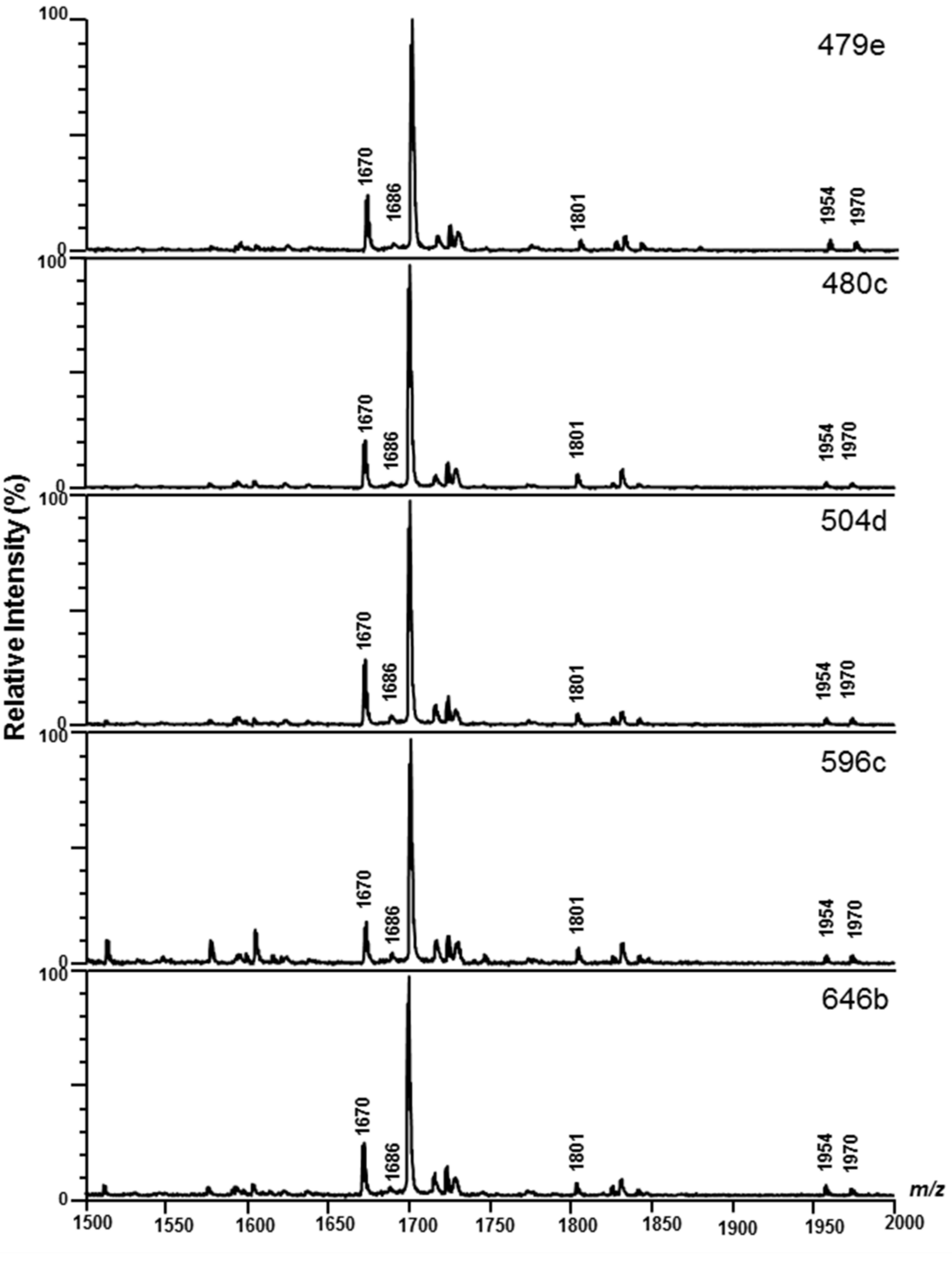

Relative Intensity (%)
100
0
479e
480c
504d
596c
646b
1670
1686
1801
1954
1970
m/z
1500
1550
1600
1650
1700
1750
1800
1850
1900
1950
2000

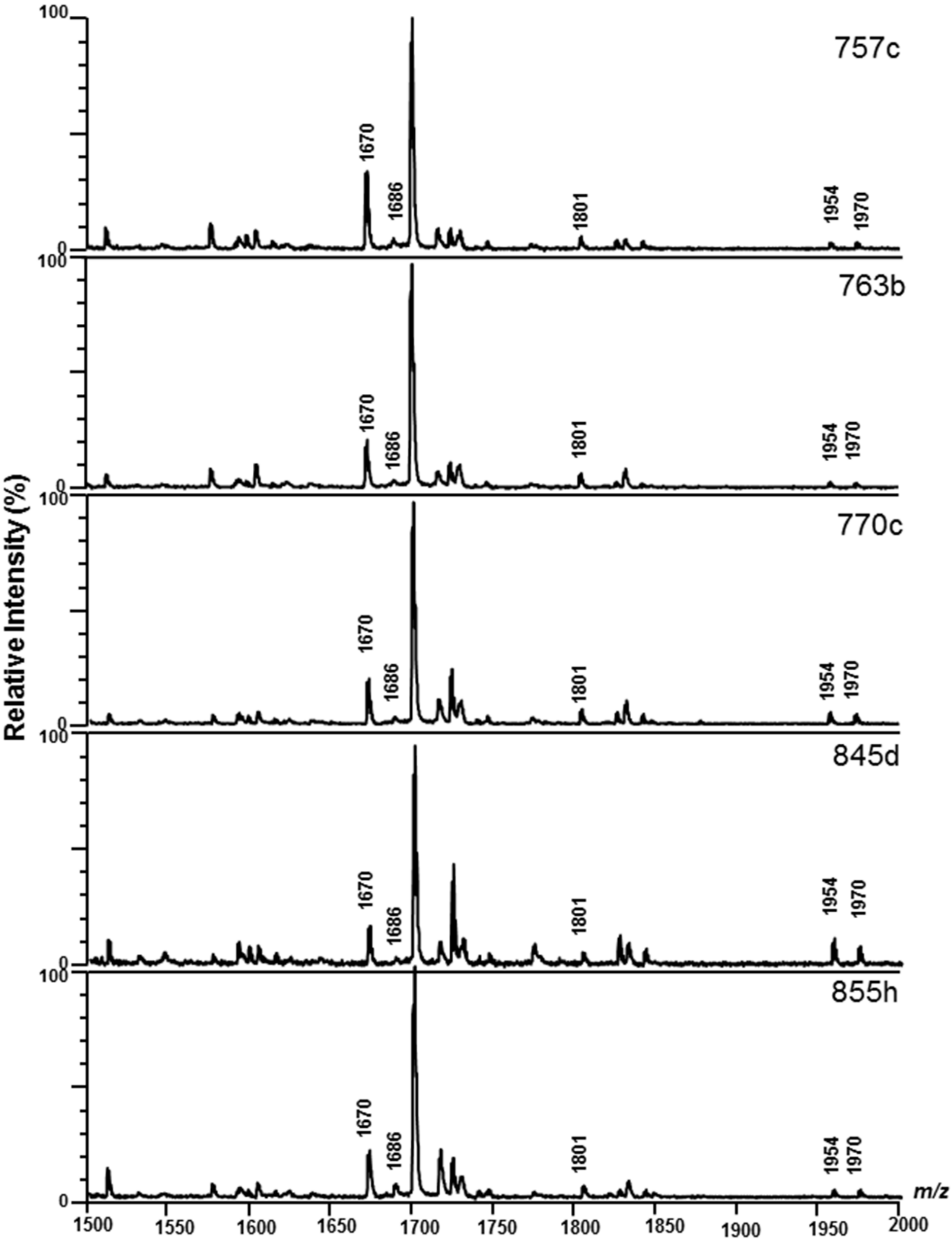

Relative Intensity (%)
757c
763b
770c
845d
855h
1670
1686
1801
1954
1970
100
0
m/z
1500
1550
1600
1650
1700
1750
1800
1850
1900
1950
2000

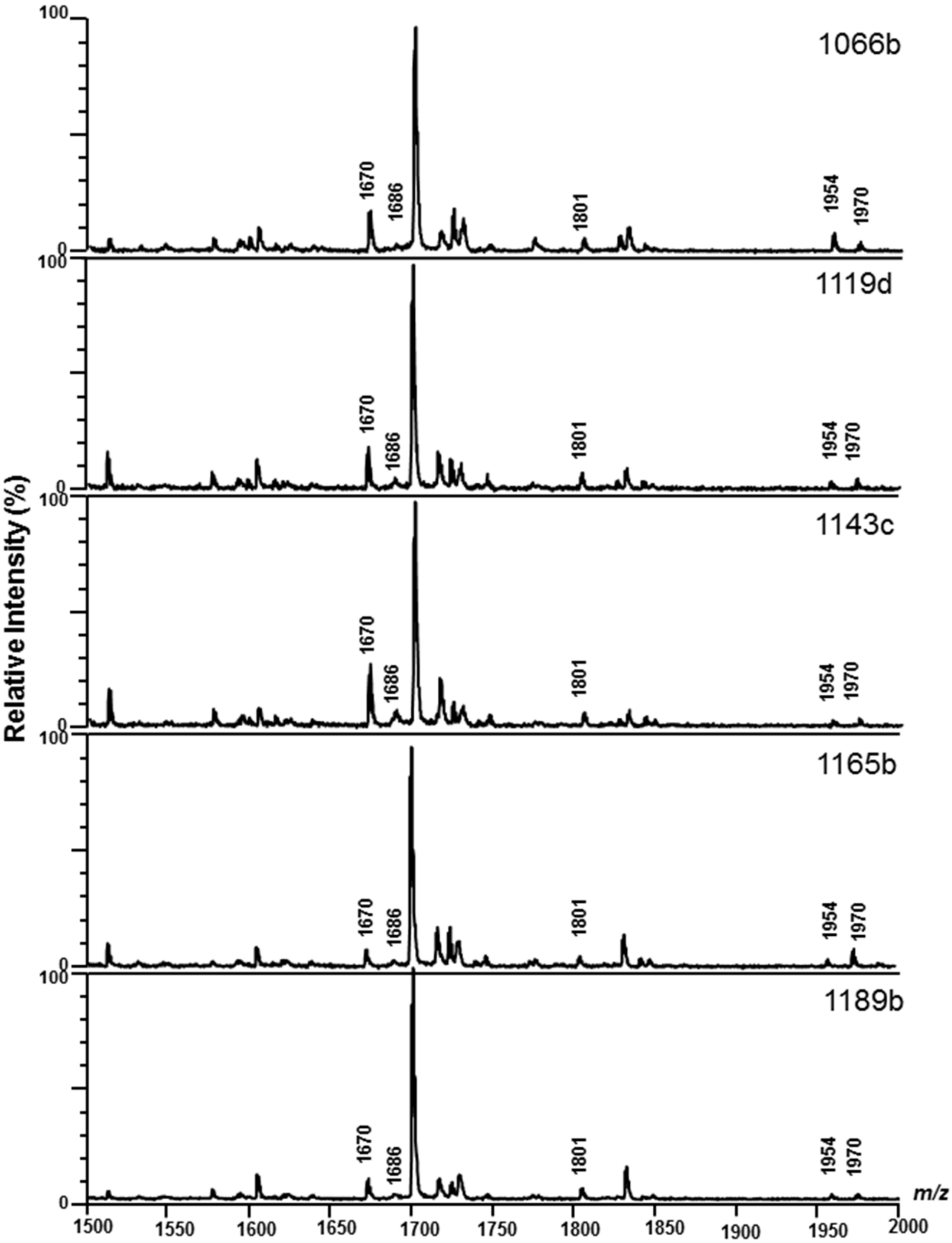

Relative Intensity (%)
1066b
1119d
1143c
1165b
1189b
1670
1686
1801
1954
1970
100
0
m/z
1500
1550
1600
1650
1700
1750
1800
1850
1900
1950
2000

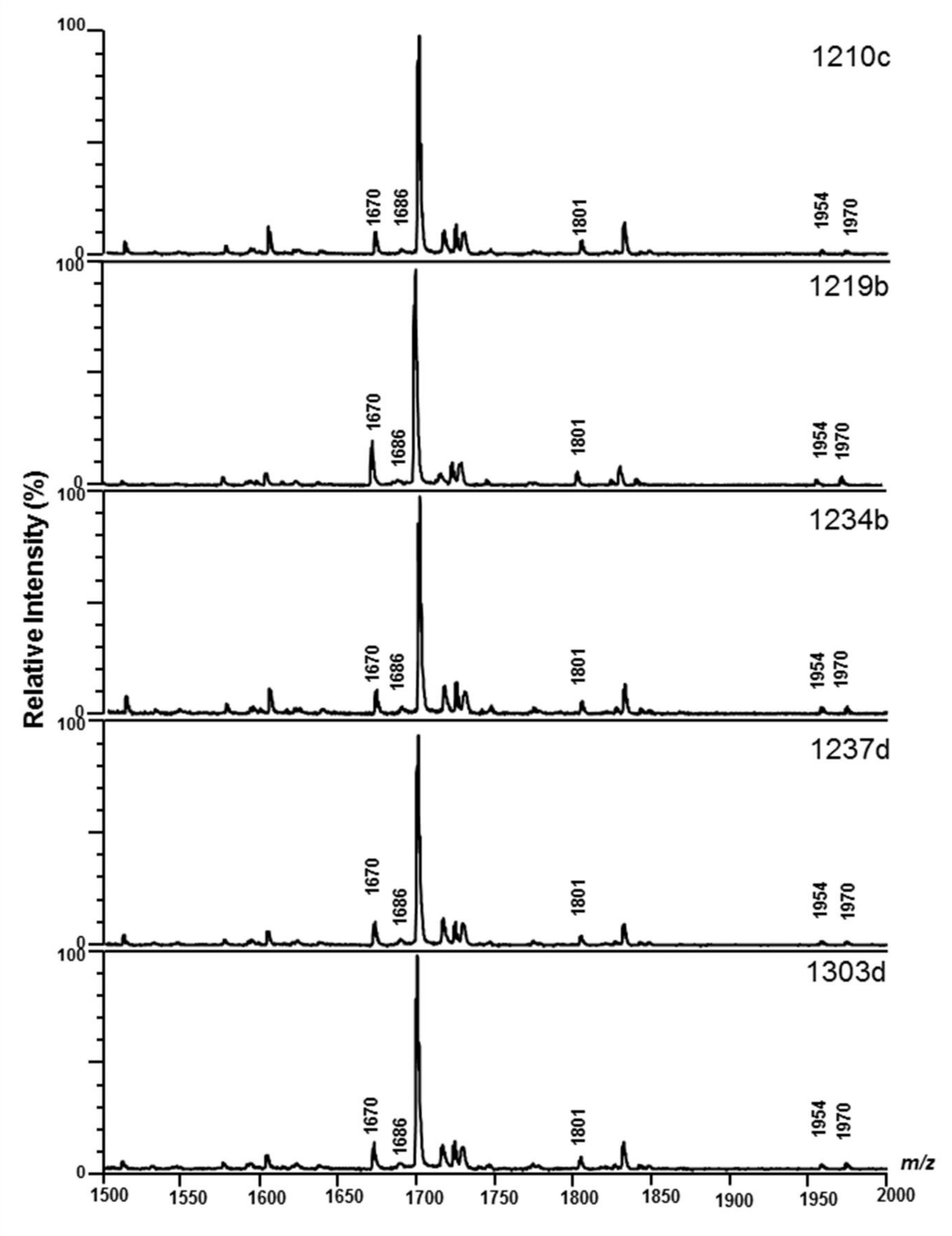

1210c
1219b
1234b
1237d
1303d
1670
1686
1801
1954
1970
Relative Intensity (%)
100
0
m/z
1500
1550
1600
1650
1700
1750
1800
1850
1900
1950
2000

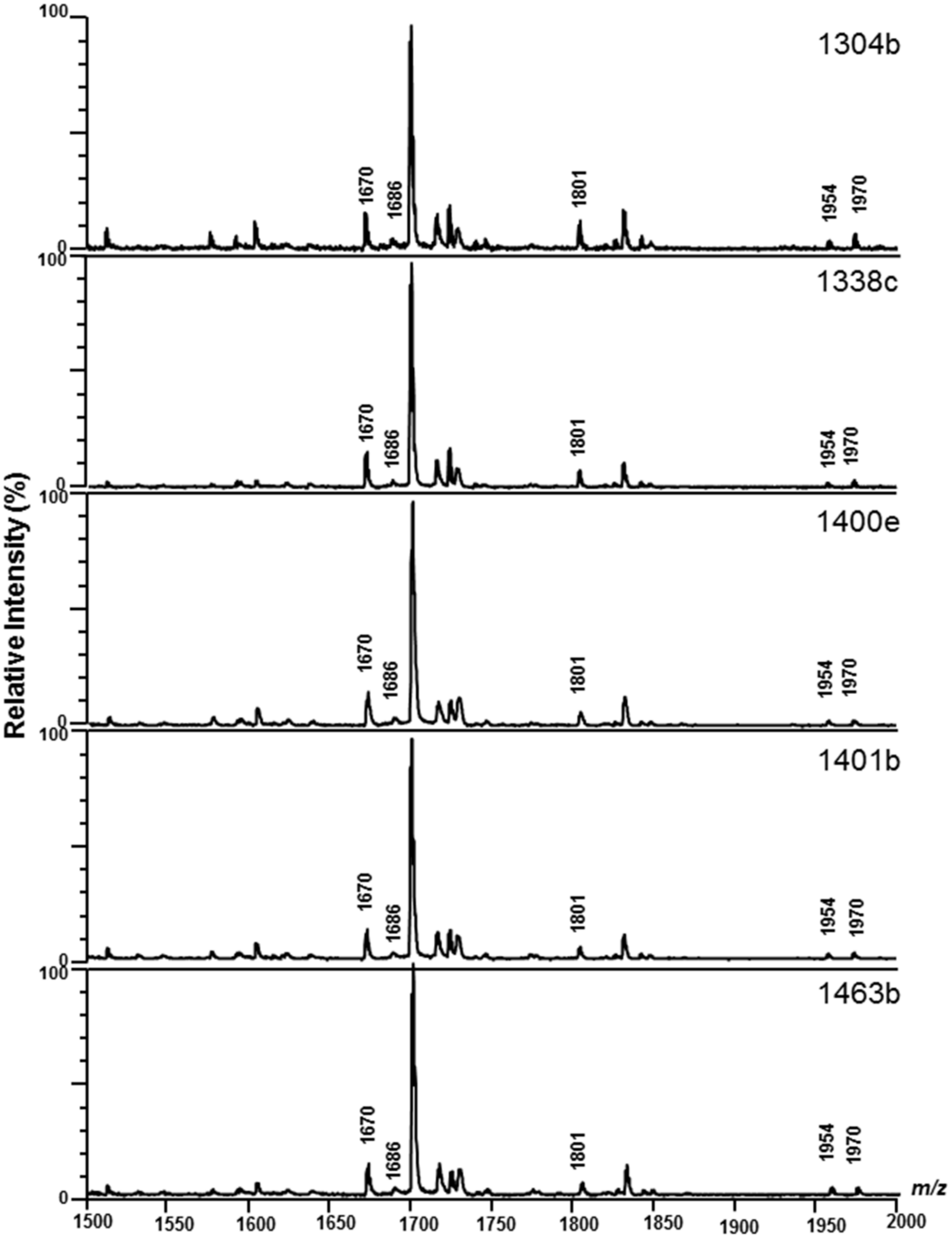

Relative Intensity (%)
1304b
1338c
1400e
1401b
1463b
1670
1686
1801
1954
1970
m/z
100
0
1500
1550
1600
1650
1700
1750
1800
1850
1900
1950
2000

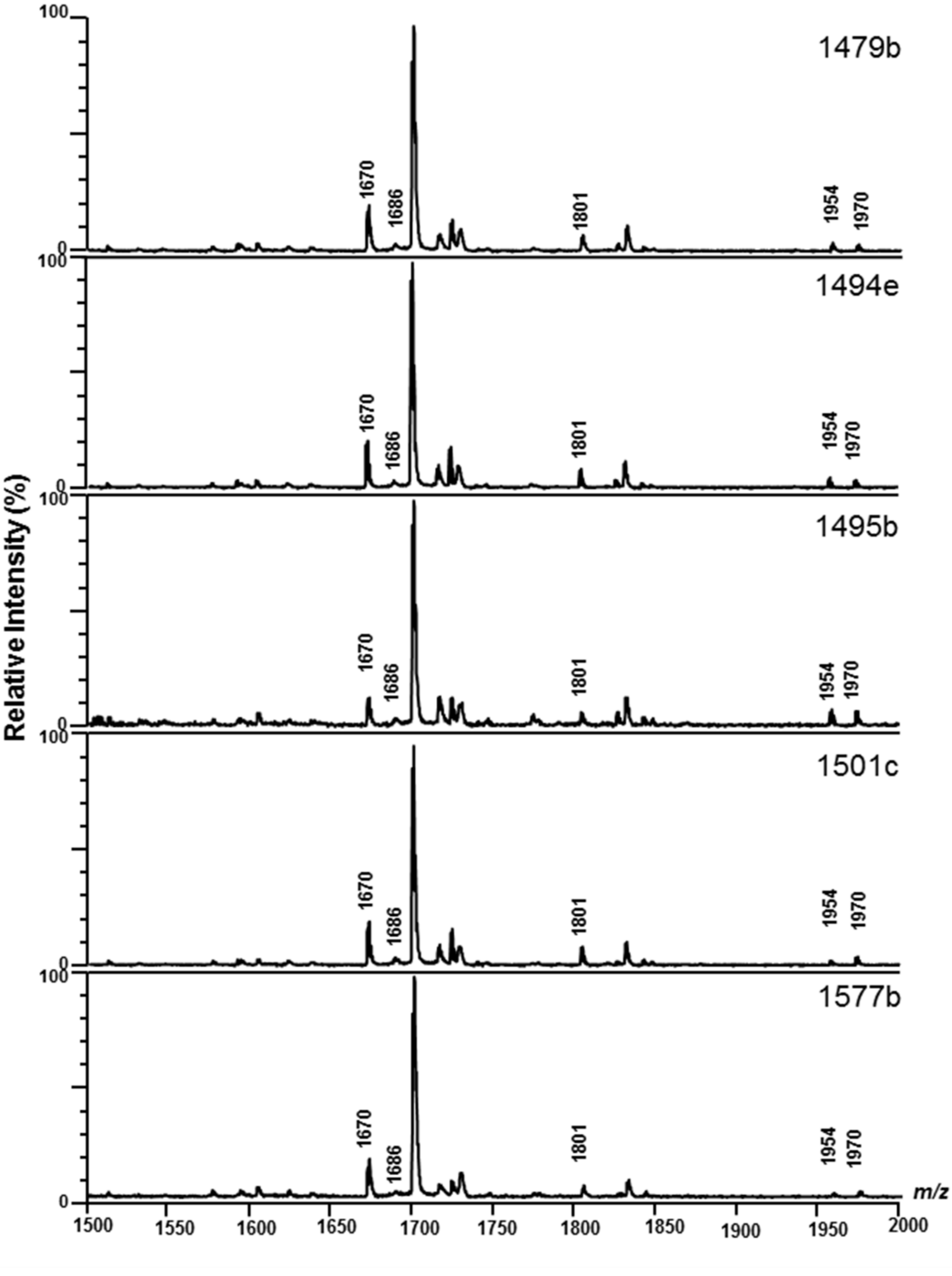

Relative Intensity (%)
1479b
1494e
1495b
1501c
1577b
1670
1686
1801
1954
1970
100
0
m/z
1500
1550
1600
1650
1700
1750
1800
1850
1900
1950
2000

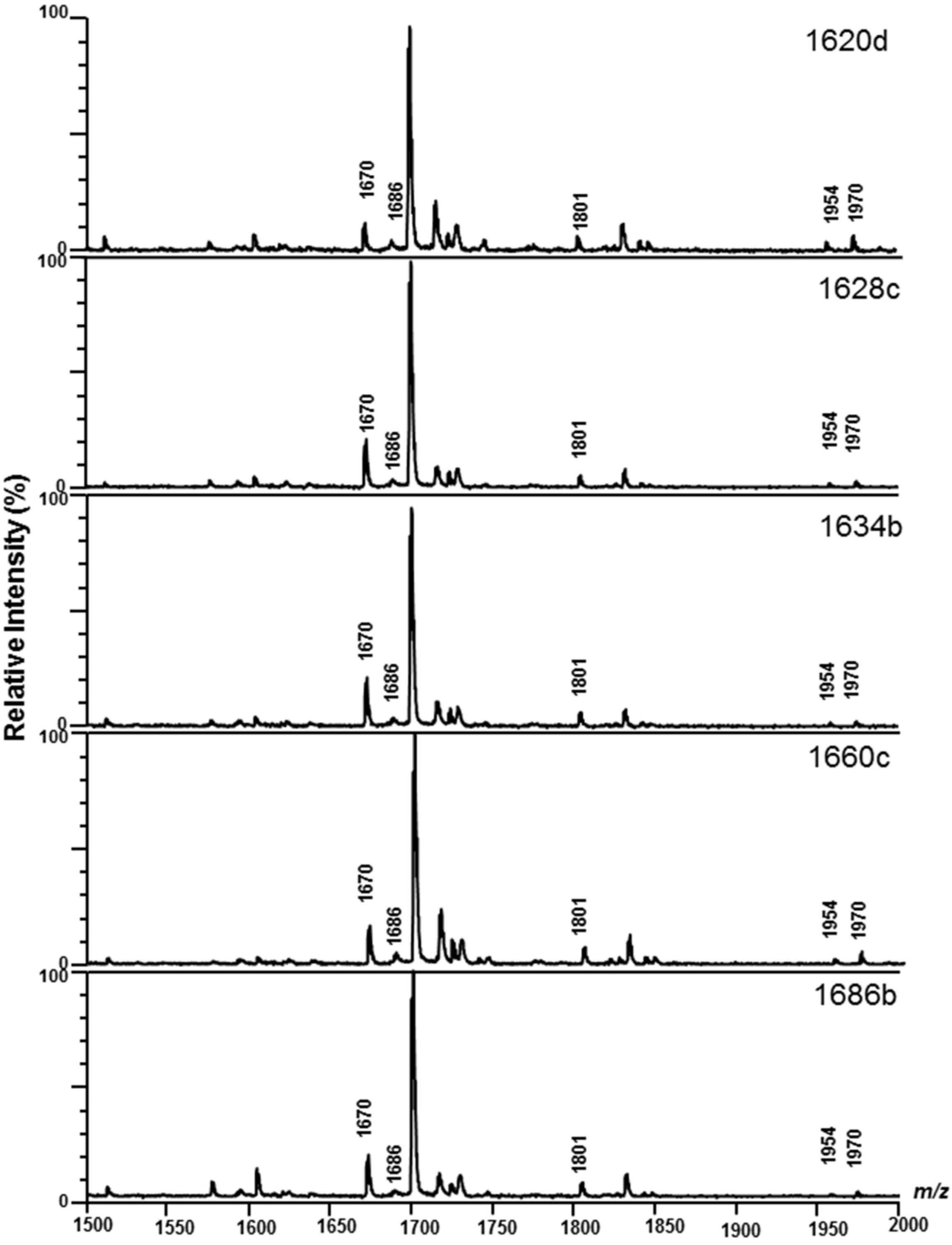

1620d
1628c
1634b
1660c
1686b
1670
1686
1801
1954
1970
100
0
Relative Intensity (%)
m/z
1500
1550
1600
1650
1700
1750
1800
1850
1900
1950
2000

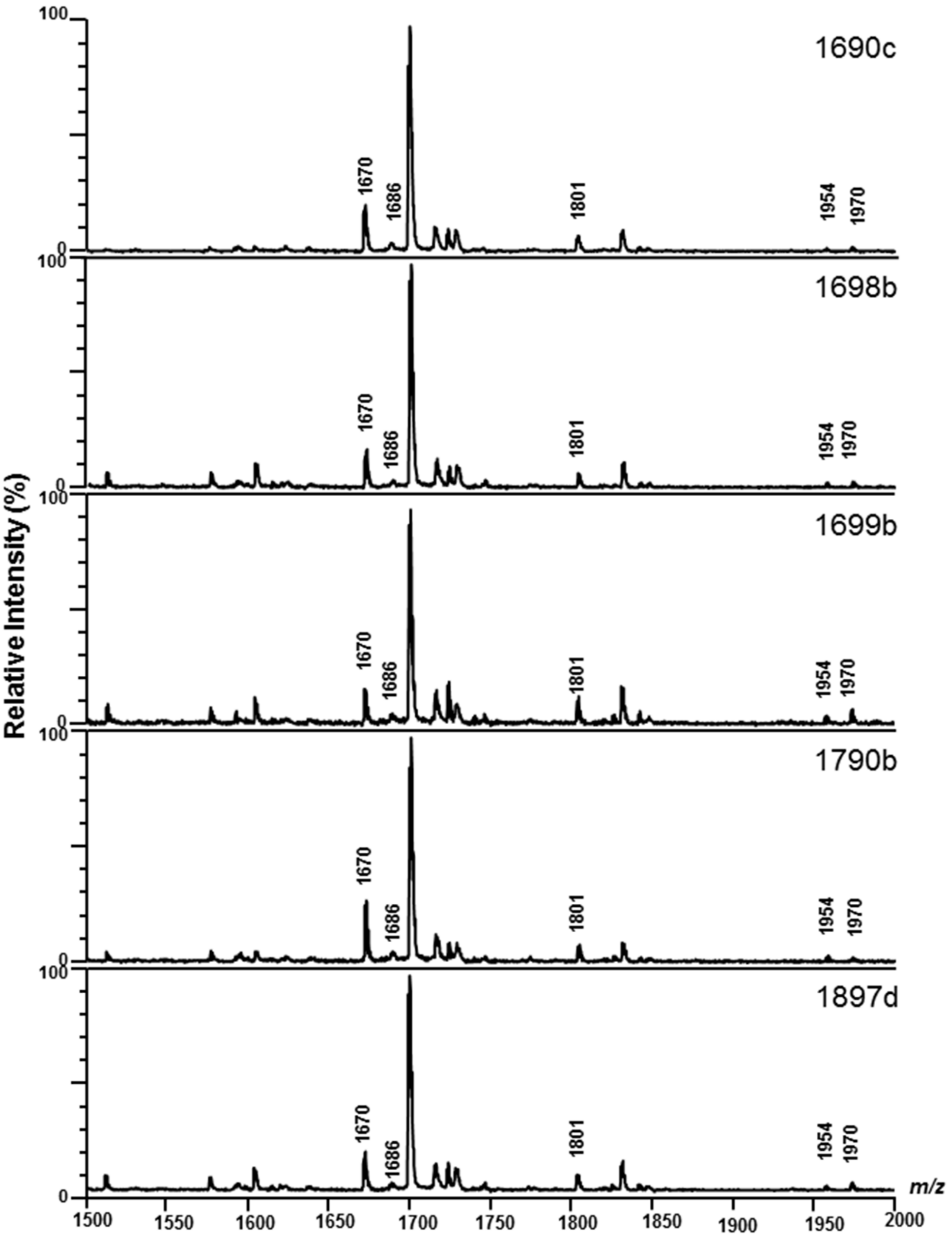

Relative Intensity (%)
100
0
1690c
1698b
1699b
1790b
1897d
1670
1686
1801
1954
1970
m/z
1500
1550
1600
1650
1700
1750
1800
1850
1900
1950
2000

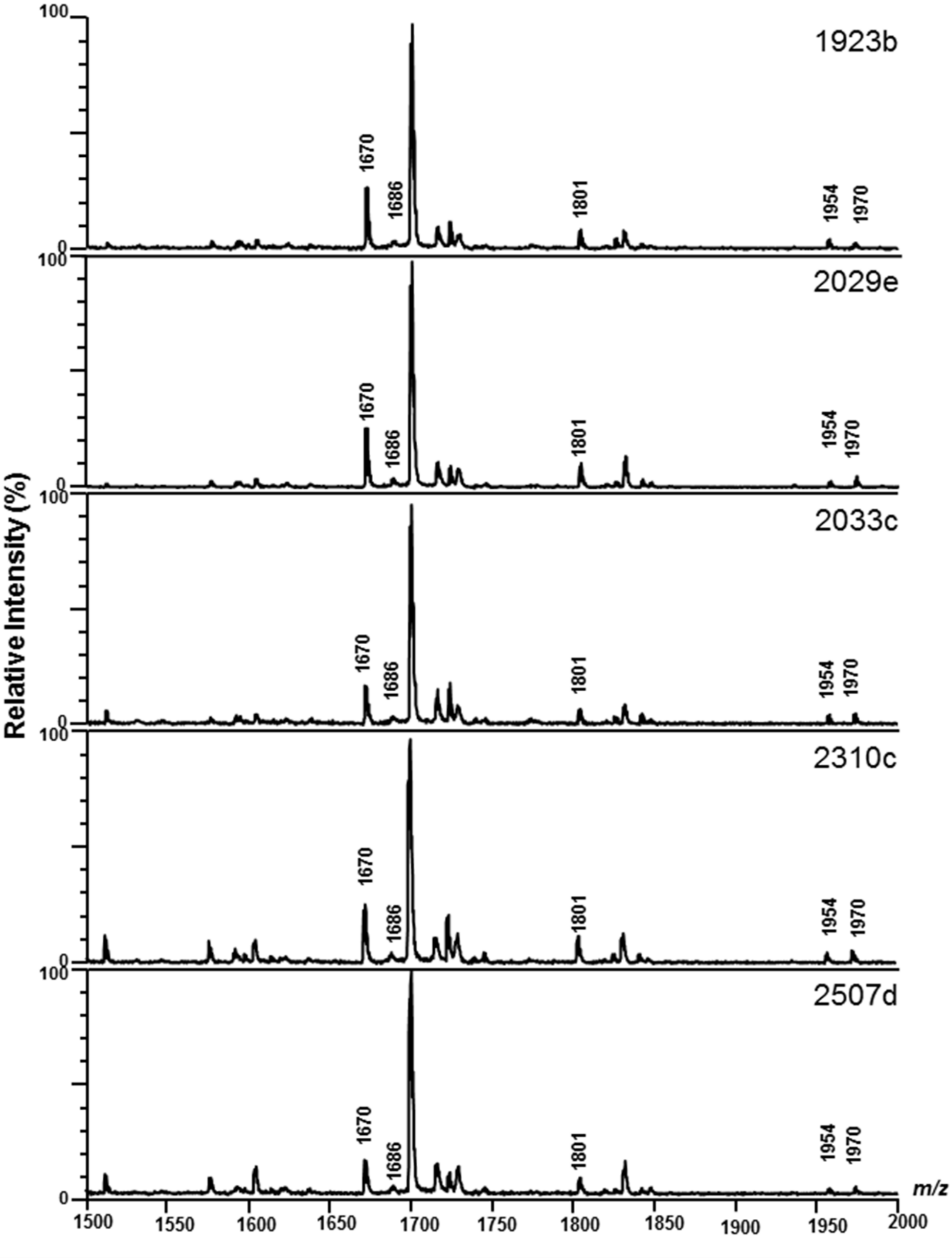

Relative Intensity (%)
100
0
1923b
2029e
2033c
2310c
2507d
1670
1686
1801
1954
1970
m/z
1500
1550
1600
1650
1700
1750
1800
1850
1900
1950
2000

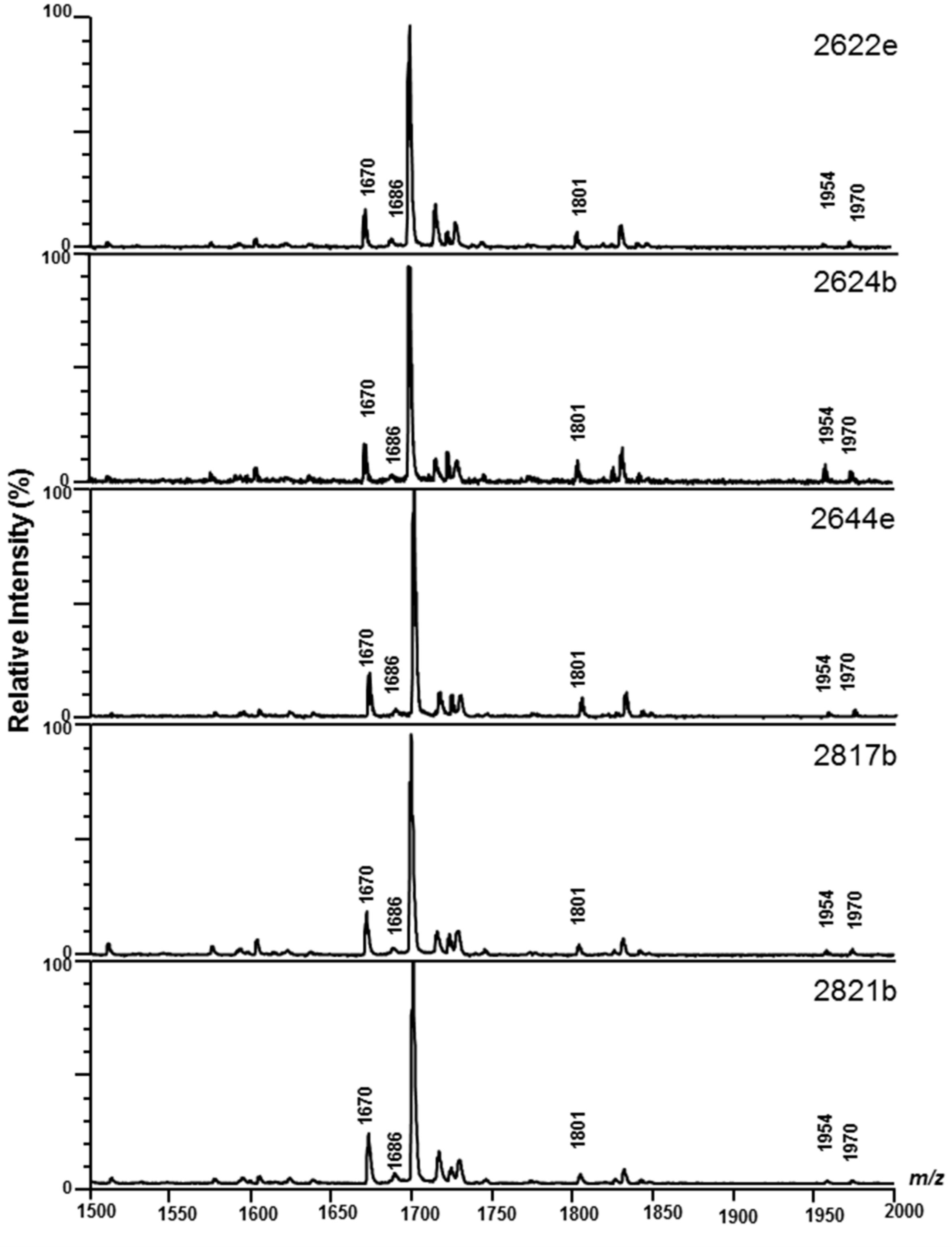

Relative Intensity (%)
2622e
2624b
2644e
2817b
2821b
1670
1686
1801
1954
1970
100
0
m/z
1500
1550
1600
1650
1700
1750
1800
1850
1900
1950
2000

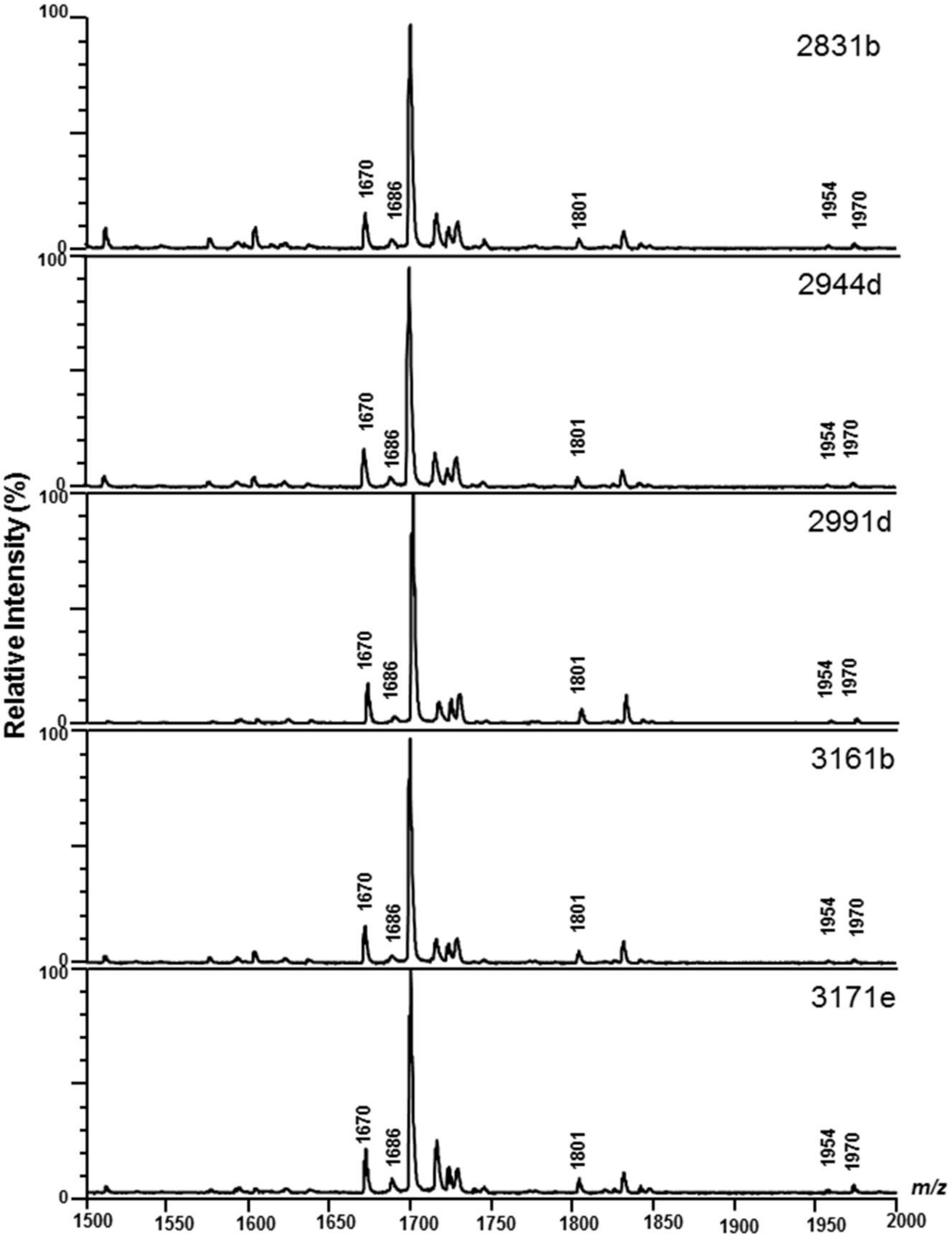

Relative Intensity (%)
100
0
2831b
2944d
2991d
3161b
3171e
1670
1686
1801
1954
1970
m/z
1500
1550
1600
1650
1700
1750
1800
1850
1900
1950
2000

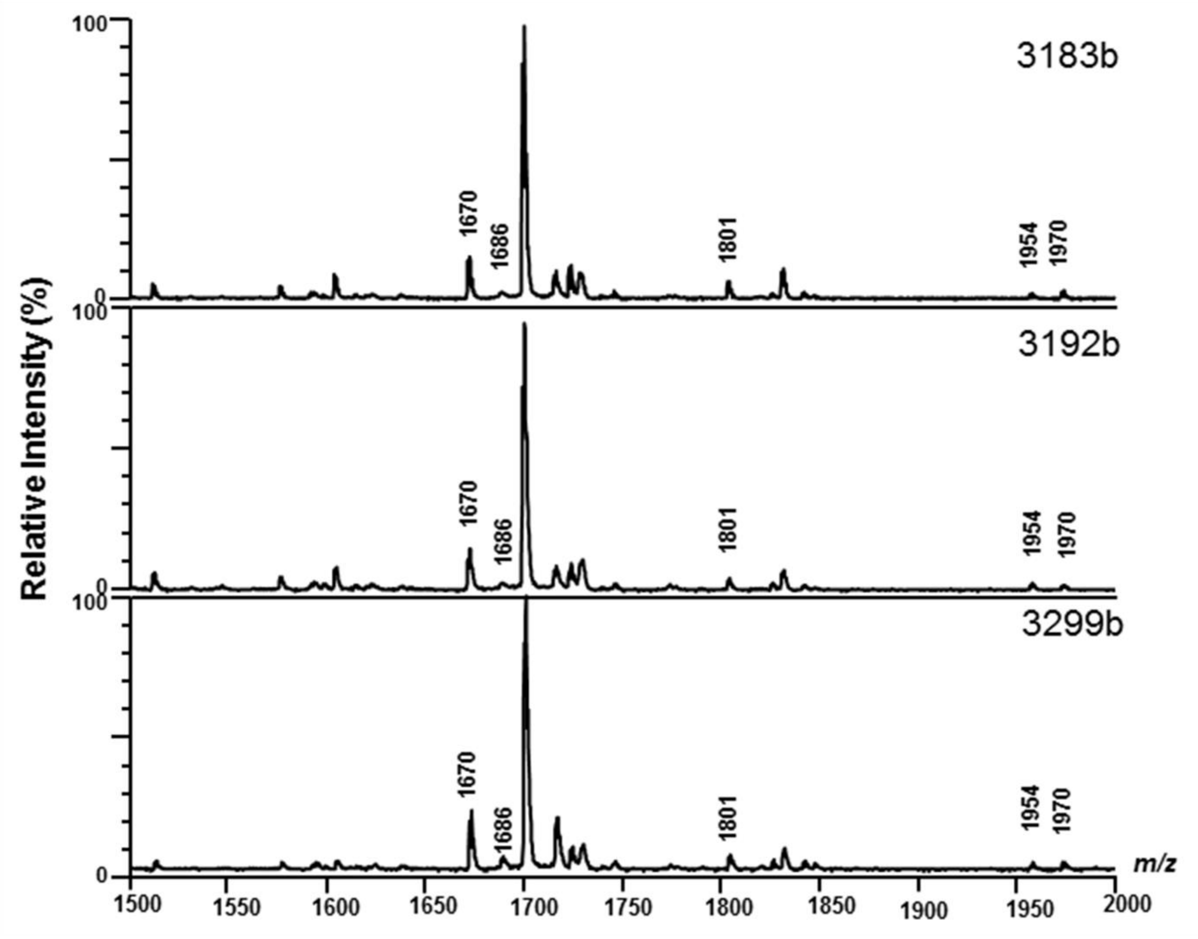

Relative Intensity (%)
3183b
3192b
3299b
1670
1686
1801
1954
1970
100
0
m/z
1500
1550
1600
1650
1700
1750
1800
1850
1900
1950
2000
