# Supplementary material for: Comprehensive analysis of clinical Burkholderia pseudomallei isolates demonstrates conservation of unique lipid A structure and TLR4-dependent innate immune activation
Source: PLoS Negl Trop Dis. 2018 Feb 23;12(2):e0006287. doi: 10.1371/journal.pntd.0006287 (PMC5842036; doi:10.1371/journal.pntd.0006287)
Supplement: S4 Fig — (PDF) [file pntd.0006287.s004.pdf]

S4 Fig. MALDI-TOF lipid A spectra of 35 individual colonies of *B. pseudomallei* from 7 clinical specimens (5 colonies for each specimens) from a Thai patient with acute melioidosis

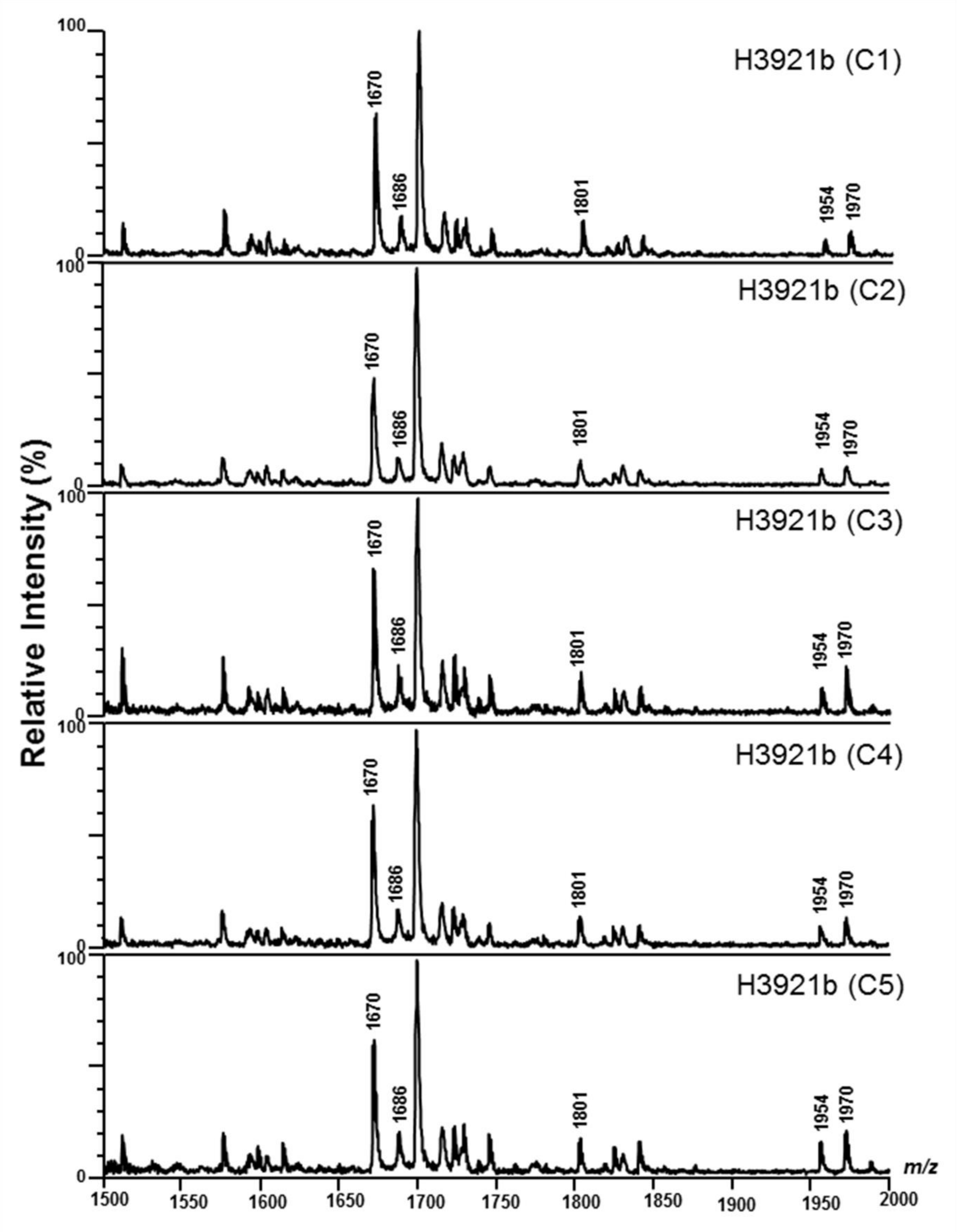

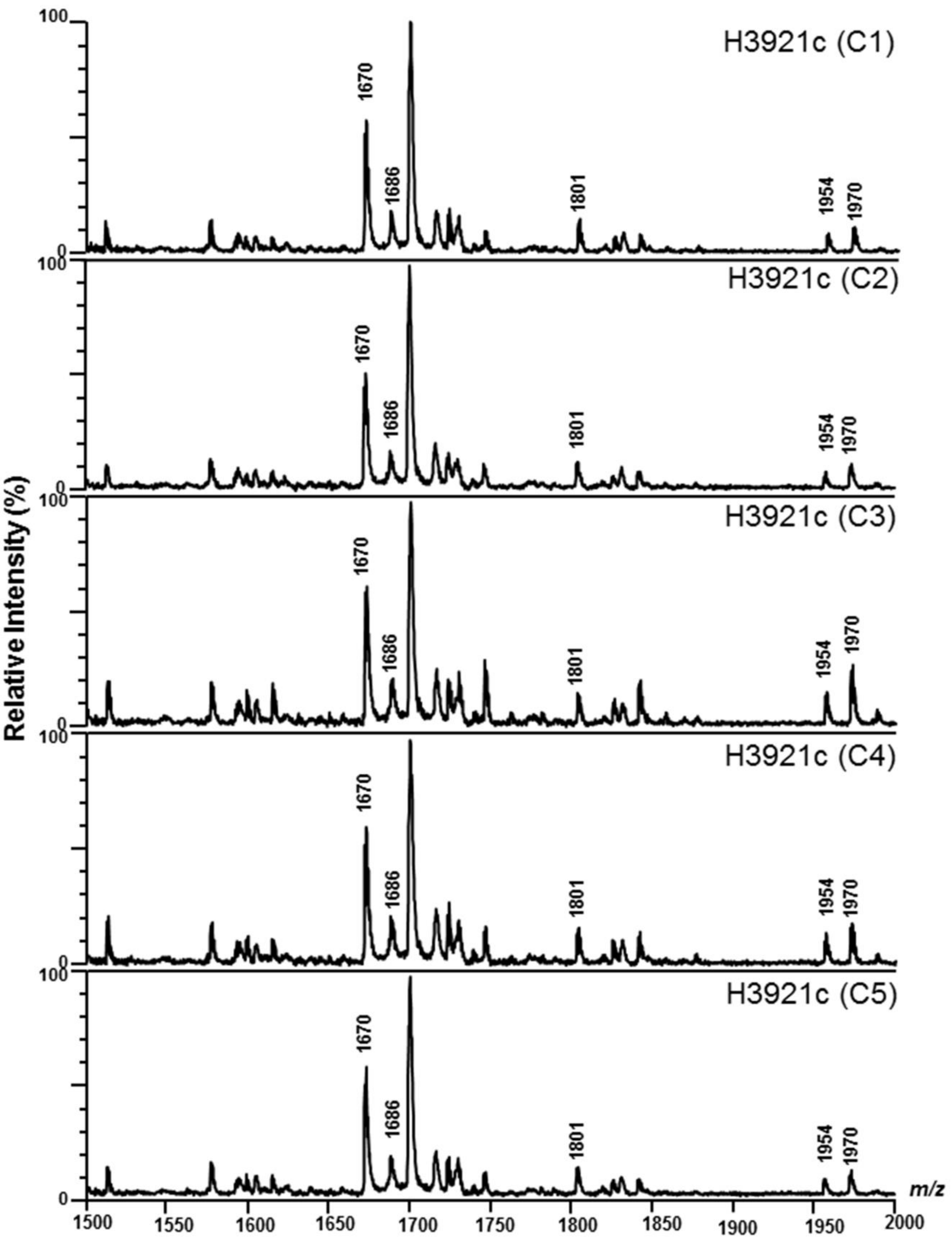
H3921c (C1)
H3921c (C2)
H3921c (C3)
H3921c (C4)
H3921c (C5)
1670
1686
1801
1954
1970
Relative Intensity (%)
100
0
m/z
1500
1550
1600
1650
1700
1750
1800
1850
1900
1950
2000

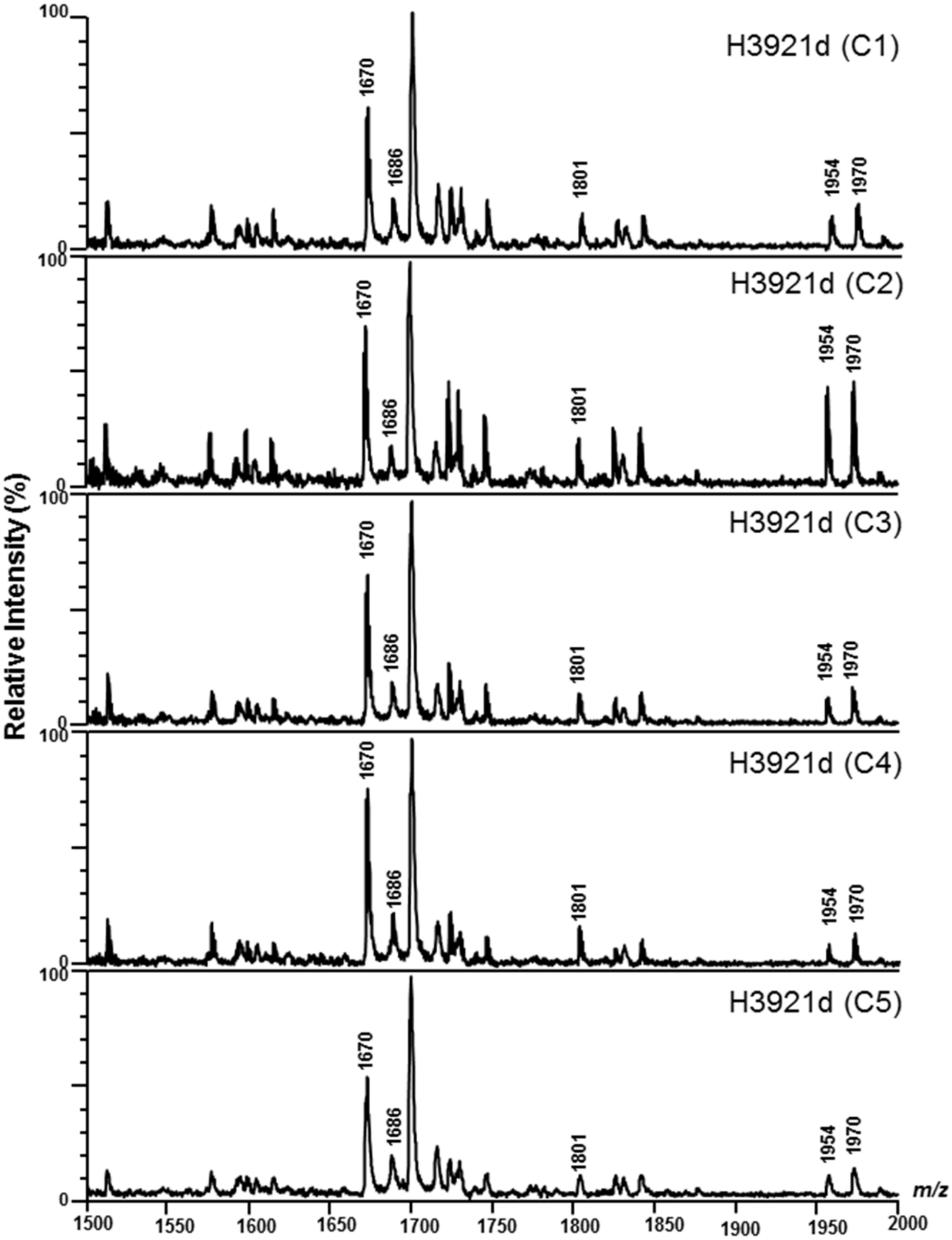
Relative Intensity (%)
H3921d (C1)
H3921d (C2)
H3921d (C3)
H3921d (C4)
H3921d (C5)
1670
1686
1801
1954
1970
100
0
m/z
1500
1550
1600
1650
1700
1750
1800
1850
1900
1950
2000

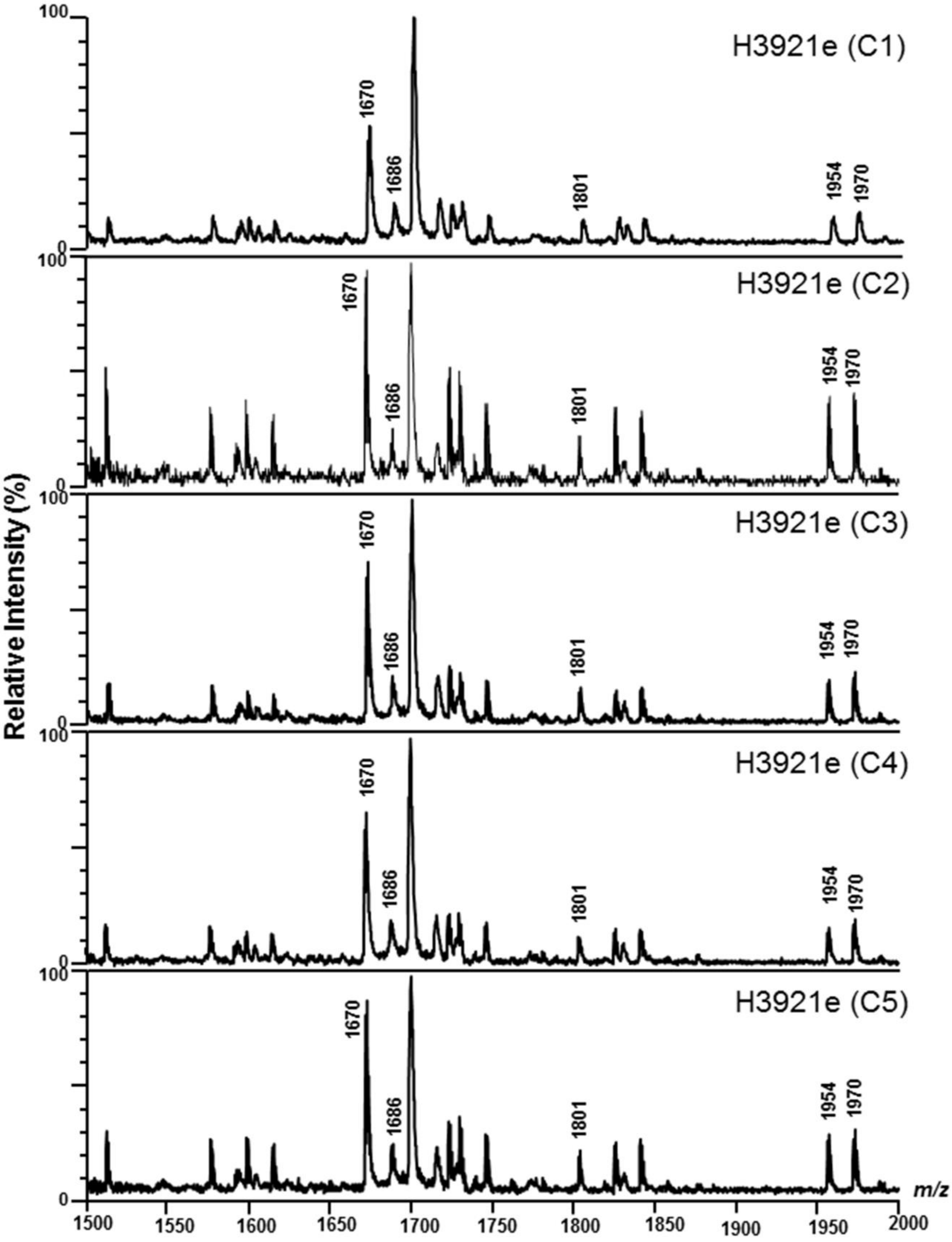

H3921e (C1)
H3921e (C2)
H3921e (C3)
H3921e (C4)
H3921e (C5)
1670
1686
1801
1954
1970
Relative Intensity (%)
100
0
m/z
1500
1550
1600
1650
1700
1750
1800
1850
1900
1950
2000

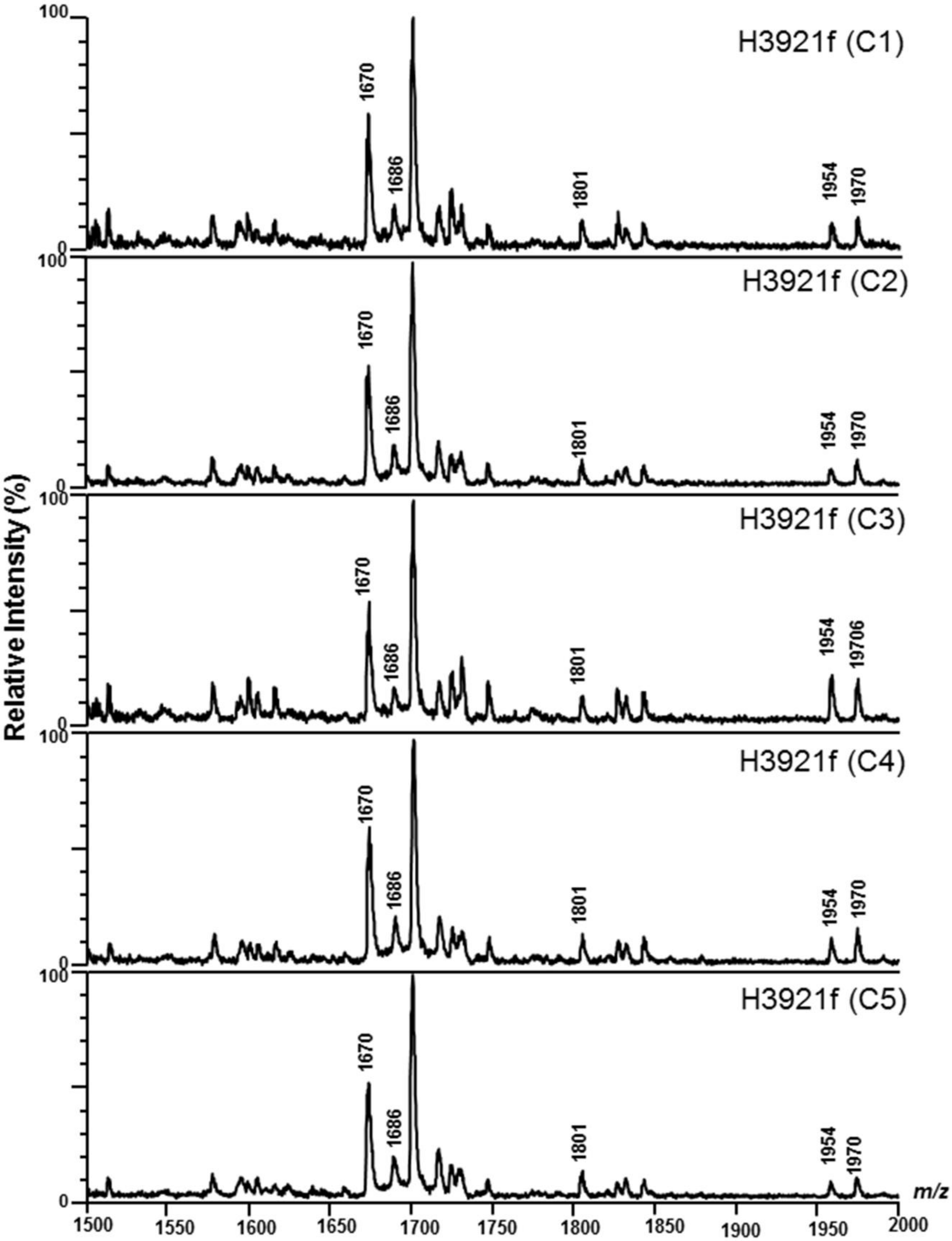

H3921f (C1)
H3921f (C2)
H3921f (C3)
H3921f (C4)
H3921f (C5)
1670
1686
1801
1954
1970
19706
Relative Intensity (%)
100
0
m/z
1500
1550
1600
1650
1700
1750
1800
1850
1900
1950
2000

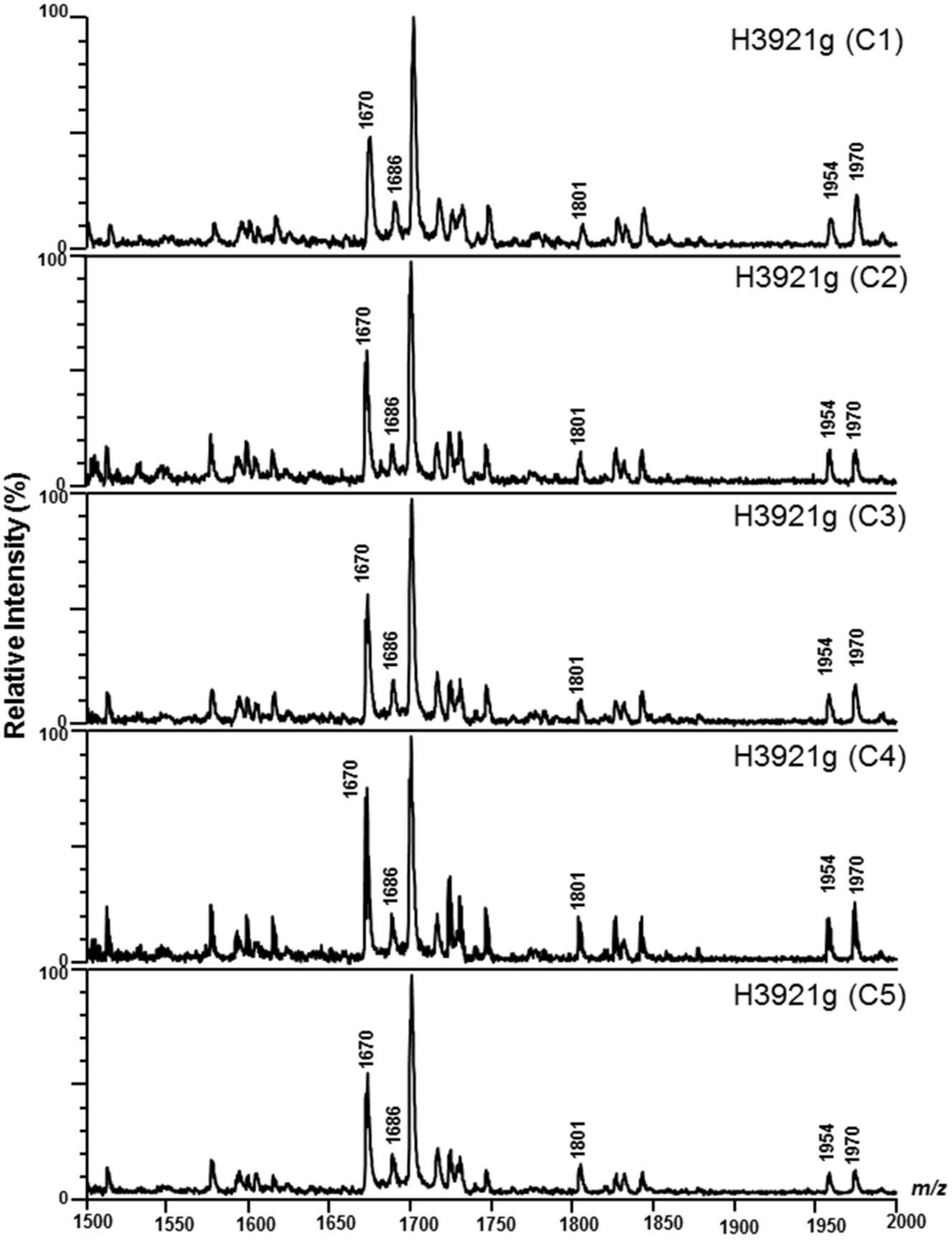

H3921g (C1)
H3921g (C2)
H3921g (C3)
H3921g (C4)
H3921g (C5)
1670
1686
1801
1954
1970
Relative Intensity (%)
100
0
m/z
1500
1550
1600
1650
1700
1750
1800
1850
1900
1950
2000

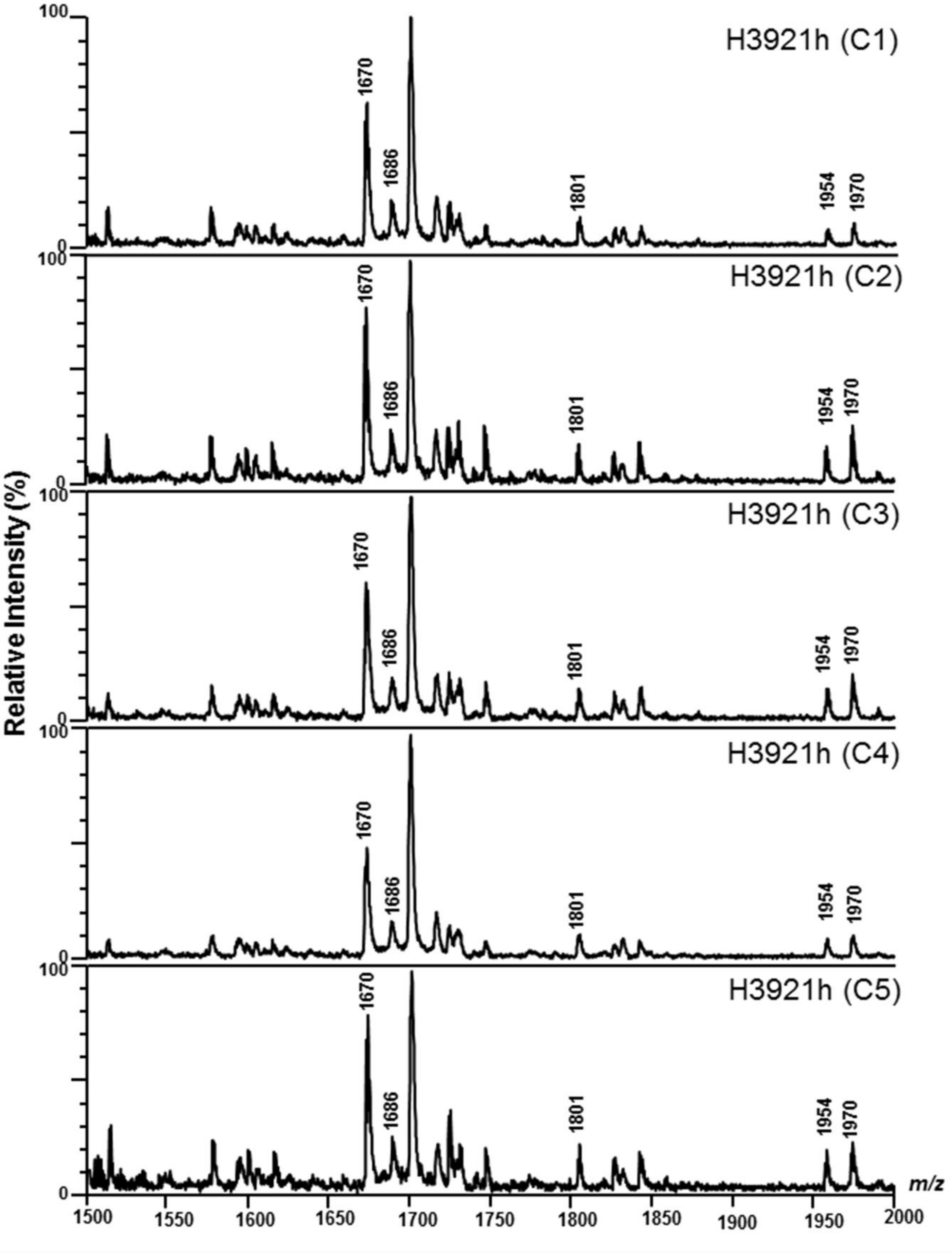
Relative Intensity (%)
H3921h (C1)
H3921h (C2)
H3921h (C3)
H3921h (C4)
H3921h (C5)
1670
1686
1801
1954
1970
100
0
m/z
1500
1550
1600
1650
1700
1750
1800
1850
1900
1950
2000
