# Supplementary material for: Comprehensive analysis of clinical Burkholderia pseudomallei isolates demonstrates conservation of unique lipid A structure and TLR4-dependent innate immune activation
Source: PLoS Negl Trop Dis. 2018 Feb 23;12(2):e0006287. doi: 10.1371/journal.pntd.0006287 (PMC5842036; doi:10.1371/journal.pntd.0006287)
Supplement: S5 Fig — (PDF) [file pntd.0006287.s005.pdf]

S5 Fig. MALDI-TOF lipid A spectra of clinical isolates of *B. pseudomallei* strain K96243 cultured in different laboratory conditions

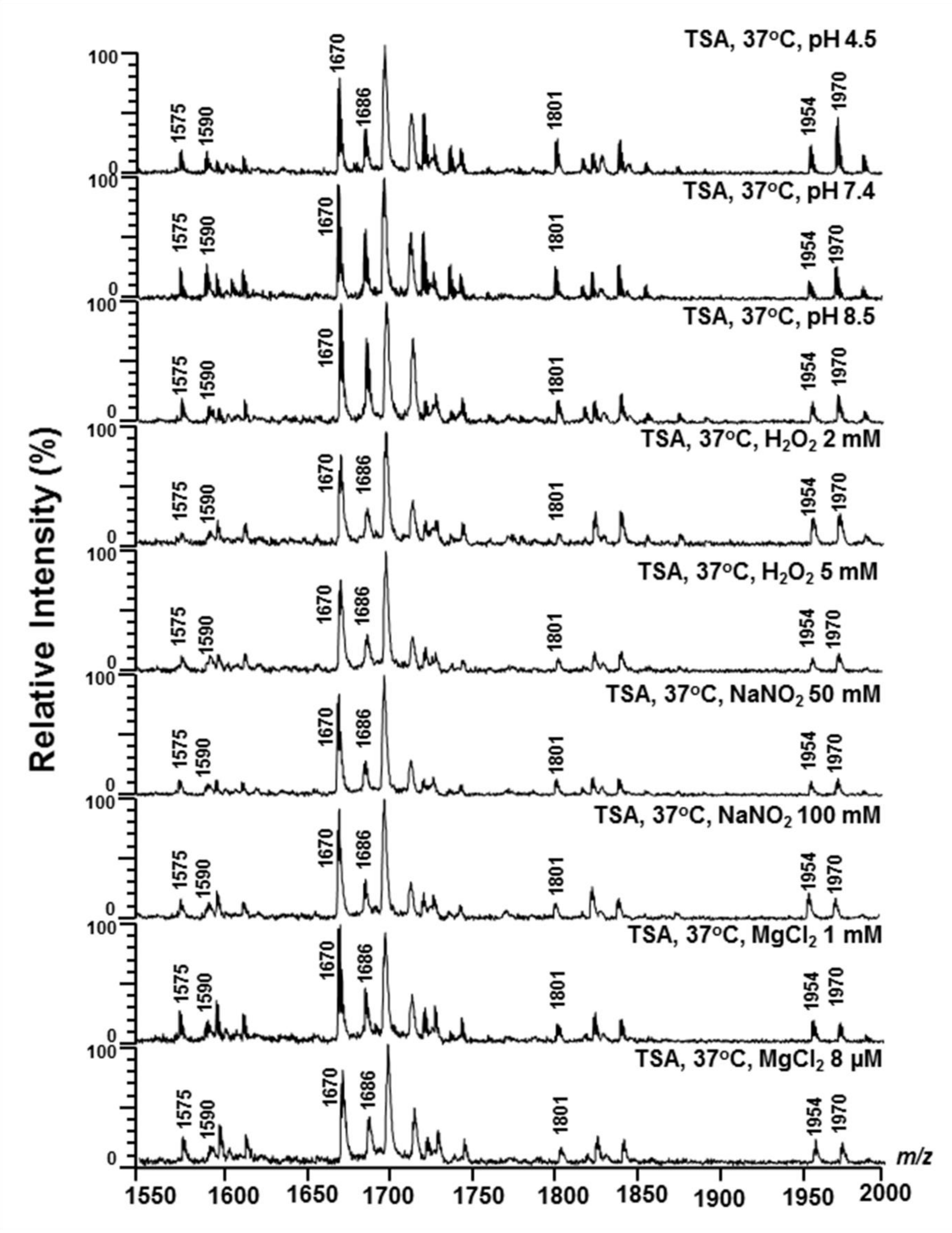

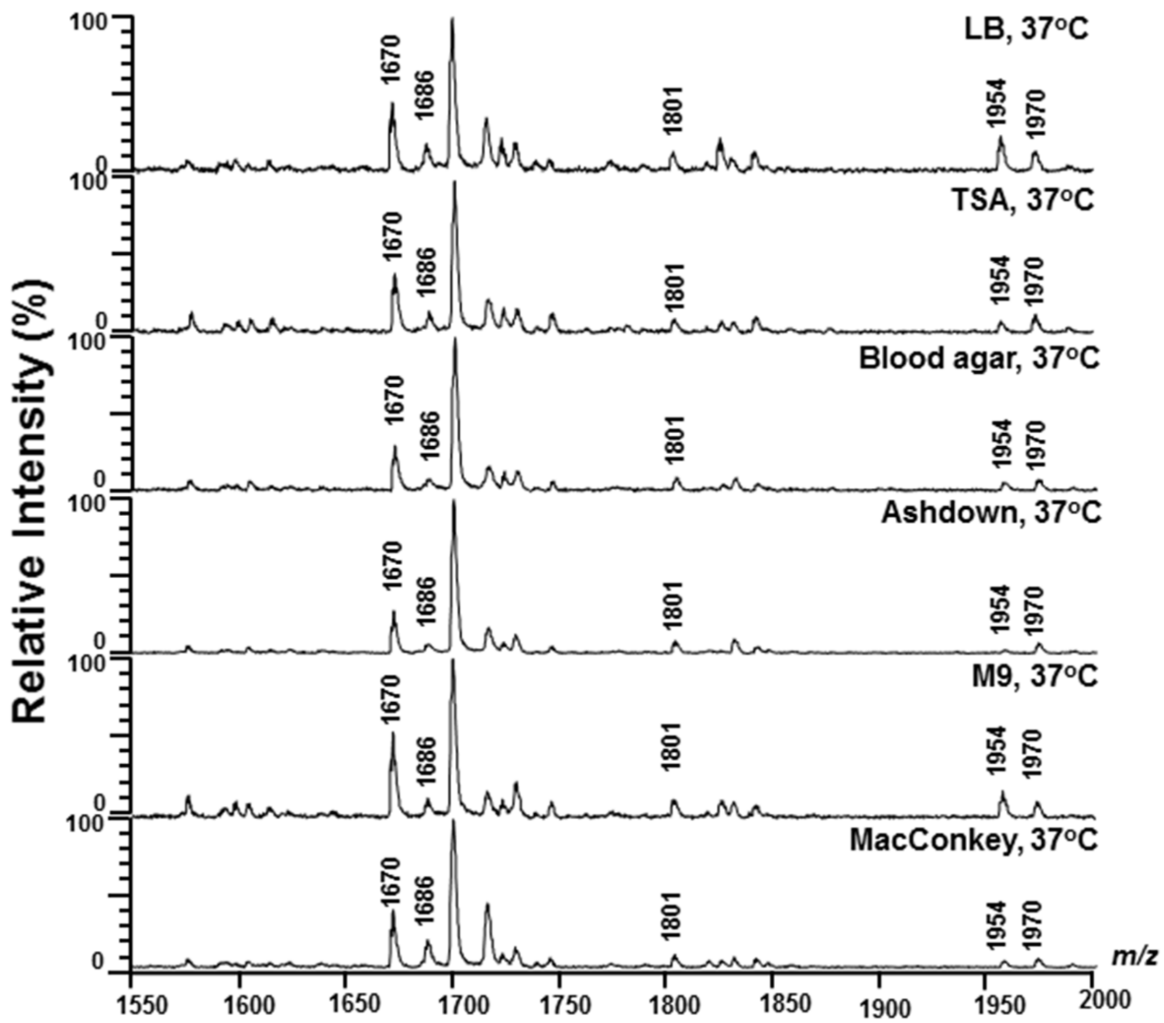

Relative Intensity (%)
LB, 37oC
TSA, 37oC
Blood agar, 37oC
Ashdown, 37oC
M9, 37oC
MacConkey, 37oC
1670
1686
1801
1954
1970
100
0
m/z
1550
1600
1650
1700
1750
1800
1850
1900
1950
2000

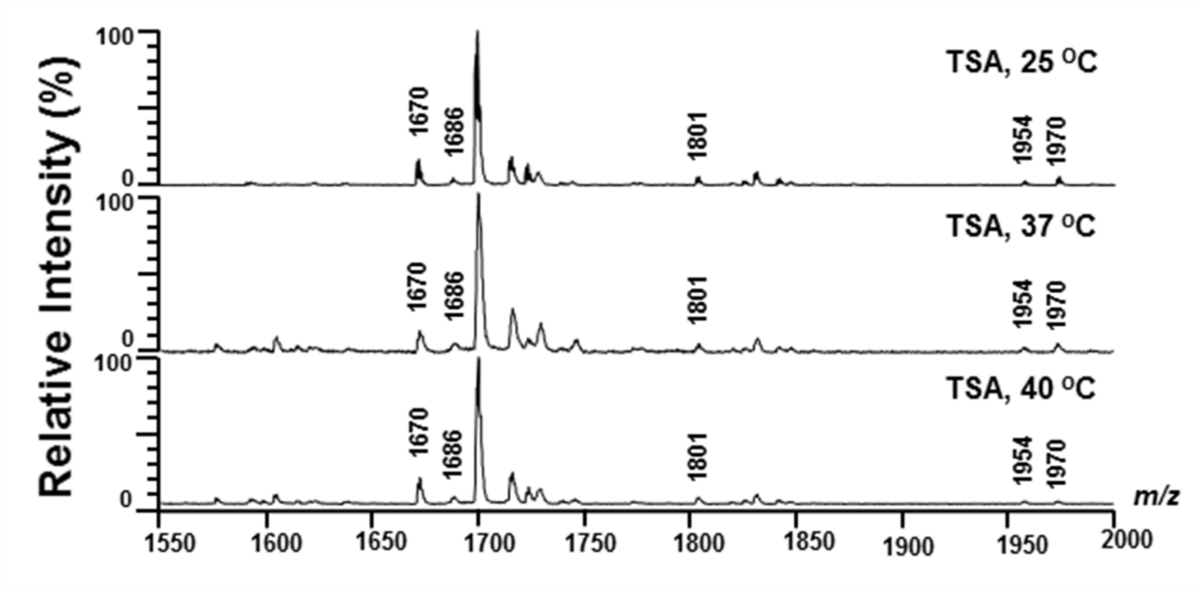
Relative Intensity (%)
TSA, 25 °C
TSA, 37 °C
TSA, 40 °C
1670
1686
1801
1954
1970
100
0
m/z
1550
1600
1650
1700
1750
1800
1850
1900
1950
2000
