# Supplementary material for: Comprehensive analysis of clinical Burkholderia pseudomallei isolates demonstrates conservation of unique lipid A structure and TLR4-dependent innate immune activation
Source: PLoS Negl Trop Dis. 2018 Feb 23;12(2):e0006287. doi: 10.1371/journal.pntd.0006287 (PMC5842036; doi:10.1371/journal.pntd.0006287)
Supplement: S1 Table — (PDF) [file pntd.0006287.s007.pdf]

S1 Table. *B. pseudomallei* wild type and mutant strains used in this study

Strain	Characteristic	LPS type	Reference
K96243	Wild type	A	[1]
K96243 Δ*wbiD*	Mutant defective in OPS synthesis	Rough	[2]
K96243 Δ*wbiA*	Mutant defective in acetylation of OPS	A	[2]
K96243 Δ*oacA*	Mutant defective in acetylation of OPS	A	[2]
4095a	Clinical strain from melioidosis patient	A	[2]
4095a Δ*wcbB*	Mutant defective in CPS synthesis	A	[2]

1. Holden MT, Titball RW, Peacock SJ, Cerdeno-Tarraga AM, Atkins T, Crossman LC, et al. Genomic plasticity of the causative agent of melioidosis, *Burkholderia pseudomallei*. Proceedings of the National Academy of Sciences of the United States of America. 2004;101(39):14240-5. doi: 10.1073/pnas.0403302101.

2. Wikraiphat C, Saiprom N, Tandhavanant S, Heiss C, Azadi P, Wongsuvan G, et al. Colony morphology variation of *Burkholderia pseudomallei* is associated with antigenic variation and O-polysaccharide modification. Infection and immunity. 2015;83(5):2127-38. doi: 10.1128/IAI.02785-14.
