# Supplementary material for: Comprehensive analysis of clinical Burkholderia pseudomallei isolates demonstrates conservation of unique lipid A structure and TLR4-dependent innate immune activation
Source: PLoS Negl Trop Dis. 2018 Feb 23;12(2):e0006287. doi: 10.1371/journal.pntd.0006287 (PMC5842036; doi:10.1371/journal.pntd.0006287)
Supplement: S2 Table — (PDF) [file pntd.0006287.s008.pdf]

S2 Table. Clinical isolates of *B. pseudomallei* from 68 Thai patients with episode of relapse and date of isolation and specimen types

Patient No.	Primary			Relapse		
	Strain	Date of isolation	Specimen type	Strain	Date of isolation	Specimen type
1	206a	22-Oct-1986	Blood	242a	15-May-1987	Blood
2	226a	19-Dec-1986	Wound swab	240a	28-Apr-1987	Blood
3	257a	2-Jul-1987	Pus	257c	14-Oct-1997	Pus
4	303b	26-Aug-1987	Blood	303d	2-Nov-1987	Blood
5	423a	4-Oct-1988	Blood	423g	15-Mar-1989	Blood
6	428a	18-Oct-1988	Sputum	428g	19-Jan-1989	Pus
7	430e	22-Oct-1988	Blood	454a	24-Jan-1989	Blood
8	436b	2-Nov-1988	Blood	436e	31-May-1989	Splenic aspirate
9	443a	10-Dec-1988	Pleural fluid	443d	23-Jun-1989	Pleural fluid
10	448a	20-Dec-1988	Blood	448c	4-Jan-1989	Blood
11	479a	8-Jun-1989	Sputum	479e	6-Oct-1989	Sputum
12	480a	7-Jun-1989	Lung	480c	2-Sep-1990	Pleural fluid
13	504a	16-Jul-1989	Blood	504d	24-Sep-1989	Blood
14	596a	27-Oct-1989	Blood	596c	7-Mar-1990	Wound swab
15	646a	3-May-1990	Sputum	646b	3-Jun-1990	Sputum
16	757a	11-Dec-1990	Liver aspirate	757c	5-May-1995	Blood
17	763a	5-Jan-1991	Pleural fluid	763b	1-May-1991	Sputum
18	770b	25-Jan-1991	Sputum	770c	26-Nov-1991	Sputum
19	845a	8-Sep-1991	Rectal swab	845d	15-Jan-1992	Blood
20	855e	5-Oct-1991	Rectal swab	855h	22-Apr-1994	Wound swab
21	1066a	15-Jul-1993	Liver aspirate	1066b	23-Aug-1996	Synovial fluid
22	1119a	14-Sep-1993	Pleural fluid	1119d	5-Dec-1993	Wound swab
23	1143a	8-Oct-1993	Liver aspirate	1143c	20-Jul-2001	Splenic aspirate
24	1165a	30-Oct-1993	Sputum	1165b	18-Dec-1993	Sputum
25	1189a	15-Jan-1994	Liver aspirate	1189b	27-Sep-1995	Liver aspirate

26	1210a	7-May-1994	Blood	1210c	23-Dec-1994	Blood
27	1219a	26-May-1994	Wound swab	1219b	18-Oct-1994	Urine
28	1234a	30-Jun-1994	Urine	1234b	7-Jan-1995	Urine
29	1237a	1-Jul-1994	Blood	1237d	5-Aug-1994	Sputum
30	1303b	8-Oct-1994	Blood	1303d	28-Nov-1994	Pus
31	1304a	11-Oct-1994	Liver aspirate	1304b	16-Dec-1994	Liver aspirate
32	1338a	18-Dec-1994	Liver aspirate	1338c	20-Dec-1997	Blood
33	1400b	16-Jul-1995	Blood	1400e	27-Jun-1996	Blood
34	1401a	12-Jul-1995	Blood	1401b	11-Aug-1995	Blood
35	1463a	4-Sep-1995	Blood	1463b	27-Feb-1996	Blood
36	1479a	21-Sep-1995	Urine	1479b	28-Jun-2001	Urine
37	1494a	13-Oct-1995	Wound swab	1494e	16-Aug-1996	Blood
38	1495a	13-Oct-1995	Splenic aspirate	1495b	12-Jun-1999	Wound swab
39	1501a	28-Oct-1995	Wound swab	1501c	4-May-1999	Pus
40	1577a	3-Jul-1996	Sputum	1577b	18-Mar-1997	Blood
41	1620a	5-Sep-1996	Blood	1620d	21-Jan-1998	Pus
42	1628b	20-Sep-1996	Blood	1628c	22-Aug-0202	Blood
43	1634a	20-Sep-1996	Blood	1634b	5-Mar-1999	Blood
44	1660a	18-Oct-1996	Pleural fluid	1660c	10-Apr-2001	Pleural fluid
45	1686a	13-Nov-1996	Blood	1686b	22-Feb-1997	Urine
46	1690a	22-Nov-1996	Blood	1690c	3-Jun-1999	Blood
47	1698a	12-Dec-1996	Liver aspirate	1698b	3-Feb-1997	Liver aspirate
48	1699a	13-Dec-1996	Pus	1699b	9-Oct-1997	Pus
49	1790a	25-Jul-1997	Throat swab	1790b	23-Aug-1997	Sputum
50	1897a	18-Dec-1997	Blood	1897d	20-Mar-1998	Blood
51	1923a	18-Mar-1998	Sputum	1923b	28-Jul-1998	Sputum
52	2029b	7-Aug-1998	Blood	2029e	27-Aug-1999	Blood
53	2033a	7-Aug-1998	Wound swab	2033c	2-Sep-1998	Wound swab
54	2310a	16-Jul-1999	Blood	2310c	23-Jul-2002	Blood
55	2507a	23-Jul-2000	Pus	2507d	22-May-2002	Liver aspirate

56	2622a	27-Feb-2001	Blood	2622e	9-Dec-2002	Blood
57	2624a	6-Mar-2001	Blood	2624b	6-Aug-2002	Wound swab
58	2644a	27-May-2001	Blood	2644e	9-Jun-2002	Blood
59	2817a	25-May-2002	Blood	2817b	11-Jul-2002	Blood
60	2821a	30-May-2002	Blood	2821b	16-Aug-2002	Urine
61	2831a	19-Jun-2002	Sputum	2831b	16-Jul-2002	Sputum
62	2944b	15-Oct-2002	Blood	2944d	23-Dec-2002	Blood
63	2991b	31-May-2003	Blood	2991d	12-Mar-2004	Blood
64	3161a	7-Oct-2003	Wound swab	3161b	18-Nov-2004	Wound swab
65	3171a	14-Oct-2003	Blood	3171e	23-Jun-2004	Blood
66	3183a	25-Oct-2003	Splenic aspirate	3183b	28-Sep-2004	Blood
67	3192a	8-Nov-2003	Blood	3192b	10-Nov-2005	Blood
68	3299a	26-Jun-2004	Lung	3299b	10-Jan-2005	Sputum
