# Supplementary material for: Comprehensive analysis of clinical Burkholderia pseudomallei isolates demonstrates conservation of unique lipid A structure and TLR4-dependent innate immune activation
Source: PLoS Negl Trop Dis. 2018 Feb 23;12(2):e0006287. doi: 10.1371/journal.pntd.0006287 (PMC5842036; doi:10.1371/journal.pntd.0006287)
Supplement: S3 Table — (PDF) [file pntd.0006287.s009.pdf]

S3 Table. Clinical isolates of *B. pseudomallei* from 35 individual colonies of 7 clinical specimens used in this study

Colony No.	Strain	Date of isolation	Specimen type
1	H3921b	17-Jul-2006	
2	H3921b	17-Jul-2006	
3	H3921b	17-Jul-2006	Blood
4	H3921b	17-Jul-2006	
5	H3921b	17-Jul-2006	
1	H3921e	17-Jul-2006	
2	H3921e	17-Jul-2006	
3	H3921e	17-Jul-2006	tracheal suction
4	H3921e	17-Jul-2006	
5	H3921e	17-Jul-2006	
1	H3921d	17-Jul-2006	
2	H3921d	17-Jul-2006	
3	H3921d	17-Jul-2006	urine
4	H3921d	17-Jul-2006	
5	H3921d	17-Jul-2006	
1	H3921g	17-Jul-2006	
2	H3921g	17-Jul-2006	
3	H3921g	17-Jul-2006	pus from right leg
4	H3921g	17-Jul-2006	
5	H3921g	17-Jul-2006	
1	H3921f	17-Jul-2006	
2	H3921f	17-Jul-2006	pus from left leg
3	H3921f	17-Jul-2006	
4	H3921f	17-Jul-2006	
5	H3921f	17-Jul-2006	

1	H3921c	17-Jul-2006	pus from forehead
2	H3921c	17-Jul-2006	
3	H3921c	17-Jul-2006	
4	H3921c	17-Jul-2006	
5	H3921c	17-Jul-2006	
1	H3921h	18-Jul-2006	wound swab from thigh
2	H3921h	18-Jul-2006	
3	H3921h	18-Jul-2006	
4	H3921h	18-Jul-2006	
5	H3921h	18-Jul-2006	
